# Hepatitis C Virus Induced miR200c Down Modulates FAP-1, a Negative Regulator of Src Signaling and Promotes Hepatic Fibrosis

**DOI:** 10.1371/journal.pone.0070744

**Published:** 2013-08-12

**Authors:** Sabarinathan Ramachandran, Haseeb Ilias Basha, Nayan J. Sarma, Yiing Lin, Jeffrey S. Crippin, William C. Chapman, Thalachallour Mohanakumar

**Affiliations:** 1 Department of Surgery, Washington University School of Medicine, Saint Louis, Missouri, United States of America; 2 Department of Internal Medicine, Washington University School of Medicine, Saint Louis, Missouri, United States of America; 3 Department of Pathology and Immunology, Washington University School of Medicine, Saint Louis, Missouri, United States of America; Baylor College of Medicine, United States of America

## Abstract

Hepatitis C virus (HCV) induced liver disease is the leading indication for liver transplantation (LTx). Reinfection and accelerated development of fibrosis is a universal phenomenon following LTx. The molecular events that lead to fibrosis following HCV infection still remains poorly defined. In this study, we determined microRNA (miRNA) and mRNA expression profiles in livers from chronic HCV patients and normals using microarrays. Using Genego software and pathway finder we performed an interactive analysis to identify target genes that are modulated by miRNAs. 22 miRNAs were up regulated (>2 fold) and 35 miRNAs were down regulated (>2fold) compared to controls. Liver from HCV patients demonstrated increased expression of 306 genes (>3 fold) and reduced expression of 133 genes (>3 fold). Combinatorial analysis of the networks modulated by the miRNAs identified regulation of the phospholipase C pathway (miR200c, miR20b, and miR31through cellular proto-oncogene tyrosine-protein kinase Src (cSrc)), response to growth factors and hormones (miR141, miR107 and miR200c through peroxisome proliferator-activated receptor alpha and extracellular-signal-regulated kinases, and regulation of cellular proliferation (miR20b, miR10b, and miR141 through cyclin-dependent kinase inhibitor 1 or CDK-interacting protein 1 p21). Real time PCR (RT-PCR) validation of the miRNA in HCV infected livers demonstrated a 3.3 ±0.9 fold increase in miR200c. *In vitro* transfection of fibroblasts with miR200c resulted in a 2.2 fold reduction in expression of tyrosine-protein phosphatase non-receptor type 13 or FAS associated phosphatase 1 (FAP-1) and 2.3 fold increase in expression of cSrc. miR200c transfection resulted in significant increases in expression of collagen and fibroblast growth factor (2.8 and 3.4 fold, p<0.05). Therefore, we propose that HCV induced increased expression of miR200c can down modulate the expression of FAP1, a critical regulator of Src and MAP kinase pathway that play an important role in the production of fibrogenic growth factors and development of fibrosis.

## Introduction

Hepatic fibrosis is a complex biological process that is progressive in nature leading to the development of cirrhosis and later to the development of hepatocellular carcinoma. Hepatitis C infection is attributed to be involved in more than 40% of the chronic liver disease and remains the major reason for liver transplantation (LTx) in US and Europe [1]. Approximately 3% of the global population or 170 million people are infected with Hepatitis C virus (HCV) [2]. In the HCV infected transplant recipients reinfection of the transplanted liver is a universal phenomenon and the resulting fibrosis follow an accelerated progression [3].

Chronic liver injury results in inflammation, apoptotic cell death of the hepatocytes and initiation of a wound healing response that results in the development of fibrous deposition leading to development of fibrosis [4]. Myofibroblasts developed from the hepatic stellate cells have been shown to play a major role in the development of liver fibrosis [5]. Development of fibrosis following chronic HCV infection plays a pivotal role in loss of liver function resulting in end stage liver diseases. Increased expression of pro-fibrogenic growth factors including transforming growth factor-β (TGF-β), hyaluron and extra cellular matrix proteins has been correlated with the degree of fibrosis in the liver [6]. B cells infiltrating the liver and Th17 cells have been shown to be important sources of cytokine IL-6, which is involved in the differentiation of hepatic stellate cells into myofibroblasts, induction of fibroblast proliferation, and the increased synthesis of extracellular matrix collagens and metalloproteinase inhibitors [7]. Chronic inflammation leads to the activation of hepatic stellate cells to produce extracellular matrix proteins and growth factors that are involved in the increased proliferation of the fibroblasts as well as deposition of the fibrous scar leading to loss of liver function [8]. Increased levels of TGF-β [9], platelet derived growth factor (PDGF) [10], fibroblast growth factor (FGF), hepatocyte growth factor [11], and vascular endothelial growth factor (VEGF) [12] have been reported in the chronic HCV individuals and are thought to play a crucial role in the fibroproliferative cascade leading to development of fibrosis. Upon binding of the fibrogenic growth factors (TGF-β and FGF) to their cognate receptor on the target cells results in the activation of cSrc, Mitogen-activated protein kinase (MAPK) and Extracellular Signal-regulated Kinase (ERK) leading to increased production of growth factors and extracellular matrix proteins resulting in development of fibrosis [13], [14]. However the mechanisms by which HCV infection modulate the process of fibrosis still remains poorly defined.

Non coding RNAs have been shown to participate in a variety of cell regulatory events [15]. microRNAs (miRNAs) are a family of 21–25 nucleotide RNAs that were initially discovered as small temporal RNAs regulating developmental events in C. elegans [16]. There are hundreds of miRNAs that have been described in various species and they have been implicated in negatively regulating gene expression at the post transcriptional level [17]. They have diverse expression patterns and regulate many events in development and cell physiology [18]. The mechanisms by which miRNA regulate gene expression are still poorly defined, but the finding of miRNAs feeding into RNA interference pathway has been important in understanding their biological functions. miRNAs have been shown to play an important role in immune evasion, cell cycle regulation and in cancer progression [19]. HCV infection has been shown to modulate miRNA levels in hepatocytes that are involved in HCV entry, propagation and immune evasion [20].

In this study we demonstrate that HCV infection results in the modulation of miRNA and these mRNA changes in the liver can promote growth factor signaling, cell proliferation and survival. We also demonstrate that increased levels of miR200c in the HCV infected livers results in the inhibition of FAS associated phosphatase 1 (FAP-1), a protein tyrosine phosphatase. FAP-1, a multimodule tyrosine phosphatase regulates the function of Src kinase by dephosphorylation that plays an essential role in activation of the down-stream signaling cascades of growth factors. We propose that these events will result in increased production of pro-fibrogenic growth factors and products of extracellular matrix (ECM) that are involved in the development of hepatic fibrosis following HCV reinfection.

## Materials and Methods

### Ethics Statement

All of the human studies were approved by the human research protection committee at Washington University (protocol 201104075) and patients were enrolled after written informed consent was obtained.

### Patient population

Patients with documented HCV infection confirmed by HCV RNA quantitative polymerase chain reaction (PCR) assays with a lower detection limit of 50 IU/mL (Cobas Amplicor, Roche Diagnostics) were enrolled in this study. Patients with Hepatitis B virus (HBV) or HIV co-infection were excluded from the study. Liver biopsies obtained from patients infected with HCV during routine follow-up were used in this study. Liver biopsies were scored for fibrosis with the Scheuer staging system. Liver biopsies procured from donor livers prior to transplantation were used as normal controls. Liver biopsies obtained from 10 HCV patients with absent/minimal evidence of allograft fibrosis (S0–S2) and varying grades of allograft inflammation (G0–G4) and 10 liver biopsies obtained from patients with non-alcoholic steatohepatitis were also procured. No donor organs were obtained from executed prisoners or other institutionalized persons. The demographic profile of the patient population is presented in Table 1. Serum samples were also collected at the time of liver biopsy.

**Table 1:** Demographics of the patients.

	HCV Fibrosis	HCV Inflammation	NASH
**Age (yrs)**	**49.1±9.8**	**52.3± 7.4**	**50.1±5.7**
**Gender (% M:F)**	**60: 40**	**60: 40**	**50: 50**
**Ethnicity (%)**CaucasianAfro-American	**80** **20**	**70** **30**	**70** **30**
**Fibrosis stage(S)**	**S2–S4**	**S0**	**3–4**
**Inflammatory Grade (G)**	**G2–G4**	**G2–G4**	**N/A**
**HCV genotype**1Non-1	**80** **20**	**80** **20**	**N/A**
**HCV load (log IU/ml)**	**13.3±2.6**	**12.4±5.6**	**N/A**
**ALT [Mean(range)] IU/ml**	**111(45-326)**	**126 (67-367)**	**72 (36-315)**
**AST [Mean(range)] IU/ml**	**103(26-271)**	**98 (54-278)**	**67 (33-214)**

### Gene array and miRNA array analysis

Liver biopsy that were snap frozen obtained from HCV or control normal liver, were used to isolate RNA using Trizol reagent (Invitrogen, Carlsbad, CA) as per manufacturer's protocol. Resulting RNA was quantified by A260 and A280 readings using a Nanodrop spectrophotometer (Nanodrop Technologies, Wilmington DE) and qualitatively assessed using a BioAnalyzer 2100 and RNANanoChip assay (Agilent Technologies, Palo Alto, CA). All nucleic acid extraction procedures were performed by the Siteman Cancer Center Tissue Procurement Core (Washington University). miRNA microarray data and mRNA microarray data for samples was generated with Illumina Bead arrays in the Siteman Cancer Center Molecular and Genomic Analysis Core (Washington University) starting with 1 μg of quality controlled total cellular RNA as per standard protocols. Both miRNA and mRNA data were analyzed in Partek Genomic Suite (Version 6.6) following background subtraction and quantile normalization in Illumina Genome Studio platform. Control probes and probe sets that were called “Absent” across all samples were removed prior to any analysis. Analysis of Variance (ANOVA) algorithm as implemented in Partek was used to compare the miRNA and mRNA expression pattern in liver biopsies between the HCV patients and normal donors at statistical significance p< 0.05. The differentially expressed miRNA in the mRNA was used to identify the gene ontological processes as well as the signaling networks targeted using the Genego software.

### Validation of the array data using quantitative RT-PCR

Results from the miRNA and the mRNA arrays were validated using quantitative RT-PCR. Liver biopsies from 10 patients with HCV with grade III/IV fibrosis, 10 patients with HCV infection but no fibrosis and 10 patients with nonalcoholic steatohepatitis were used to validate the array data.

### Isolation of primary human liver fibroblasts

Primary human liver fibroblasts were isolated from donor liver (n = 2) as described earlier [21] with certain modifications. Briefly, the liver tissue (0.5×5 mm) was washed twice in RPMI, minced with surgical blades under aseptic conditions and digested overnight with collagenase (1 mg/mL), filtered to remove undigested tissue remnants and layered on collagen 1-coated plates and cultured for 7 days at 37°C in 5% CO_2_ and harvested by trypsinization for further studies.

### Functional studies

In order to determine the function of miR-200c, we transfected 1×106 human liver fibroblasts (ATCC, Manassas, VA) or isolated primary human liver fibroblasts with 200 nM of Pre-miR™ miRNA Precursor -miR200c, -miR141 or scrambled miRNA (Invitrogen, Carlsbad, CA) using Lipofectamine® RNAiMAX (Invitrogen, Carlsbad, CA). Transfected cells were stimulated with 50 ng/ml of recombinant human TGF-β (R&D systems) for 24 hrs. Levels of miR-200c, FAP-1 and FGF were analyzed by quantitative RT-PCR using Taqman pre-developed assays (Applied Biosystems, Foster City, CA). Liver fibroblasts were cotransfected with 50 nM of *mir*Vana® miRNA inhibitor and 200 nM of pre-miR miRNA to discern the role of individual miRNA in modulation of the target gene. Cells were treated with Src inhibitor-1 (10 µM, Sigma-Aldrich, St Louis, MO) or controls during stimulation with TGF-β to discern the role of Src signaling. Protein levels of activated cSrc and FAP-1 were analyzed by western blot analysis.

### RT-PCR analysis

Total RNA, including miRNAs was extracted using the Trizol reagent (Invitrogen, Carlsbad, CA). miRNA expression was measured by quantitative PCR using Biorad iCycler RT-PCR machine. miRNA TaqMan primers and probes were purchased from Applied Biosystems (Foster City, CA). The small RNA U6b was used as an endogenous control and relative miRNA quantity was calculated by the ΔΔCt method. Gene expression was measured by quantitative PCR using Biorad iCycler RT-PCR machine. Gene expression assay TaqMan primers and probes, TaqMan® One-Step RT-PCR Master Mix were purchased from Applied Biosystems (Foster City, CA). For gene expression studies, the human actin B (ACTB) was used as an endogenous control and the relative gene expression levels were calculated by the ΔΔCt method and the fold changes in the control and experimental groups were compared by student t test.

### Protein expression

Expression levels of the genes at the protein level were determined by Western blotting as previously described. The primary antibodies used in this study are cSrc (Cell Signaling), FAP1 (Cell Signaling), and β-Actin (Santa Cruz Biotechnology). The western blots were developed with SuperSignal West Pico Chemiluminescent kit (Pierce Biotechnology, Rockford, IL). Densitometric analysis of the bands was performed using QuantityOne software (Biorad, CA) and the expression values in the experimental and control (untransfected) groups were compared by student t test.

### TGF-β ELISA

Circulating levels of TGF-β in the serum of chronic HCV patients and normal subjects were analyzed using Human TGF-β 1 Quantikine ELISA Kit (R&D systems, Minneapolis, MN) as per manufacturers' instruction. Briefly, to activate the latent TGF-β to immunoreactive TGF-β, 40 µL of serum sample was added to 20 µL of1N HCl and incubated for 10 minutes at room temperature and neutralized by adding 20 µL of 1.2N NaOH/0.5M HEPES. The samples were further diluted 20 fold with calibrator diluent RD5-53. 50 µL of assay diluent and 50 µL of diluted serum sample or standards were added to each well and incubated for 2 hours at room temperature. The wells were washed (4X) and incubated with 100 µL of TGF-β conjugate and incubated for 2 hours at room temperature. The plates were developed with 100 µL of substrate solution for 30 minutes and stopped with 100 µL of stop solution and the optical density was measured at 450 nm and corrected by measurements at 540 nm. A standard curve was generated using 4 parameter logistic curve fit and used to calculate the concentrations in the serum sample.

## Results

### HCV infection results in modulation of miRNA profile

In order to determine the effect of chronic HCV infection in the expression of miRNAs we analyzed the miRNA expression profile in liver biopsies of chronic HCV patients and compared them to normal healthy livers procured for transplantation. Total RNA was isolated from the liver biopsies and miRNA expression analysis was done using the Illumina V2 human array that contains (1146) probe sets. Anova analysis of the miRNA levels demonstrated significant differences in the miRNA profiles between the two groups (p<0.05). Changes in expression level of 2-fold or more in the HCV samples compared with the normal controls were considered to be significant. The data discussed in this publication have been deposited in NCBI's Gene Expression Omnibus (Edgar *et al*., 2002) and are accessible through GEO Series accession number GSE38226 (http://www.ncbi.nlm.nih.gov/geo/query/acc.cgi?acc= GSE38226). In 6 of the 8 samples that demonstrated a significant difference we identified 22 miRNAs that are up regulated (>2 fold) and 35 miRNAs that are down regulated (>2 fold) in the liver of HCV patients compared to controls (Table 2). A significant increase in the expression of miR144 (11.77 fold, p = 0.022), miR31 (7.87 fold, p = 0.002), miR141 (3.40 fold, p = 0.009) and miR200c (2.95 fold, p = 0.02) were observed in the chronic HCV infected liver compared to the controls. Similarly the levels of miR802 (4.97 fold, p = 0.001), miR556-3p (4.5 fold, p = 0.0042) and miR615-5p (3.67 fold, p<0.001) were significantly down regulated in the livers from chronic HCV patients compared to the controls. The miRNAs that are modulated in chronic HCV liver is presented in Table 2. Gene ontological grouping of the differentially expressed miRNAs identified phospholipase C activating G protein coupled receptor signaling pathway (miR220c, miR20b and miR31), response to endogenous stimulus (miR141, miR107, miR200c), regulation of cellular processes (miR20b, miR10b and miR141), response to wound healing (miR34c) and leucocyte migration (miR483) as the major gene ontological processes that could be modulated by the differentially expressed miRNAs in the chronic HCV infected liver. These results are consistent with the possibility that the differentially expressed miRNAs in the HCV infected liver may play a significant role in the development of fibrosis.

**Table 2:** miRNA expression profile in chronic HCV.

ID	p-value	Fold Change	ID2	p-value	Fold Change
hsa-miR-144	0.0228226	11.7742	hsa-miR-431	0.0357257	−2.00977
hsa-miR-31*	0.002818	7.86888	hsa-miR-651	1.96E-06	-2.0156
hsa-miR-486-3p	0.0186293	7.46136	hsa-miR-1275	0.00111948	-2.07652
hsa-miR-144:9.1	0.030492	6.98866	hsa-miR-616*	0.00798456	-2.10282
hsa-miR-31	0.00681662	5.08077	hsa-miR-196a*	0.0335879	-2.11588
HS_176	0.018694	4.73535	hsa-miR-193b*	0.00722542	-2.13796
hsa-miR-708	0.00867611	3.83595	hsa-miR-24-1*	5.42E-06	-2.16046
hsa-miR-10b	0.0125468	3.69579	solexa-15-44487	0.0468131	-2.17189
hsa-miR-141	0.00995113	3.40592	hsa-miR-128a:9.1	0.00246633	-2.24699
hsa-miR-182	0.0116925	3.38087	hsa-miR-548j	0.00279194	-2.25898
hsa-miR-34c-3p	0.0320449	2.97359	hsa-miR-483-5p	0.00560094	-2.28867
hsa-miR-200c	0.0245857	2.94861	hsa-miR-128b:9.1	0.0107908	-2.31064
hsa-miR-486-5p	0.00281192	2.79836	hsa-miR-548b-3p	0.0134523	-2.31549
HS_97	0.0265193	2.66399	hsa-miR-449a	0.007856	-2.38192
hsa-miR-138	0.0375211	2.62481	hsa-miR-148a*	8.88E-05	-2.46658
HS_263.1	0.00630278	2.61126	hsa-miR-23b*	0.000776048	-2.4976
hsa-miR-218	0.0485095	2.59988	hsa-miR-194*	7.52E-05	-2.51704
hsa-miR-34b*	0.00826268	2.31809	hsa-miR-107	0.0151007	-2.53139
hsa-miR-125b-1*	0.00738561	2.22584	hsa-miR-548o	0.0154111	-2.5446
hsa-miR-20b	0.0282687	2.21857	hsa-miR-92a-1*	0.005961	-2.58038
HS_203	0.00936761	2.15614	hsa-miR-616	0.000142243	-2.73623
hsa-miR-214*	0.0449169	2.09365	hsa-miR-592	0.00275174	-2.78955
			hsa-miR-1258	0.000234539	-2.8273
			hsa-miR-1268	0.00781389	-3.16454
			hsa-miR-130b*	0.00123395	-3.19733
			hsa-miR-556-5p	0.00471186	-3.25349
			hsa-miR-33b*	2.55E-05	-3.55305
			hsa-miR-615-5p	3.13E-06	-3.67112
			hsa-miR-556-3p	0.0042323	-4.50595
			hsa-miR-802	0.00174168	-4.97243

### Identification of the target genes that are modulated by chronic HCV infection

To identify the target genes that are modulated by miRNAs, we performed a whole genome microarray on the liver biopsies from 8 chronic HCV patients and compared them to normal livers. Total RNA was isolated from the liver biopsies and mRNA expression analysis was done using the Illumina Human HT-12 v3 Expression BeadChips array that contains (48000) probe sets. Gene expression levels in the samples were normalized based on the 16s ribosomal RNAs and housekeeping gene expression. Using PARTEK analysis software we analyzed the expression levels of the genes. Changes in gene expression level of 2-fold or more in the HCV samples compared with the normal controls were considered to be significant. Chronic HCV patients had greater than two fold increases in the expression of 785 genes and greater than 2 fold reduction in the expression of 533 genes. Functional grouping of the modulated genes (Table 3) identified components of the cell cycle regulation/apoptosis pathways (NFkB, Bcl2 and cIAP, cSRC, BRCA1 and TCF4), immune function (HLA-A, IRF-1, MxA, MGAT2 and ASCL1), and cellular response to growth factor stimulus (ILK, cSRC, STAT3) as the major gene ontological processes modulated by HCV infection. These results support that the differentially expressed genes in the HCV livers can influence many of the major biological processes.

**Table 3:** Transcriptional modulation of biological networks in chronic HCV infection.

Key network objects	GO Processes	Seed nodes	p-Value	zScore	gScore
NF-kB, Bcl-2, c-IAP1, c-IAP2, Leptin receptor	regulation of programmed cell death (80.0%), positive regulation of cellular process (98.0%)	11	8.43e-09	9.65	172.15
c-Src, Brca1, DAB2, TCF7L2 (TCF4), NF-kB2 (p100)	regulation of cell proliferation (66.0%), Wnt receptor signaling pathway (40.0%),	10	5.93e-08	8.99	57.74
ILK, c-Src, STAT3, Neuropilin-1, SHIP	transmembrane receptor protein tyrosine kinase signaling pathway (60.7%), cellular response to growth factor stimulus (51.8%),	11	1.86e-08	9.24	21.74
Alpha1-globin, Phox1 (PRRX1), PGHD, miR-107, SLIT2	regulation of response to stimulus (71.1%), regulation of cell death (53.3%), regulation of signaling (60.0%)	19	7.54e-20	18.20	19.45
STK39, HAI-2, ITM2C, PDCD4, P4HA1	multicellular organismal response to stress (16.0%)	16	7.22e-16	15.52	19.27
HLA-A, IRF1, MxA, MGAT2, ACSL1	antigen processing and presentation of endogenous peptide antigen (24.0%), positive regulation of T cell mediated cytotoxicity (24.0%)	13	1.02e-11	12.28	18.53
WARS, ICAM3, STAT1, alpha- D/beta-2 integrin, ITGAX	response to other organism (43.6%), immune response (43.6%), defense response to virus (20.5%)	16	3.29e-16	15.90	17.15
SLC34A1, COL6A2, F-spondin, IMPA2, SMOC2	Gamma-aminobutyric acid signaling pathway (27.1%)	17	4.09e-17	16.36	16.36
NKCC1, ACACB, BETA-IG-H3, PCSK9, HCD2	Second-messenger-mediated signaling (21.7%)	16	1.52e-15	15.17	15.17
Ephrin-B receptor 6, MIR (Idol), PTPR-epsilon	Ephrin enzyme linked receptor protein signaling pathway (37.5%)	12	1.79e-10	11.26	13.76

### miRNA mediated changes in gene expression during HCV infection affects cellular pathways implicated in chronic HCV infection

**Figure 1:**
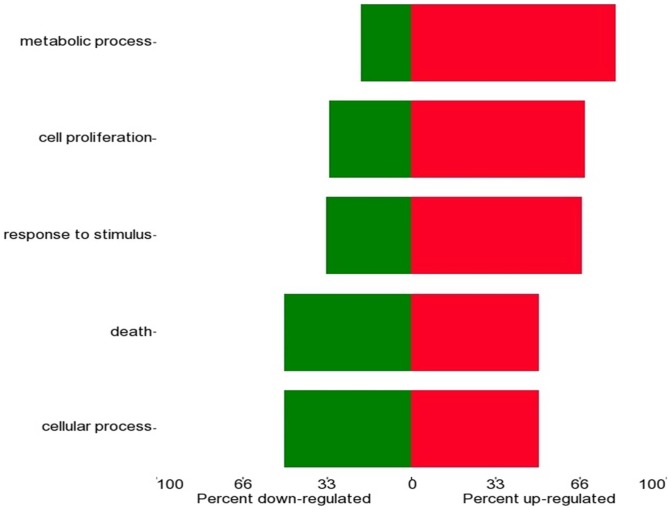
Pathways modulated in chronic HCV. RNA from liver biopsies from 8 HCV patients and normal donor livers were isolated using Trizol reagent and miRNA expression profile in the samples were analyzed using Illumina Bead array. The differentially expressed miRNA identified using Partek analysis were grouped based on the biological pathways that could be modulated.

In order to identify the genes that are targeted by the modulated miRNAs in the chronic HCV infected liver we combined the differentially expressed miRNAs and the differentially expressed mRNAs in the Partek miRNA analysis using the Targetscan 5.0 database. Potential interactions, as many as 289, were defined between the differentially expressed miRNAs and mRNAs identified in the HCV infected liver (Table 4). In order to delineate the biological significance we used the combined miRNA and mRNA pairs to build networks using Genego software. The biological processes that are modulated by chronic HCV infection are presented in figure 1. Among all the biological processes, multiple hits were observed in the “cellular response to endogenous stimulus through growth factors” pathways suggesting that HCV infection results in differential expression of miRNAs that modulate cellular response to endogenous stimulus including growth factors which can promote development of fibrosis.

**Table 4:** mRNAs targeted by the modulated miRNA in chronic HCV.

ID	SYMBOL
**hsa-miR-144**	ZNF532, MYH10, COL5A2, DUSP5, ALDH1A3, DUSP1, CUGBP2, FLJ20160, CEP135, RUNX1, PDCD4, PRRX1, ARHGEF3, FOSB, PAPPA, TGIF1, PCDH18
**hsa-miR-486-3p**	CLIP3, STEAP3, MLLT6, NAMPT
**hsa-miR-31**	COL5A1, PC, STX3, KLF13
**hsa-miR-708**	DKK3, HNT, DKK3
**hsa-miR-10b**	TPM4, MAPRE1, CUGBP2, KCNA6, TPM4
**hsa-miR-141**	MOBKL2B, MYH10, DOCK4, TCF4, GPHN, SPG3A, SCD5, RUNX1, PDCD4, GLS, PAPPA, FRMD6
**hsa-miR-182**	ANXA11, COL5A1, LAMC1, PC, MOBKL2B, ZNF532, CMTM7, MAPRE1, F13A1,ANXA11, FZD5, LIPG, DOCK4, NAP1L1, CUGBP2, PLOD2, HBEGF, DKFZP564O0823, INSIG1, DDEF1, CLPTM1L, KLF13, SAMD5, ARHGEF3, EVI1, PHLDB1,PAPPA, MYADM, ATOH8, BCL2, RAB34, THBS2, NAMPT, PCDH18
**hsa-miR-34c-3p**	CD34, GLS, PPP1R1A, SLITRK3, MTUS1
**hsa-miR-200c**	PTPN13, SNAP25, LFNG, C5orf13, ABAT, LAMC1, C3orf23, ZNF532, LEPR, CDR2L, MAPRE1, NIN, DOCK4, DUSP1, CUGBP2, TCF4, DDIT4, DDEF1, CREB5, KLF13, ABCC9, OSR1, ARHGEF3, KLF4, TIMP2, SGIP1, PHLDB1, KIAA0644, NIN, BCL2, MTUS1, COL4A3, LPAR1, HPS5, FRMD6
**hsa-miR-486-5p**	EPHA3, CUGBP2, OLFM4
**hsa-miR-138**	SNAP25, SNCAIP, ADAMTS5, SCRN1, MAPRE1, TCF4, GPHN, VIM, HK1, SH3GL2, ARHGEF3, MLLT6, RASL12, PAPPA
**hsa-miR-218**	ABAT, PLEK, DUSP5, TCF4, ACSL1, INSIG1, BRCA1
**hsa-miR-20b**	SAR1B, OSBPL5, PLXNA1, ADAMTS5, UGDH, PTPN3, ZNF532, ARHGEF10, MAPRE1, FAM129A, NIN, DOCK4, FURIN, IRF1, CUGBP2, TCF4, NR4A3, FJX1, SCD5, CREB5, RUNX1, DPYSL2, PRRX1, EGR2, OSR1, SLITRK3, ARHGEF3, TIMP2, MLLT6, GLIS3, NR4A2, NIN, COL4A3, DAB2, HPS5, FRMD6
**hsa-miR-431**	COL12A1
**hsa-miR-651**	BCL2, KDELR2, DHCR24, ZHX3
**hsa-miR-548j**	MAB21L2, ZFPM2, ADARB1, CD44, STAT3, CSNK1E, CD44, SMOC2, MXRA7, FLRT2, RALGPS1, CAV1
**hsa-miR-548b-3p**	DAB2, BCL11A, FGF9, NAMPT, PPP2R1B
**hsa-miR-449a**	MYH9, ANK3, RALGDS, BCL2, EI24, NR4A2, SIDT1, SLC7A6, SGSM2, CREB5, PDE4B, CNTNAP1, COL12A1, PDE4B, VCL, JAG1, CACNB3, GAS1, PID1, MMAB SLC12A2, SOX4, PODXL, RALGPS1, SEMA4B
**hsa-miR-107**	ST8SIA4, THY1, MYH9, ANK3, ZFPM2, BCL11A, RNF19A, SVEP1, CREB5, CNTNAP1, HIPK2, VCL, ZHX3, GPR124, SRGAP1
**hsa-miR-548o**	C5orf29, MARCKS, ST3GAL6, PMEPA1, DAB2, MEF2C, ANTXR1, JAG1, FLRT2, MRPL22,
**hsa-miR-616**	NTN1, ANTXR1, MMAB
**hsa-miR-592**	ABCA1, BHLHB3, CYP1B1
**hsa-miR-1258**	YWHAZ, DDEF1, SGSM2
**hsa-miR-556-5p**	CREB5, HIPK2, RCC2, LHFPL3
**hsa-miR-615-5p**	BRCA1, CD3E, DBN1, TBC1D10C, OAS3, MPDU1
**hsa-miR-556-3p**	COL4A1, B3GALNT1, DTX3L, TGFBI, IMPA2, MSRB3, NAMPT, CSNK1E, HIPK2, NCK2
**hsa-miR-802**	YWHAZ, RTN1, ZFPM2, CDH11, TSHZ3, HIPK2, LHFPL3, TADA1L

**Figure 2:**
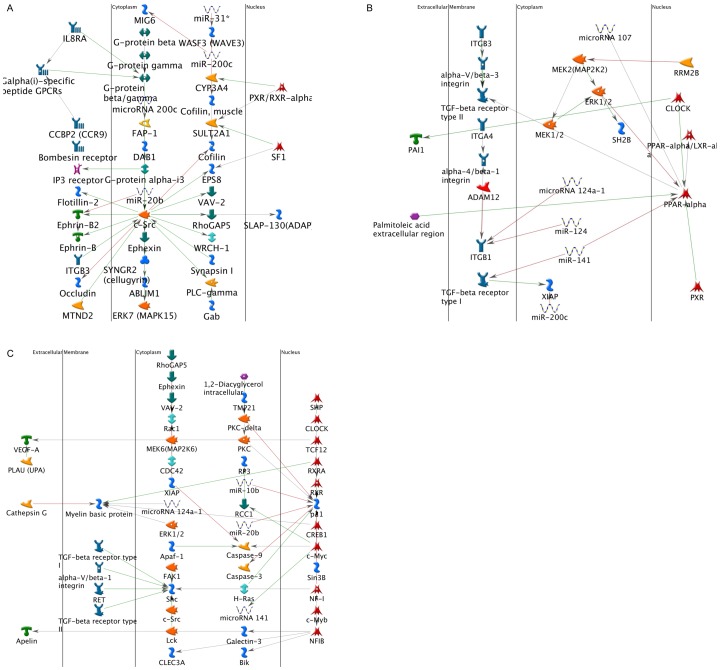
Biological networks modulated by the differentially expressed miRNA in chronic HCV. The biological networks modulated by differentially expressed miRNA following HCV infection was analyzed using the Genego software. Genego analysis of the networks identified: a) regulation of the phospholipase C pathway (miR200c, miR20b, and miR31 through cSrc); b) response to endogenous stimulus of growth factors and hormones (miR141, miR107 and miR200c through PPAR-α and ERK); and c) regulation of cellular proliferation (miR20b, miR10b, and miR141 through p21) as the major biological pathways that are impacted.

Analysis of the networks modulated by the miRNAs (figure 2a, b, c) identified regulation of the phospholipase C pathway (miR200c, miR20b, and miR31 through cSrc) (figure 2a), response to endogenous stimulus of growth factors and hormones (miR141, miR107 and miR200c through Peroxisome proliferator-activated receptors-α *(*PPAR-α) and ERK (figure 2b)), and regulation of cellular proliferation (miR20b, miR10b, and miR141 through p21) (figure 2c). The list of putative networks that are modulated by the miRNAs in the chronic HCV infected liver is presented in Table 5.

**Table 5:** Gene Ontological process affected by miRNAs.

Key network objects	GO Processes	Total nodes	Seed nodes	p Value	zScore	gScore
miR 200c, microRNA 200c, miR 20b, miR 31*, c Src	phospholipase C activating G protein coupled receptor signaling pathway (21.3%), cell communication (76.6%), signal transduction (72.3%)	50	4	8.96e 08	19.56	19.56
miR 141, microRNA 107, miR 200c, PPAR alpha, ERK1/2	response to endogenous stimulus (58.3%), positive regulation of biological process (77.8%), positive regulation of cellular process (75.0%)	50	3	8.75e 06	14.93	14.93
miR 20b, miR 10b, microRNA 141, p21, Shc	developmental process (91.5%), anatomical structure development (87.2%), positive regulation of cellular process (80.9%), positive regulation of biological process (83.0%)	50	3	9.90e 06	14.62	14.62
microRNA 20b, miR 182, microRNA 31, CREB1, Bax	cellular response to chemical stimulus (51.1%), cellular response to organic substance (44.4%), response to inorganic substance (31.1%), response to organic substance (53.3%)	50	3	9.90e 06	14.62	14.62
miR 34c 3p, Beta catenin, CDK1 (p34), c Raf 1, GLUT4	positive regulation of cellular process (85.3%), positive regulation of macromolecule metabolic process (70.6%), positive regulation of biological process (85.3%)	38	1	3.07e 02	5.49	5.49
miR 34c 5p, c Myb, Histone H3, Collagen I, PLAU (UPA)	response to wounding (63.6%), wound healing (50.0%), coagulation (45.5%), blood coagulation (45.5%), hemostasis (45.5%)	50	1	3.71e 02	4.96	4.96
microRNA 483, FAK1, G protein alpha i family, NF I, eIF4E	leukocyte migration (36.7%), hemostasis (44.9%), neuron projection development (46.9%)	50	1	4.02e 02	4.74	4.74

### Increased TGF-β production in chronic HCV

**Figure 3:**
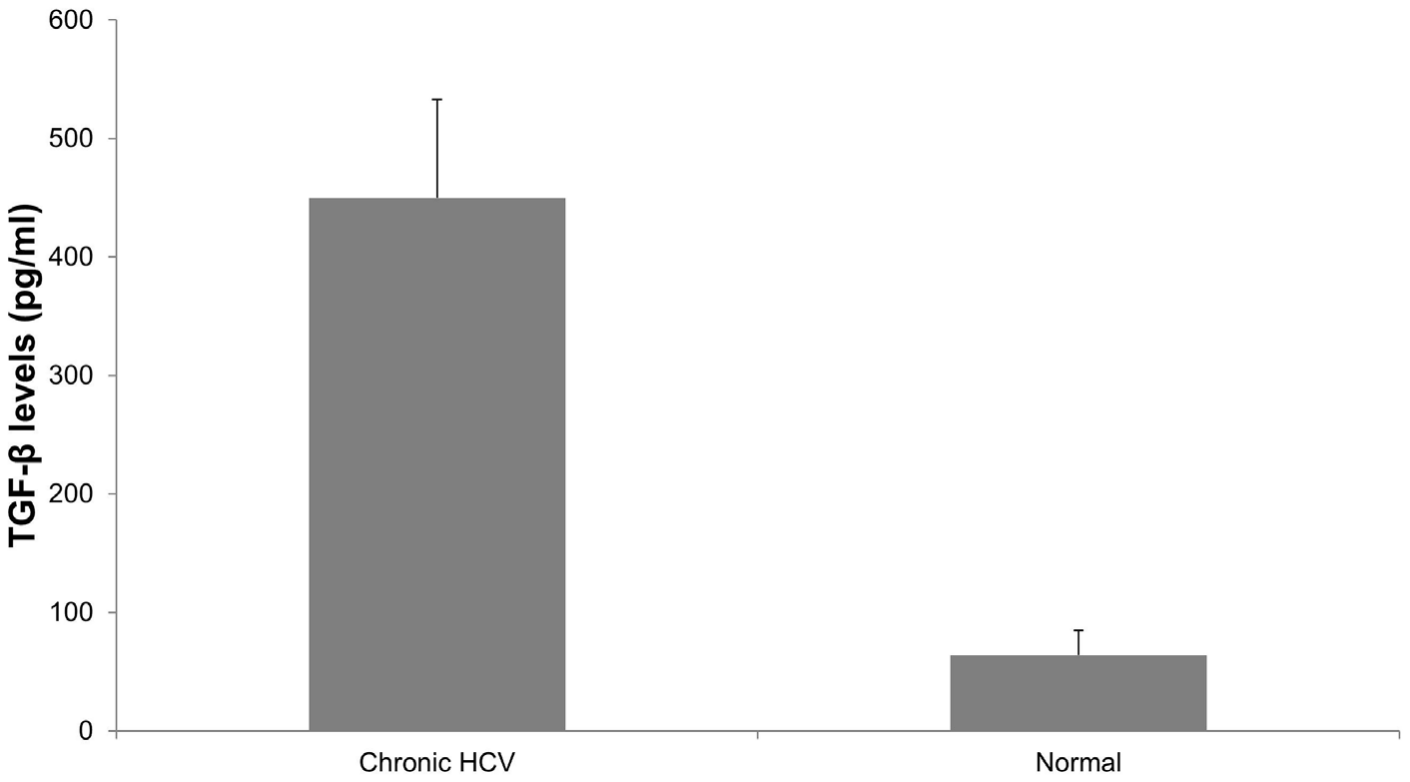
Increased TGF-β in serum of HCV patients. We measured the levels of TGF-β in the sera of chronic HCV patients and compared them to normal subjects using a TGF-β ELISA kit. The bars represent the mean ± SD levels of TGF-β concentration. The levels of TGF-β in the sera from chronic HCV patients were significantly elevated when compared to healthy controls (450 vs. 64 µg/ml, p<0.05).

It is well accepted that TGF-β plays a crucial role in the development and progression of fibrosis [22]. Therefore, we measured the levels of TGF-β in the sera of 20 chronic HCV and compared them to normal subjects using a TGF-β ELISA kit. As shown in figure 3, ELISA results demonstrated significant increases in the levels of TGF-β in the sera from chronic HCV patients when compared to healthy controls (450 vs. 64 ug/ml, p<0.05).

### Transfection of human liver fibroblasts with miR200c decreases expression of FAP-1

**Figure 4:**
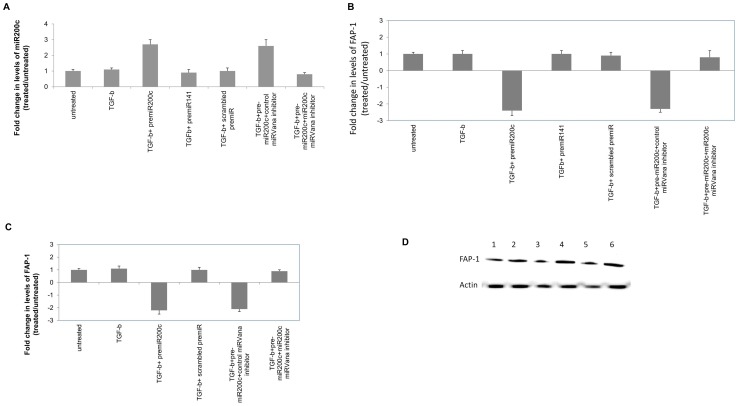
Increased miR200c decreases FAP-1 expression. Human liver fibroblasts were transfected with pre-miR200c miRNA or scrambled miRNA (200 nM) using Lipofectamine RNAiMax and stimulated with TGF-β (50 ng/mL). RNA was isolated using trizol reagent and expression levels of miR200c and FAP-1 were measured using pre-developed Taqman miRNA and mRNA assays respectively. The small RNA U6b (miRNA) and GAPDH (mRNA) was used as an endogenous control and relative levels was calculated by the ΔΔCt method. Bars represent the mean expression observed in 3 different experiments performed with 3 different fibroblasts. In order to further define the role of miR200c, we cotransfected fibroblasts with pre-miR200c and *mir*Vana® miRNA inhibitor for miR200c and analyzed for the expression levels of miR200c and FAP-1. Cotransfection with *mir*Vana® miRNA inhibitor (50 nM) resulted in restoration of the levels of miR200c and FAP-1 to the levels observed in the untreated fibroblasts. a) Relative expression levels of miR200c; b: Relative expression levels of FAP-1 at the mRNA level; c: Relative expression levels of FAP-1 at the protein level and d: Representative western blot analysis of FAP-1. Lanes 1) Fibroblasts; 2) Fibroblast + TGF-β; 3) Fibroblast + TGF-β+pre-miR-miR200c; 4) Fibroblast + TGF-β+ scrambled pre-miR; 5) Fibroblast + TGF-β+ pre-miR –miR200c+ *mir*Vana® miRNA inhibitor control; and 6) Fibroblast + TGF-β+ pre-miR –miR200c+ *mir*Vana® miRNA inhibitor miR200c.

Since analysis of the miRNA and mRNA expression profiles identified that there was a significant modulation of miR200c along with its target (FAP-1) and downstream signaling intermediates (cSRC, FGF, and extracellular matrix components) that can promote the development of fibrosis, the miR200c pathway was selected for further analysis. The miRNA analysis showed that miRNA-200c is up regulated (2.9 fold) in HCV patients compared to normal (Table 2). Computational target prediction of miR200C using Targetscan (Targetscan.org) identified FAP1 (Tyrosine-protein phosphatase non-receptor type 13, PTPN13) to be a putative target for translational silencing. In order to investigate the role of miR200c in the development of fibrosis, we transfected normal human liver fibroblasts (CRL11005, ATCC or 2 fibroblasts isolated from donor livers) with pre-miR200c, control miRNA or pre-miR141 (shares the 5′ seed sequence of miR200c) and expression of both miRNA-200C and FAP1 were determined by quantitative RT-PCR following stimulation with TGF-β. The miR200c results were normalized to small RNA U6b levels and FAP-1 levels were normalized with GAPDH expression. A 2.7 fold increase in the levels of miR200c was observed in the fibroblasts when transfected with miR200c, however, the levels of miR200c were unaffected when transfected with miR141 or scrambled miRNA (figure 4a). Transfection with miR200c resulted in a 2.4 ± 0.3 fold (figure 4b) reduction in the levels of FAP-1 when compared to fibroblasts transfected with miR141 or scrambled miRNA. Further cotransfection with *mir*Vana® miRNA inhibitor for miR200c resulted in blocking the function of miR200c in the miR200c transfected fibroblast leading to an increase in the expression of FAP-1 similar to that observed in the untransfected or scrambled miRNA transfected fibroblast (0.8±0.4 vs 2.3±0.2, p<0.05).

### miR200c overexpression promotes fibrosis by modulating growth factor signaling through cSRC activation

**Figure 5:**
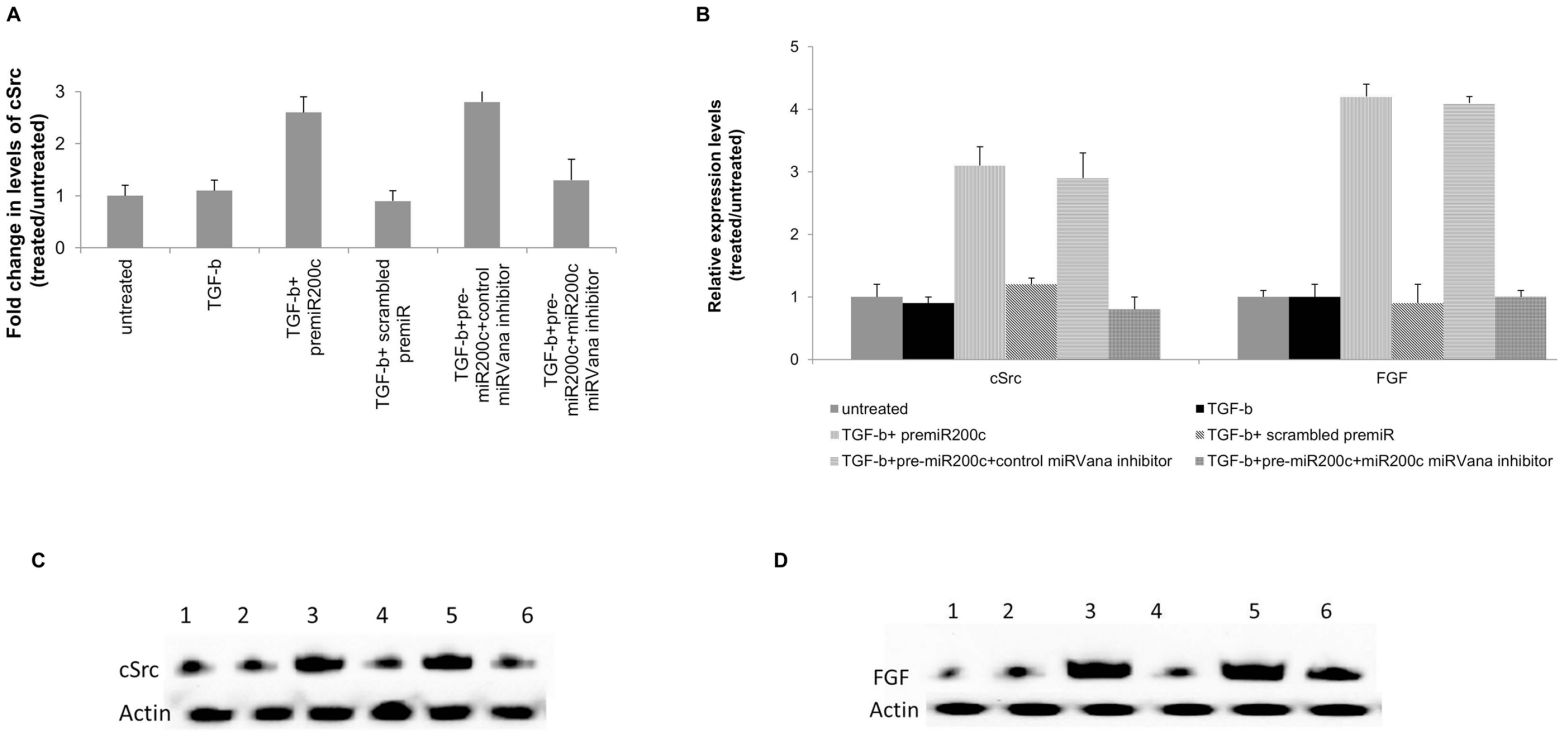
miR200c through modulation of growth factor signaling through cSrc activation promotes fibrosis. Human liver fibroblasts transfected with miR200c or scrambled miRNA were stimulated with TGF-β and analyzed for the expression of: a) cSrc at the message level by quantitative RT-PCR and b) relative expression levels of cSrc and FGF at protein level, c) representative western blot analysis of cSrc, and d) representative western blot analysis of FGF. miR200c overexpression results in a significant increase in expression of cSrc at the message level (2.6 fold) and cSrc and FGF at the protein level (3.1 and 4.2 fold respectively). Bars represent the mean relative expression levels from 3 different experiments performed with 3 different fibroblasts.

In silico combinatorial analysis of the miRNA and mRNA array data demonstrated that miR200c targets src signaling by directly targeting FAP1, a critical event in the development of fibrosis (figure 2). Murielle Glondu-Lassis et al have shown that PTPN13 or FAP-1, a phosphatase can inhibit Src through direct dephosphorylation in intact cells [23]. In order to determine the biological role of miR200c in the development of fibrosis we transfected primary human liver fibroblasts with pre-miR200c and stimulated them with TGF-β for 24 hrs and analyzed the levels of cSrc at the message level by quantitative RT-PCR and activated cSrc and FGF at protein level by western blot analysis. A 2.6 fold increase (p<0.05) in the levels of cSrc expression at the message level was observed in the fibroblasts transfected with miR200c (figure 5a). Blocking of the miR200c by cotransfection with *mir*Vana® miRNA inhibitor for miR200c resulted in amelioration of the increased expression of cSrc observed in the miR200c transfected fibroblasts to the levels observed in the untransfected or scrambled miRNA transfected controls (1.3±0.4 vs 2.8±0.4, p<0.05). Western blot analysis of the levels of cSrc levels demonstrated a 3.1 fold increase (p<0.05) in the fibroblasts transfected with miR200c when compared to fibroblasts transfected with scrambled miRNA (figure 5b). Similarly, western blot analysis also demonstrated a 4.2 fold increase (p<0.05) in the levels of FGF in the miR200c transfected fibroblasts when compared to scrambled miRNA transfected fibroblasts (figure 5b). These results demonstrate that miR200c targets the cSrc regulator FAP1 leading to dysregulated signaling through the growth factors leading to increased production of pro-fibrogenic growth factors.

### miR200c promotes fibrosis through dysregulation of cSrc signaling cascade

**Figure 6:**
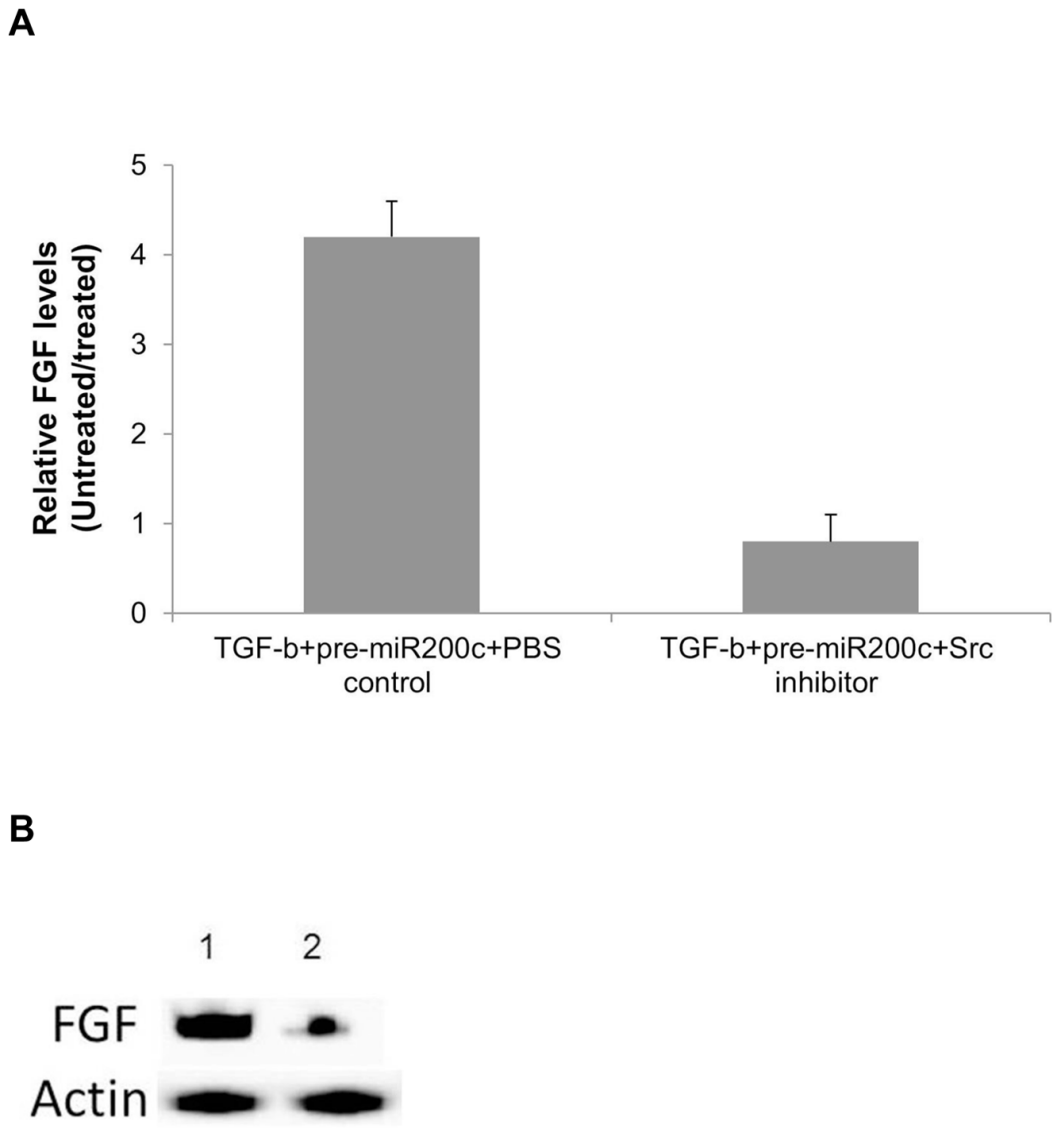
Src Inhibition reverses the action of miR200c. Human liver fibroblasts transfected with miR200c were stimulated with TGF-β in the presence of Src inhibitor (10 µM) or PBS control. a) Expression levels of FGF were analyzed at the protein level by western blot analysis. Bars represent the mean relative expression observed in 3 different set of experiments performed with 3 different fibroblasts. b) representative western blot analysis of FGF levels. Treatment with Src inhibitor decreased the levels of FGF expression in the miR200c transfected cells to the levels observed in the untransfected fibroblasts.

In order to determine the signaling cascade through which miR200c may promote fibrosis, we transfected human liver fibroblasts with pre-miR200c and stimulated them with TGF-β for 24 hrs. The cells were treated with Src inhibitor-1 (10 µM) or controls during stimulation with TGF-β. Analysis of the FGF levels in the cSrc inhibitor treated cells showed no significant increase (4.2± 0.3 vs 0.8±0.3, p>0.05) in the FGF levels in the miR200c transfected fibroblasts when compared to controls (figure 6). These results also support the conclusion that miR200c through FAP-1 modulates the cSrc signaling cascade which can promote development of fibrosis.

### miR200c and its gene targets that promote development of fibrosis is modulated in chronic HCV

**Figure 7:**
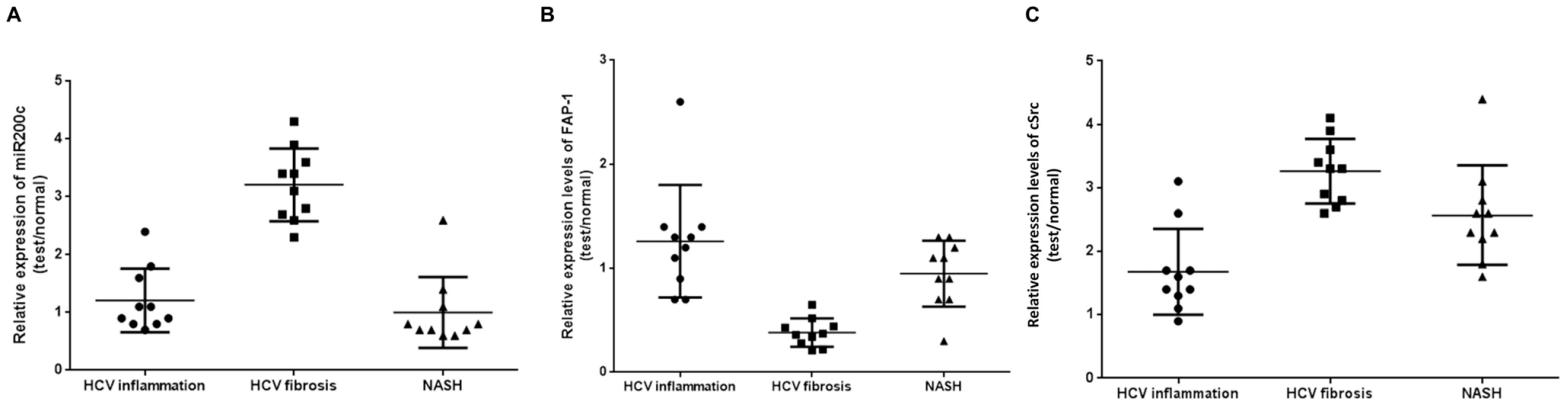
Modulation of growth factor signaling cascade in chronic HCV infection. Liver biopsies were obtained from 10 HCV infected patients with inflammation but no fibrosis, 10 HCV infected patients with grade III/IV fibrosis and 10 patients with NASH. Expression levels of: a) mIR200c; b) FAP-1 and c) cSrc were analyzed by quantitative RT-PCR using pre-developed Taqman miRNA assays. The small RNA U6b was used as an endogenous control and relative miRNA quantity was calculated by the ΔΔCt method. The horizontal line represents the average and standard deviation of fold expression levels in the groups.

Analysis of miRNA and mRNA arrays suggested that HCV infection can modulate the growth factor signaling through the modulation of miR200c. In order to validate the microarray data, we analyzed the levels of miR 200c, FAP1 and cSrc that modulate the growth factor signaling cascades by quantitative RT-PCR. The levels of miR200c in the liver biopsies obtained from 10 HCV infected livers with inflammation but no fibrosis, 10 HCV infected livers with grade III/IV fibrosis and 10 liver biopsies from patients with non-alcoholic steatohepatitis (NASH) were analyzed by quantitative RT-PCR using TaqMan^®^ MicroRNA Assay. RT-PCR analysis of the miRNA levels in the chronic HCV infected liver (figure 7a) demonstrated a 3.3 ±0.9 fold increase in the levels of miR 200c in the chronic HCV infected liver whereas the levels in the HCV infected livers with inflammation but no fibrosis (1.1 fold) and NASH (0.9 fold) showed no significant increase in the levels of miR200c when compared to the normal livers (figure 7a). The levels of FAP-1 (figure 7b) the immediate target of miR200c was down regulated by 2.6 fold in the chronic HCV infected liver with fibrosis but was not affected in HCV livers with no fibrosis (1.3 fold) or in NASH (1.0 fold). However, the levels of cSrc (figure 7c) were significantly elevated in chronic HCV (3.2 ± 0.4) and NASH (2.4 ± 0.7) but not in HCV infected liver with no fibrosis (1.7 ± 1.1).These results demonstrate that HCV infection leads to a significant modulation of the miRNA profile that promotes development of fibrosis following chronic HCV infection. Similarly, analysis of the FAP-1 levels demonstrated a 2.1 fold reduction in the HCV infected livers with fibrosis, 1.6 fold reductions in the NASH but showed no significant reduction in HCV infected liver with inflammation but no fibrosis when compared to control normal livers. These observations that the increase in miR200c and decrease in the FAP-1 levels in the HCV infected liver but not in the NASH livers suggest that chronic HCV infection through the modulation of miR200c promotes the development of fibrosis.

## Discussion

Chronic HCV infection is one of the major indications for hepatitis, cirrhosis and hepatocellular carcinoma worldwide. Chronic HCV infection results in increased production of profibrotic cytokines and growth factors that result in fibrosis and cirrhosis of the liver leading to loss of liver functions. One potential mechanism by which HCV can evade the immune responses and induces fibrosis is through the modulation of miRNA levels that play a critical role in the gene expression. In this study, we demonstrate that chronic HCV infection results in the modulation of 45 miRNAs that alter growth factor signaling pathway, cell cycle regulation, epithelial mesenchymal transition and chemokines and cell adhesion that could promote fibrosis and immune evasion leading to loss of liver function. We also demonstrate that HCV infection, by increasing the levels of miR-200c, down modulates the expression of FAP1 or PTPN13, a regulator of c-Src activation. Decreased expression of FAP-1 results in increased cSrc activation following growth factor signaling leading to increased production of pro-fibrogenic growth factors and components of extracellular matrix that promotes the development of fibrosis.

Analysis of the miRNA profile showed a >2 fold increase in the expression of 22 miRNAs and >2 reduction in the expression of 23 miRNAs. Functional grouping of the miRNAs based on the in silico target prediction and network building identified activation of phospholipase C pathway, cellular response to endogenous stimuli (growth factors and cytokine), and cellular proliferation as the major biological processes impacted by the modulated miRNAs. Similar to our studies, a distinct miRNA profile has been associated with HCV infection [24]. By comparing liver biopsies from HCV+ and HCV- LTx the authors demonstrated a significant increase in the expression of miR16 and down regulation of miR122 [24]. Similarly, an increase in the expression of miR-122, miR-100, miR-10a, miR198 and miR-145 has also been reported in HCV induced HCC tissues when compared to adjacent non-tumor tissue [25]. Shackle et al has also reported that in HCV-associated cirrhosis the genes up regulated were predominantly associated with a Th1 immune response, fibrosis, cellular proliferation, and apoptosis [26]. A study using a whole genome array has also reported that genes of the immune system, extracellular matrix turnover, anti-apoptotic pathway, interferon pathway and virus host interactions are differentially expressed in the liver biopsies from chronic HCV when compared to normal livers [27]. However, the mechanism by which HCV modulates the differential gene expression has not been explored. In silico analysis of the miRNA by complementary pairing analysis suggest that each miRNA could potentially target multiple mRNAs and impact their expression profile. Partek analysis identified 289 potential interactions that can modulate several biological processes including metabolic processes, cell proliferation and response to stimulus. In order to further validate these studies, we analyzed the levels of TGF-β expression in sera of 20 chronic HCV patients and compared them to normal subjects. Significant increases (>8 fold) in the levels of TGF-β in chronic HCV infected patients when compared to normal controls (figure 3). Since increased TGF-β levels can increase the production of extracellular matrix proteins and their receptors and inhibit the synthesis of matrix-degradative proteolytic enzymes we postulate that HCV infection through the modulation of TGF-β signaling can promote immune evasion, viral persistence as well as induction of fibrosis leading to end stage liver disease.

Fibrosis is the critical event in liver cirrhosis leading to loss of liver functions. We defined that chronic HCV infection results in the modulation of miRNA 10b and 200c which can regulate the expression of transcription factors, Homeobox D10 and ZFHX1B [28]. It has been reported that the interactions of yEF1/ZEB1 and the miRNA-200 family members miR-141 and miR-200c are part of a transcriptional feed forward loop that stabilizes epithelial mesenchymal transition (EMT) and promotes cancer invasion [29]. Microdissected metaplastic breast carcinoma has been shown to have a specific loss of miR-200 which correlates with increased expression of vimentin and decreased E-cadherin in the mesenchymal component [30], [31]. Therefore, significant modulation of miRNA 141, 200 and 10c seen in our analysis can result in the modulation of the expression of transcription factors that promote the induction of EMT.

Binding of TGF-β to its cognate receptor on the target cells results in recruitment of Smad proteins that translocates to the nucleus and with coactivators results in the increased profibrotic and anti-apoptotic genes that contribute to the development of fibrosis [32], [33] Src kinases are important mediators of pro-fibrotic signaling pathways [34], [35] and can modulate the activity of TGF-β signaling by phosphorylating and activating TGF-β type II receptor and the downstream target c-Abl [36]. A specific inhibitor of Src kinase (SU6656) can effectively abrogate fibrosis in an experimental model of fibrosis [37]. FAP1 or PTPN13, a nonreceptor type protein tyrosine phosphatase with the highest molecular weight, 270 kDa, contains multiple interactive domains has been shown to directly interact with Src and modulates its downstream effects [38]. It has been shown that miR200c can directly modulate the expression of FAP-1 [39]. In our study, miRNA expression profiling of the chronic HCV livers demonstrated a 2.9 fold increase (Table 2) in the expression of miR200c which was then validated by quantitative RT-PCR in a set of 10 chronic HCV samples (figure 7a). We also identified that the miRNA 200c target FAP1 is significantly down regulated in chronic HCV (figure 7b). A significant (3-6 fold) increase in the expression of TGF-β , FGF, VEGF, and PDGF in chronic HCV liver was also noted along with significant increases (2–8 fold) in the expression of ECM proteins collagen, fibronectin and vimentin (Table S1). Using *in vitro* methods, we demonstrated that transfection of human fibroblasts with miR200c results in a 2.1 fold decrease in expression of FAP-1 (figure 4b) with a corresponding 2.2 fold increase in the levels of activated cSrc (figure 4C) leading to a 3.4 fold increase in expression of FGF (figure 5) and 4.2 fold increase in expression of FGF (figure 5). Blocking of Src signaling through an inhibitor (Src inhibitor-1) following transfection with miR200c of liver fibroblasts did not result in the increased expression of FGF or Col-V (figure 6). Based on these, we conclude that the increased levels of miR200c in the chronic HCV infected liver results in the decreased expression of FAP-1, the negative regulator of Src kinase leading to dysregulated TGF-β signaling that promotes development of fibrosis. A putative pathway through which modulated miRNAs in chronic HCV infected liver that promotes fibrosis is presented in figure 2.

In conclusion, using miRNA expression profiling and whole genome array analysis we demonstrate that chronic HCV infection results in the modulation of several miRNAs. We also present evidence that HCV infection increases the levels of a specific miRNA, miR200c which regulates the expression of FAP-1, a negative regulator and impacts the growth factor signaling pathway by modulating the Src kinase signaling cascade plays an important role in development of fibrosis.

## Supporting Information

Table S1 **Transcription profile of the chronic HCV liver.** Total RNA was isolated from the liver biopsies and mRNA expression analysis was done using the Illumina HumanHT-12 v3 Expression BeadChips array that contains (48000) probe sets. Gene expression levels in the samples were normalized based on the 16s ribosomal RNAs and housekeeping gene expression. PARTEK analysis software was used to analyze the differential expression profile of the genes. Changes in gene expression level of 2-fold or more in the HCV samples compared with the normal controls with p value <0.05 (based on ANOVA analysis) were considered to be significant. Chronic HCV patients had greater than two fold increases in the expression of 785 genes and greater than 2 fold reduction in the expression of 533 genes.
(PDF)
