# Supplementary material for: Hepatitis C Virus Induced miR200c Down Modulates FAP-1, a Negative Regulator of Src Signaling and Promotes Hepatic Fibrosis
Source: PLoS One. 2013 Aug 12;8(8):e70744. doi: 10.1371/journal.pone.0070744 (PMC3741284; doi:10.1371/journal.pone.0070744)
Supplement: Table S1 — Transcription profile of the chronic HCV liver. Total RNA was isolated from the liver biopsies and mRNA expression analysis was done using the Illumina HumanHT-12 v3 Expression BeadChips array that contains (48000) probe sets. Gene expression levels in the samples were normalized based on the 16s ribosomal RNAs and housekeeping gene expression. PARTEK analysis software was used to analyze the differential expression profile of the genes. Changes in gene expression level of 2-fold or more in the HCV samples compared with the normal controls with p value <0.05 (based on ANOVA analysis) were considered to be significant. Chronic HCV patients had greater than two fold increases in the expression of 785 genes and greater than 2 fold reduction in the expression of 533 genes. (PDF) [file pone.0070744.s001.pdf]

Probe ID	SYMBOL	CHROMOSOME	DEFINITION	SYNONYMS	p-value(HCV vs. Normal)	FoldChange(HCV vs. Normal)
ILMN_1751607	FDFT1	8	Homo sapiens farnesyl-diphosphate farnesyltransferase 1 (FDFT1), mRNA.	SS; DGPT; SQS; ERG9	0.00850675	27.0379
ILMN_1664464	DNAJC3	13	Homo sapiens DnaJ (Hsp40) homolog, subfamily C, member 3 (DNAJC3), mRNA.	PRKRI; HP58; P58IPK; P5	0.0445901	24.4452
ILMN_1680274	MT1H	16	Homo sapiens metallothionein 1H (MT1H), mRNA.	MGC70702; MT1	0.0435413	22.5672
ILMN_1699214	FAM96A	15	Homo sapiens family with sequence similarity 96, member A (FAM96A), transcript variant 2, mRNA.	FLJ22875	0.0330944	18.8945
ILMN_1790529	SNCA	4	Homo sapiens synuclein, alpha (non A4 component of amyloid precursor) (SNCA), transcript variant NACP112, mRNA.	PD1; NACP; PARK4; MGC	0.0302102	17.8078
ILMN_1739508	IHPK3	6	Homo sapiens inositol hexaphosphate kinase 3 (IHPK3), mRNA.	IP6K3; MGC102928; INSF	0.0294882	17.5373
ILMN_1720048	LHFP	13	Homo sapiens lipoma HMGIC fusion partner (LHFP), mRNA.		0.0174947	17.1514
ILMN_1804601	PCTP	17	Homo sapiens phosphatidylcholine transfer protein (PCTP), mRNA.	STARD2	0.0476138	16.537
ILMN_2350634	PTGDR	14	Homo sapiens prostaglandin D2 receptor (DP) (PTGDR), mRNA.	MGC49004; ASRT1; DP;	0.0167806	16.1553
ILMN_1678842	SLC35D1	1	Homo sapiens solute carrier family 35 (UDP-glucuronic acid/UDP-N-acetylgalactosamine dual transporter), member D1 (SLC35D1), mRNA.	KIAA0260; MGC138236;	0.000906238	14.4414

ILMN_1687501	FILIP1L	3	Homo sapiens filamin A interacting protein 1-like (FILIP1L), transcript variant 2, mRNA.	DOC-1; GIP90; DOC1	0.0124355	13.5582
ILMN_1658494	ACN9	7	Homo sapiens ACN9 homolog (S. cerevisiae) (ACN9), mRNA.	DC11	0.00795725	13.1636
ILMN_1651958	CSAG3A	X	Homo sapiens CSAG family, member 3A (CSAG3A), mRNA.	MGC17065	0.00230244	11.8389
ILMN_2071809	MATK	19	Homo sapiens megakaryocyte-associated tyrosine kinase (MATK), transcript variant 3, mRNA.	DKFZp434N1212; HHYLTI	0.000258862	11.8375
ILMN_1653579	HAO2	1	Homo sapiens hydroxyacid oxidase 2 (long chain) (HAO2), transcript variant 2, mRNA.	HAOX2; GIG16	0.0350986	11.167
ILMN_1772964	ATN1	12	Homo sapiens atrophin 1 (ATN1), transcript variant 1, mRNA.	DRPLA; NOD; B37; D12S	0.0150017	10.7226
ILMN_1779875	CA1	8	Homo sapiens carbonic anhydrase I (CA1), mRNA.	Car1	0.034077	10.5553
ILMN_2098126	GPNMB	7	Homo sapiens glycoprotein (transmembrane) nmb (GPNMB), transcript variant 1, mRNA.	NMB; HGFIN	0.0439817	10.5233
ILMN_1743199	ST8SIA4	5	Homo sapiens ST8 alpha-N-acetyl-neuraminide alpha-2,8-sialyltransferase 4 (ST8SIA4), transcript variant 1, mRNA.	PST1; MGC61459; SIAT8	0.00200071	10.402
ILMN_1752755	DCN	12	Homo sapiens decorin (DCN), transcript variant C, mRNA.	PGII; DSPG2; CSCD; PGS	0.00529227	10.1759
ILMN_1706505	DNAJC12	10	Homo sapiens DnaJ (Hsp40) homolog, subfamily C, member 12 (DNAJC12), transcript variant 2, mRNA.	RP11-57G10.2; JDP1	0.00623458	9.85081
ILMN_2054297			AGENCOURT_10520654 NIH_MGC_128 Homo sapiens cDNA clone IMAGE:6702588 5, mRNA sequence		0.0202577	9.80606

ILMN_2104356	LOC613037	16	Homo sapiens nuclear pore complex interacting protein pseudogene (LOC613037) on chromosome 16.		0.010573	9.40124
ILMN_1679262	CLEC1B	12	Homo sapiens C-type lectin domain family 1, member B (CLEC1B), transcript variant 1, mRNA.	1810061I13Rik; CLEC2B;	0.00101639	9.09621
ILMN_1790689	THY1	11	Homo sapiens Thy-1 cell surface antigen (THY1), mRNA.	CD90; FLJ33325	0.0255171	8.60203
ILMN_2179083	MYO1G	7	Homo sapiens myosin IG (MYO1G), mRNA.	HA-2; MGC142104	0.000408193	8.22701
ILMN_1772910	TMSB10	2	Homo sapiens thymosin beta 10 (TMSB10), mRNA.	MIG12; TB10	0.00766838	8.16496
ILMN_1785272	ACACA	17	Homo sapiens acetyl-Coenzyme A carboxylase alpha (ACACA), transcript variant 2, mRNA.	ACC1; ACAC; ACCA; ACC	0.00539356	8.16197
ILMN_1653466	KCTD14	11	Homo sapiens potassium channel tetramerisation domain containing 14 (KCTD14), mRNA.	MGC2376	0.0147468	8.15493
ILMN_1762899	SYK	9	Homo sapiens spleen tyrosine kinase (SYK), mRNA.		0.0012691	8.01957
ILMN_1656920	AQP12A	2	Homo sapiens aquaporin 12A (AQP12A), mRNA.	AQPX2; AQP12	0.000819727	7.8523
ILMN_1810289	CCND2	12	Homo sapiens cyclin D2 (CCND2), mRNA.	KIAK0002; MGC102758	0.000631499	7.69727
ILMN_2328666	FKSG24	19	Homo sapiens hypothetical protein MGC12972 (FKSG24), mRNA.	MGC110861; MGC12972	0.0219381	7.68178
ILMN_1671703	KLB	4	Homo sapiens klotho beta (KLB), mRNA.	BKL; MGC142213	0.00117047	7.67315
ILMN_1723035	TMEM56	1	Homo sapiens transmembrane protein 56 (TMEM56), mRNA.	MGC102912; FLJ31842	0.0111053	7.61496
ILMN_1672536	NRG4	15	Homo sapiens neuregulin 4 (NRG4), mRNA.	HRG4; DKFZp779N0541;	0.00951408	7.56529

ILMN_2127842	IGLL1	22	Homo sapiens immunoglobulin lambda-like polypeptide 1 (IGLL1), transcript variant 1, mRNA.	IGO; IGVPB; IGLL; CD179	0.0291169	7.53433
ILMN_2230025	DEFA3	8	Homo sapiens defensin, alpha 3, neutrophil-specific (DEFA3), mRNA.	HNP3; HNP-3; DEF3; HP-	2.65E-05	7.45335
ILMN_1673566			Homo sapiens, clone IMAGE:5528576, mRNA		0.0209274	7.44703
ILMN_1667796	BRCA1	17	Homo sapiens breast cancer 1, early onset (BRCA1), transcript variant BRCA1-delta14-17, mRNA.	RNF53; BRCAI; PSCP; BR	0.0221319	7.44411
ILMN_1736178	C5orf29	5	Homo sapiens chromosome 5 open reading frame 29 (C5orf29), mRNA.	MGC70478; FLJ33641	0.00510722	7.39146
ILMN_1734653	TLR7	X	Homo sapiens toll-like receptor 7 (TLR7), mRNA.		0.0194564	7.2746
ILMN_1653028	FGA	4	Homo sapiens fibrinogen alpha chain (FGA), transcript variant alpha-E, mRNA.	MGC119425; MGC119423	0.0019621	7.14518
ILMN_1789196	TNFRSF6B	20	Homo sapiens tumor necrosis factor receptor superfamily, member 6b, decoy (TNFRSF6B), transcript variant M68C, mRNA.	DJ583P15.1.1; M68; TR6	0.00285444	7.13857
ILMN_1778668	KGFLP1	9	Homo sapiens keratinocyte growth factor-like protein 1 (KGFLP1) on chromosome 9.	MGC125747; MGC126891	0.00820784	7.13203
ILMN_1655595	HAO2	1	Homo sapiens hydroxyacid oxidase 2 (long chain) (HAO2), transcript variant 1, mRNA.	GIG16; HAOX2	0.0102935	7.13158
ILMN_2400935	FLJ45139	21	Homo sapiens FLJ45139 protein (FLJ45139), mRNA.		0.0113534	7.10884
ILMN_2393765	PIGP	21	Homo sapiens phosphatidylinositol glycan anchor biosynthesis, class P (PIGP), transcript variant 2, mRNA.	DCRC-S; DSCR5; DCRC;	0.0190876	7.10592

ILMN_1780582	DBNDD2	20	Homo sapiens dysbindin (dystrobrevin binding protein 1) domain containing 2 (DBNDD2), transcript variant 3, mRNA.	HSMNP1; CK1BP; C20orf3	0.0241655	6.88431
ILMN_1688642	FKBP5	6	Homo sapiens FK506 binding protein 5 (FKBP5), mRNA.	FKBP54; P54; PPIase; Ptg	0.0178307	6.84879
ILMN_1775501	ALDH4A1	1	Homo sapiens aldehyde dehydrogenase 4 family, member A1 (ALDH4A1), nuclear gene encoding mitochondrial protein, transcript variant P5CDhL, mRNA.	P5CDh; P5CD; ALDH4; P5	0.00581907	6.74479
ILMN_1689146	COL4A1	13	Homo sapiens collagen, type IV, alpha 1 (COL4A1), mRNA.	arresten	0.019105	6.53057
ILMN_1726589	B3GALNT1	3	Homo sapiens beta-1,3-N-acetylgalactosaminyltransferase 1 (globoside blood group) (B3GALNT1), transcript variant 3, mRNA.	galT3; P1; B3GALT3; GLC	0.00111089	6.49869
ILMN_1735877	NDE1	16	Homo sapiens nudE nuclear distribution gene E homolog 1 (A. nidulans) (NDE1), mRNA.	FLJ20101; HOM-TES-87;	0.0099916	6.38602
ILMN_1670379	CMTM3	16	Homo sapiens CKLF-like MARVEL transmembrane domain containing 3 (CMTM3), transcript variant 5, mRNA.	BNAS2; MGC51956; CKLF	0.000473306	6.36486
ILMN_2067656	AFMID	17	Homo sapiens arylformamidase (AFMID), mRNA.	KF; DKFZp686F03259	0.00123571	6.34492
ILMN_1761788	GPR56	16	Homo sapiens G protein-coupled receptor 56 (GPR56), transcript variant 2, mRNA.	BFPP; DKFZp781L1398; T	0.0193817	6.31374
ILMN_2184184	FLJ21438	19	Homo sapiens hypothetical protein FLJ21438 (FLJ21438), mRNA.	DKFZp667E013; FLJ0008	0.00175813	6.29803

ILMN_1656057	SLC31A2	9	Homo sapiens solute carrier family 31 (copper transporters), member 2 (SLC31A2), mRNA.	hCTR2; CTR2; COPT2	0.00266231	6.21525
ILMN_1772612	SLCO3A1	15	Homo sapiens solute carrier organic anion transporter family, member 3A1 (SLCO3A1), mRNA.	FLJ40478; SLC21A11; OA	0.00067693	6.14936
ILMN_1676449	RFX5	1	Homo sapiens regulatory factor X, 5 (influences HLA class II expression) (RFX5), transcript variant 2, mRNA.		0.00188831	6.13656
ILMN_1768940	YWHAZ	8	Homo sapiens tyrosine 3-monooxygenase/tryptophan 5-monooxygenase activation protein, zeta polypeptide (YWHAZ), transcript variant 1, mRNA.	KCIP-1; MGC126532; MG	0.023983	5.92257
ILMN_1782538	F3	1	Homo sapiens coagulation factor III (thromboplastin, tissue factor) (F3), mRNA.	TF; TFA; CD142	0.00316895	5.87898
ILMN_1654324			AGENCOURT_10404673 NIH_MGC_82 Homo sapiens cDNA clone IMAGE:6615135 5, mRNA sequence		0.0043923	5.84416
ILMN_1738742	DMRTA1	9	Homo sapiens DMRT-like family A1 (DMRTA1), mRNA.	MGC163307; DMO; MGC1	0.00739488	5.81201
ILMN_2352131	LPA	6	Homo sapiens lipoprotein, Lp(a) (LPA), mRNA.	LP; APOA; AK38	1.53E-05	5.79939
ILMN_1741688	C13orf15	13	Homo sapiens chromosome 13 open reading frame 15 (C13orf15), mRNA.	RGC-32; KIAA0564; bA15	0.000324392	5.76927
ILMN_1773079	MS4A3	11	Homo sapiens membrane-spanning 4-domains, subfamily A, member 3 (hematopoietic cell-specific) (MS4A3), transcript variant 1, mRNA.	CD20L; HTM4	0.00166452	5.73205

ILMN_1741356	MARCKS	6	Homo sapiens myristoylated alanine-rich protein kinase C substrate (MARCKS), mRNA.	MACS; 80K-L; PRKCSL; F	0.00529303	5.67998
ILMN_1723684	BEXL1		PREDICTED: Homo sapiens brain expressed X-linked-like 1 (BEXL1), mRNA.		0.00131132	5.63846
ILMN_2143795	IL15	4	Homo sapiens interleukin 15 (IL15), transcript variant 3, mRNA.	MGC9721; IL-15	0.033597	5.60579
ILMN_1806667	SUSD3	9	Homo sapiens sushi domain containing 3 (SUSD3), mRNA.	MGC26847	0.0365778	5.53998
ILMN_1700541	FAM129B	9	Homo sapiens family with sequence similarity 129, member B (FAM129B), transcript variant 1, mRNA.	OC58; MEG-3; FLJ13518;	0.00294163	5.52412
ILMN_1790555	COL6A2	21	Homo sapiens collagen, type VI, alpha 2 (COL6A2), transcript variant 2C2, mRNA.	PP3610; DKFZp586E1322	0.0123958	5.51744
ILMN_1662358	STEAP1	7	Homo sapiens six transmembrane epithelial antigen of the prostate 1 (STEAP1), mRNA.	MGC19484; PRSS24; STE	0.0476296	5.49019
ILMN_1773006	ARHGEF2	1	Homo sapiens rho/rac guanine nucleotide exchange factor (GEF) 2 (ARHGEF2), mRNA.	P40; GEF; LFP40; DKFZp	0.00575156	5.45486
ILMN_1746565	MOGAT2	11	Homo sapiens monoacylglycerol O-acyltransferase 2 (MOGAT2), mRNA.	DGAT2L5; MGC119183; F	0.0328181	5.43587
ILMN_1805737	COL1A2	7	Homo sapiens collagen, type I, alpha 2 (COL1A2), mRNA.	OI4	0.0189567	5.41274
ILMN_1800739	FAM43A	3	Homo sapiens family with sequence similarity 43, member A (FAM43A), mRNA.	FLJ90022	0.0476368	5.39152
ILMN_1660462	NOS1AP	1	Homo sapiens nitric oxide synthase 1 (neuronal) adaptor protein (NOS1AP), mRNA.	MGC138500; CAPON	0.0351977	5.37559

ILMN_2246956	TNFSF10	3	Homo sapiens tumor necrosis factor (ligand) superfamily, member 10 (TNFSF10), mRNA.	TL2; Apo-2L; CD253; APO	0.00810669	5.3402
ILMN_1782305			PREDICTED: Homo sapiens hypothetical LOC401062 (LOC401062), mRNA		0.0122334	5.27908
ILMN_2112638	RELN	7	Homo sapiens reelin (RELN), transcript variant 1, mRNA.	RL	0.0162312	5.27509
ILMN_1815673	GSN	9	Homo sapiens gelsolin (amyloidosis, Finnish type) (GSN), transcript variant 2, mRNA.	DKFZp313L0718	0.00436993	5.24269
ILMN_1757604	AGXT2	5	Homo sapiens alanine-glyoxylate aminotransferase 2 (AGXT2), nuclear gene encoding mitochondrial protein, mRNA.	AGT2	0.00618568	5.21584
ILMN_2374865	HBA2	16	Homo sapiens hemoglobin, alpha 2 (HBA2), mRNA.	HBA1	0.0346004	5.16617
ILMN_1686573	NLF2		PREDICTED: Homo sapiens nuclear localized factor 2 (NLF2), mRNA.		0.0277116	5.11733
ILMN_2329914	LOC23117	16	PREDICTED: Homo sapiens KIAA0220-like protein, transcript variant 16 (LOC23117), mRNA.		0.00813694	5.11594
ILMN_2316236	ISG20	15	Homo sapiens interferon stimulated exonuclease gene 20kDa (ISG20), mRNA.	CD25; HEM45	0.0120227	5.11335
ILMN_1756439	CLEC4G	19	Homo sapiens C-type lectin superfamily 4, member G (CLEC4G), mRNA.	LSECtin; LP2698; UNQ43	0.024647	5.08755
ILMN_2398159	LOC153561	5	Homo sapiens hypothetical protein LOC153561 (LOC153561), mRNA.		0.00933763	5.08398

ILMN_1716246	ALDH4A1	1	Homo sapiens aldehyde dehydrogenase 4 family, member A1 (ALDH4A1), nuclear gene encoding mitochondrial protein, transcript variant P5CDhS, mRNA.	P5CDh; P5CD; ALDH4; P5	0.00316614	5.04882
ILMN_1659075	MANEA	6	Homo sapiens mannosidase, endo-alpha (MANEA), mRNA.	FLJ12838; hEndo; DKFZp	0.0116576	5.02835
ILMN_1691860	ISLR	15	Homo sapiens immunoglobulin superfamily containing leucine-rich repeat (ISLR), transcript variant 1, mRNA.	HsT17563; MGC102816	0.00726262	5.02663
ILMN_1656501	SLC44A1	9	Homo sapiens solute carrier family 44, member 1 (SLC44A1), mRNA.	RP11-287A8.1; CHTL1; C	0.0110374	5.00315
ILMN_1687384	IDH2	15	Homo sapiens isocitrate dehydrogenase 2 (NADP+), mitochondrial (IDH2), nuclear gene encoding mitochondrial protein, mRNA.	ICD-M; IDHM; IDH; IDP;	0.0328017	4.99946
ILMN_1738116	FBLN2	3	Homo sapiens fibulin 2 (FBLN2), transcript variant 2, mRNA.		0.00363588	4.97081
ILMN_2058251			K-EST0224780 L17N670205n1 Homo sapiens cDNA clone L17N670205n1-28-B04 5, mRNA sequence		0.000670946	4.94067
ILMN_1706643	KBTBD9	2	PREDICTED: Homo sapiens kelch repeat and BTB (POZ) domain containing 9 (KBTBD9), mRNA.		0.00385789	4.86792
ILMN_1734276	NCOA7	6	Homo sapiens nuclear receptor coactivator 7 (NCOA7), mRNA.	FLJ45605; dJ187J11.3; E	0.0325988	4.86491
ILMN_1736567	MAB21L2		PREDICTED: Homo sapiens mab-21-like 2 (C. elegans) (MAB21L2), mRNA.		0.00681675	4.84719

ILMN_2170814	IFI16	1	Homo sapiens interferon, gamma-inducible protein 16 (IFI16), mRNA.	IFNGIP1; PYHIN2	0.019963	4.83154
ILMN_1738578	ARHGAP4	X	Homo sapiens Rho GTPase activating protein 4 (ARHGAP4), mRNA.	p115; C1; RGC1; KIAA01	0.00755451	4.82694
ILMN_1815057	GPD1	12	Homo sapiens glycerol-3-phosphate dehydrogenase 1 (soluble) (GPD1), mRNA.	FLJ26652	0.0017036	4.82055
ILMN_2062468	STAT4	2	Homo sapiens signal transducer and activator of transcription 4 (STAT4), mRNA.		0.0114465	4.70951
ILMN_1815500	MME	3	Homo sapiens membrane metallo-endopeptidase (MME), transcript variant 1, mRNA.	MGC126681; NEP; CD10;	0.0130727	4.70651
ILMN_1755383	SPIRE1	18	Homo sapiens spire homolog 1 (Drosophila) (SPIRE1), mRNA.	Spir-1; MGC150622; MG[illegible]	0.00213588	4.70613
ILMN_1797822	DDEF1	8	Homo sapiens development and differentiation enhancing factor 1 (DDEF1), mRNA.	ZG14P; AMAP1; PAG2; A[illegible]	0.00564541	4.66707
ILMN_1789733	FDFT1	8	Homo sapiens farnesyl-diphosphate farnesyltransferase 1 (FDFT1), mRNA.	SS; DGPT; SQS; ERG9	0.00726868	4.64913
ILMN_1691376	IFT57	3	Homo sapiens intraflagellar transport 57 homolog (Chlamydomonas) (IFT57), mRNA.	ESRRBL1; MHS4R2; FLJ1[illegible]	0.00722885	4.62884
ILMN_1791890	FBLN1	22	Homo sapiens fibulin 1 (FBLN1), transcript variant D, mRNA.	FBLN	0.0185486	4.62155
ILMN_1758895	SERPINE2	2	Homo sapiens serpin peptidase inhibitor, clade E (nexin, plasminogen activator inhibitor type 1), member 2 (SERPINE2), mRNA.	PI7; GDN; PNI; PN1	0.00116083	4.58947
ILMN_2413158	MCOLN2	1	Homo sapiens mucolipin 2 (MCOLN2), mRNA.	TRP-ML2; TRPML2; FLJ36	0.00179989	4.57795

ILMN_1711015	DNASE1L3	3	Homo sapiens deoxyribonuclease I-like 3 (DNASE1L3), mRNA.	LSD; DNAS1L3; DHP2	0.0319023	4.5756
ILMN_1674063	SHMT1	17	Homo sapiens serine hydroxymethyltransferase 1 (soluble) (SHMT1), transcript variant 1, mRNA.	MGC15229; MGC24556; [illegible]	0.0400634	4.56232
ILMN_1766499	PDGFD	11	Homo sapiens platelet derived growth factor D (PDGFD), transcript variant 1, mRNA.	MSTP036; IEGF; MGC268	0.00942	4.53746
ILMN_1671295	FOXA1	14	Homo sapiens forkhead box A1 (FOXA1), mRNA.	MGC33105; TCF3A; HNF3	0.0314431	4.5072
ILMN_1772218	CCL3	17	Homo sapiens chemokine (C-C motif) ligand 3 (CCL3), mRNA.	SCYA3; MIP-1-alpha; G0[illegible]	0.0156619	4.49619
ILMN_1776181	TGIF1	18	Homo sapiens TGFB-induced factor homeobox 1 (TGIF1), transcript variant 1, mRNA.	HPE4; MGC5066; MGC39[illegible]	0.00822225	4.49515
ILMN_1747593	C6	5	Homo sapiens complement component 6 (C6), mRNA.		0.00122675	4.48961
ILMN_1713636	GLT8D2	12	Homo sapiens glycosyltransferase 8 domain containing 2 (GLT8D2), mRNA.	FLJ31494	0.0384111	4.4651
ILMN_1658356	ST3GAL6	3	Homo sapiens ST3 beta-galactoside alpha-2,3-sialyltransferase 6 (ST3GAL6), mRNA.	ST3GALVI; SIAT10	0.00548795	4.42856
ILMN_1687301	EVC2	4	Homo sapiens Ellis van Creveld syndrome 2 (limbin) (EVC2), mRNA.	LBN	0.0162229	4.41056
ILMN_2379644	ALDH8A1	6	Homo sapiens aldehyde dehydrogenase 8 family, member A1 (ALDH8A1), transcript variant 1, mRNA.	DKFZp779D2315; MGC13	0.0166786	4.38994
ILMN_1758128	ILK	11	Homo sapiens integrin-linked kinase (ILK), transcript variant 1, mRNA.	P59; DKFZp686F1765	0.00198617	4.38539

ILMN_1689655	LOC728037	16	PREDICTED: Homo sapiens similar to Kinesin-like protein KIF22 (Kinesin-like DNA-binding protein) (Kinesin-like protein 4) (LOC728037), mRNA.		0.0105017	4.38379
ILMN_1771688	LAYN	11	Homo sapiens layilin (LAYN), mRNA.	FLJ30977; FLJ31092	0.000531632	4.3812
ILMN_1735014	NSDHL	X	Homo sapiens NAD(P) dependent steroid dehydrogenase-like (NSDHL), mRNA.	H105E3; XAP104	0.0070912	4.35773
ILMN_2309156	CHN2	7	Homo sapiens chimerin (chimaerin) 2 (CHN2), transcript variant 2, mRNA.	BCH; ARHGAP3; RHOGAP	0.0309475	4.35064
ILMN_1799467	PDPN	1	Homo sapiens podoplanin (PDPN), transcript variant 4, mRNA.	Gp38; OTS8; T1A; PA2.2(	0.00804747	4.31624
ILMN_1665526	C20orf103	20	Homo sapiens chromosome 20 open reading frame 103 (C20orf103), mRNA.		0.0298176	4.26244
ILMN_2390919	TMEM178	2	Homo sapiens transmembrane protein 178 (TMEM178), mRNA.	MGC33926	0.0180019	4.25635
ILMN_2087692	TRAF5	1	Homo sapiens TNF receptor-associated factor 5 (TRAF5), transcript variant 1, mRNA.	MGC:39780; RNF84	0.0358556	4.25339
ILMN_1695311	ADK	10	Homo sapiens adenosine kinase (ADK), transcript variant ADK-short, mRNA.	AK	0.0107724	4.21186
ILMN_2339955	SDCBP2	20	Homo sapiens syndecan binding protein (syntenin) 2 (SDCBP2), transcript variant 2, mRNA.	SITAC18; ST-2	0.0304891	4.19872
ILMN_1749070	MYH9	22	Homo sapiens myosin, heavy chain 9, non-muscle (MYH9), mRNA.	NMMHCA; DFNA17; EPST	0.0220959	4.18576

ILMN_2139970	TRPC1	3	Homo sapiens transient receptor potential cation channel, subfamily C, member 1 (TRPC1), mRNA.	HTRP-1; MGC133335; TR	0.00329213	4.17963
ILMN_1654072	PSCA	8	Homo sapiens prostate stem cell antigen (PSCA), mRNA.	PRO232	0.00078298	4.17902
ILMN_1796734	NPIP	16	Homo sapiens nuclear pore complex interacting protein (NPIP), mRNA.		0.0053826	4.16448
ILMN_1699521	COL3A1	2	Homo sapiens collagen, type III, alpha 1 (Ehlers-Danlos syndrome type IV, autosomal dominant) (COL3A1), mRNA.	FLJ34534; EDS4A	0.00603648	4.13894
ILMN_1699856	PCSK6	15	Homo sapiens proprotein convertase subtilisin/kexin type 6 (PCSK6), transcript variant 5, mRNA.	SPC4; PACE4	0.0061256	4.13347
ILMN_1651610	SAA1	11	Homo sapiens serum amyloid A1 (SAA1), transcript variant 1, mRNA.	MGC111216; SAA; PIG4;	0.0122741	4.12011
ILMN_1791328	ZNF83	19	Homo sapiens zinc finger protein 83 (ZNF83), mRNA.	MGC33853; FLJ90585; H	0.0402776	4.11856
ILMN_1699631	C9orf19	9	Homo sapiens chromosome 9 open reading frame 19 (C9orf19), mRNA.	GLIPR2; GAPR-1	0.0114268	4.11786
ILMN_2146761	CYBRD1	2	Homo sapiens cytochrome b reductase 1 (CYBRD1), mRNA.	FLJ23462; FRRS3; DCYTE	0.0094253	4.11201
ILMN_2084825			Homo sapiens cDNA FLJ13267 fis, clone OVARC1000964		0.0136562	4.10815
ILMN_2308849	WSB1	17	Homo sapiens WD repeat and SOCS box-containing 1 (WSB1), transcript variant 2, mRNA.	WSB-1; SWIP1	0.011657	4.09904
ILMN_1710434	UACA	15	Homo sapiens uveal autoantigen with coiled-coil domains and ankyrin repeats (UACA), transcript variant 1, mRNA.	MGC141967; FLJ10128; N	0.0498025	4.0806

ILMN_2163873	C6orf151	6	Homo sapiens chromosome 6 open reading frame 151 (C6orf151), mRNA.	dJ336K20B.1; FLJ32234;	0.029208	4.07842
ILMN_1810191	FGA	4	Homo sapiens fibrinogen alpha chain (FGA), transcript variant alpha-E, mRNA.	MGC119425; MGC119423	0.000975683	4.05568
ILMN_1696391	LOC654244		PREDICTED: Homo sapiens similar to mitochondrial carrier protein MGC4399 (LOC654244), mRNA.		0.0112632	4.05263
ILMN_2315979	ANK3	10	Homo sapiens ankyrin 3, node of Ranvier (ankyrin G) (ANK3), transcript variant 2, mRNA.	ANKYRIN-G; FLJ45464	0.0036603	4.05029
ILMN_1738075			UI-CF-FN0-aes-k-15-0-UI.s1 UI-CF-FN0 Homo sapiens cDNA clone UI-CF-FN0-aes-k-15-0-UI 3, mRNA sequence		0.00159714	4.04603
ILMN_2414268	C9orf75	9	Homo sapiens chromosome 9 open reading frame 75 (C9orf75), mRNA.	MGC131933; RP11-350O	0.024723	4.04519
ILMN_1712305	MGAT2	14	Homo sapiens mannosyl (alpha-1,6-)-glycoprotein beta-1,2-N-acetylglucosaminyltransferase (MGAT2), transcript variant 2, mRNA.	GNT-II; CDGS2; GLCNAC	0.0298026	4.02598
ILMN_2352097	HSD17B11	4	Homo sapiens hydroxysteroid (17-beta) dehydrogenase 11 (HSD17B11), mRNA.	DHRS8; PAN1B; RETSDR	0.0242367	4.01785
ILMN_2307861	LOC728358	8	Homo sapiens defensin, alpha 1 (LOC728358), mRNA.		0.00448797	4.01148
ILMN_1656560	ACACB	12	Homo sapiens acetyl-Coenzyme A carboxylase beta (ACACB), mRNA.	ACC2; ACCB; HACC275	0.0288032	4.00044
ILMN_1769615	POP5	12	Homo sapiens processing of precursor 5, ribonuclease P/MRP subunit (S. cerevisiae) (POP5), transcript variant 3, mRNA.	HSPC004; RPP20; RPP2	0.00116571	3.97865

ILMN_1709795	CD97	19	Homo sapiens CD97 molecule (CD97), transcript variant 2, mRNA.	TM7LN1	0.0405817	3.97476
ILMN_1687848	LMCD1	3	Homo sapiens LIM and cysteine-rich domains 1 (LMCD1), mRNA.		0.0402553	3.9747
ILMN_1774602	ARNT2	15	Homo sapiens aryl-hydrocarbon receptor nuclear translocator 2 (ARNT2), mRNA.	KIAA0307	0.00411538	3.96174
ILMN_1684554	TMEM149	19	Homo sapiens transmembrane protein 149 (TMEM149), mRNA.	U2AF1L4; FLJ22573	0.00219662	3.93599
ILMN_1657683	MRPL11	11	Homo sapiens mitochondrial ribosomal protein L11 (MRPL11), nuclear gene encoding mitochondrial protein, transcript variant 2, mRNA.	MGC111024; L11mt; CGI	0.0103161	3.91712
ILMN_1679809	CMTM3	16	Homo sapiens CKLF-like MARVEL transmembrane domain containing 3 (CMTM3), transcript variant 1, mRNA.	BNAS2; CKLFSF3; FLJ317	0.0245436	3.90744
ILMN_1740555	CRADD	12	Homo sapiens CASP2 and RIPK1 domain containing adaptor with death domain (CRADD), mRNA.	MGC9163; RAIDD	0.0132369	3.90334
ILMN_1805466	PDIA4	7	Homo sapiens protein disulfide isomerase family A, member 4 (PDIA4), mRNA.	ERP72; ERP70	0.025736	3.89792
ILMN_1767448	ATF3	1	Homo sapiens activating transcription factor 3 (ATF3), transcript variant 4, mRNA.		0.00245948	3.89361
ILMN_1659688	HEYL	1	Homo sapiens hairy/enhancer-of-split related with YRPW motif-like (HEYL), mRNA.	MGC12623; HRT3	0.0447363	3.87464
ILMN_2384122	CFHR1	1	Homo sapiens complement factor H-related 1 (CFHR1), mRNA.	CFHR1P; H36-2; HFL1; FI	0.028579	3.8697

ILMN_1730906	EFEMP1	2	Homo sapiens EGF-containing fibulin-like extracellular matrix protein 1 (EFEMP1), transcript variant 2, mRNA.	FBNL; MLVT; FLJ35535; F	0.0219255	3.85932
ILMN_1761733	TCEA2	20	Homo sapiens transcription elongation factor A (SII), 2 (TCEA2), transcript variant 2, mRNA.	TFIIS	0.00593502	3.85868
ILMN_1710495	LOC642197		PREDICTED: Homo sapiens similar to Protein FAM82B (LOC642197), mRNA.		0.00193731	3.839
ILMN_1691884	PMEPA1	20	Homo sapiens prostate transmembrane protein, androgen induced 1 (PMEPA1), transcript variant 2, mRNA.	STAG1; PMEPA1	0.0128005	3.83632
ILMN_2083066	ARHGDIB	12	Homo sapiens Rho GDP dissociation inhibitor (GDI) beta (ARHGDIB), mRNA.	Ly-GDI; RAP1GN1; GDIA2	0.00553702	3.82677
ILMN_1674985	MRPL20	1	Homo sapiens mitochondrial ribosomal protein L20 (MRPL20), nuclear gene encoding mitochondrial protein, mRNA.	MGC4779; L20mt; MGC74	0.0247404	3.81897
ILMN_1801205	IER5	1	Homo sapiens immediate early response 5 (IER5), mRNA.	SBBI48; MGC102760	0.0215162	3.81353
ILMN_1752899	COLEC11	2	Homo sapiens collectin sub-family member 11 (COLEC11), transcript variant 2, mRNA.	CL-K1-II; CL-K1-IIb; DKF	0.0221771	3.81196
ILMN_2237428	CD3E	11	Homo sapiens CD3e molecule, epsilon (CD3-TCR complex) (CD3E), mRNA.	T3E; TCRE	0.0409445	3.80321
ILMN_1727087	PLAT	8	Homo sapiens plasminogen activator, tissue (PLAT), transcript variant 1, mRNA.	T-PA; DKFZp686I03148;	0.0011757	3.80025
ILMN_2129161	SAMD9	7	Homo sapiens sterile alpha motif domain containing 9 (SAMD9), mRNA.	KIAA2004; C7orf5; OEF1	0.00353757	3.79484
ILMN_1717261	AOX1	2	Homo sapiens aldehyde oxidase 1 (AOX1), mRNA.	AO; AOH1	0.00199457	3.79373

ILMN_1705107	SLCO3A1	15	Homo sapiens solute carrier organic anion transporter family, member 3A1 (SLCO3A1), mRNA.	FLJ40478; SLC21A11; OA	0.0327773	3.79338
ILMN_1673639	TPD52L1	6	Homo sapiens tumor protein D52-like 1 (TPD52L1), transcript variant 3, mRNA.	D53; MGC8556; hD53; TI	0.0300491	3.78847
ILMN_1687533	CNDP1	18	Homo sapiens carnosine dipeptidase 1 (metallopeptidase M20 family) (CNDP1), mRNA.	CPGL2; CN1; MGC102737	0.0159411	3.75012
ILMN_1671509	SIGLEC10	19	Homo sapiens sialic acid binding Ig-like lectin 10 (SIGLEC10), mRNA.	SLG2; SIGLEC-10; PRO94	0.0206666	3.74479
ILMN_2197128	LOC649841		PREDICTED: Homo sapiens similar to protein immuno-reactive with anti-PTH polyclonal antibodies (LOC649841), mRNA.		0.00498723	3.72525
ILMN_2121408	SEMA4D	9	Homo sapiens sema domain, immunoglobulin domain (Ig), transmembrane domain (TM) and short cytoplasmic domain, (semaphorin) 4D (SEMA4D), mRNA.	SEMAJ; M-sema G; M-ser	0.00926763	3.71581
ILMN_2086077	CCDC58	3	Homo sapiens coiled-coil domain containing 58 (CCDC58), mRNA.	FLJ33273	0.0136285	3.70537
ILMN_1667966	LOC729485		PREDICTED: Homo sapiens hypothetical LOC729485 (LOC729485), mRNA.		0.00287281	3.70379
ILMN_1722713	TIMP2	17	Homo sapiens TIMP metallopeptidase inhibitor 2 (TIMP2), mRNA.	CSC-21K	0.00185118	3.70155
ILMN_2387385	ENPP2	8	Homo sapiens ectonucleotide pyrophosphatase/phosphodiesterase 2 (autotaxin) (ENPP2), transcript variant 2, mRNA.	NPP2; ATX; LysoPLD; ATX	0.0302269	3.69966

ILMN_1807042	PFKP	10	Homo sapiens phosphofructokinase, platelet (PFKP), mRNA.	PFKF; PFK-C; FLJ40226	0.00152368	3.69674
ILMN_2100437	XK	X	Homo sapiens X-linked Kx blood group (McLeod syndrome) (XK), mRNA.	XKR1; KX; X1k	0.0480992	3.67969
ILMN_2053829	SPRED1	15	Homo sapiens sprouty-related, EVH1 domain containing 1 (SPRED1), mRNA.	NFLS; FLJ33903	0.0212071	3.66996
ILMN_1811387	ELOVL6	4	Homo sapiens ELOVL family member 6, elongation of long chain fatty acids (FEN1/Elo2, SUR4/Elo3-like, yeast) (ELOVL6), mRNA.	MGC5487; FLJ23378; FAE	0.00218987	3.65464
ILMN_2262444	PTGER4	5	Homo sapiens prostaglandin E receptor 4 (subtype EP4) (PTGER4), mRNA.	EP4R; MGC126583; EP4	0.0195408	3.64827
ILMN_1796678	RTN1	14	Homo sapiens reticulon 1 (RTN1), transcript variant 1, mRNA.	NSP; MGC133250	0.015589	3.6286
ILMN_2341229	IDH1	2	Homo sapiens isocitrate dehydrogenase 1 (NADP+), soluble (IDH1), mRNA.	PICD; IDH; IDP	0.00336743	3.6187
ILMN_1702691	AP1G2	14	Homo sapiens adaptor-related protein complex 1, gamma 2 subunit (AP1G2), transcript variant 1, mRNA.	G2AD	0.00188199	3.61807
ILMN_1731206	WARS	14	Homo sapiens tryptophanyl-tRNA synthetase (WARS), transcript variant 1, mRNA.	IFI53; IFP53; GAMMA-2	0.0308821	3.6102
ILMN_1769782	GNA15	19	Homo sapiens guanine nucleotide binding protein (G protein), alpha 15 (Gq class) (GNA15), mRNA.	GNA16	0.0186003	3.60002
ILMN_1695299	KIAA0152	12	Homo sapiens KIAA0152 (KIAA0152), mRNA.		0.000728961	3.59614
ILMN_1724994	VIM	10	Homo sapiens vimentin (VIM), mRNA.	FLJ36605	0.010482	3.59441

ILMN_1746359	PLAU	10	Homo sapiens plasminogen activator, urokinase (PLAU), mRNA.	UPA; URK; u-PA; ATF	0.00282013	3.59121
ILMN_1704629	MUC6	11	Homo sapiens mucin 6, oligomeric mucus/gel-forming (MUC6), mRNA.	MUC6 mucin	0.0361896	3.57932
ILMN_2351466	MX2	21	Homo sapiens myxovirus (influenza virus) resistance 2 (mouse) (MX2), mRNA.	MXB	0.00769824	3.57134
ILMN_1795251	RALGDS	9	Homo sapiens ral guanine nucleotide dissociation stimulator (RALGDS), transcript variant 1, mRNA.	RGF; RalGEF; FLJ20922	0.00792582	3.5709
ILMN_1682775	SLC1A1	9	Homo sapiens solute carrier family 1 (neuronal/epithelial high affinity glutamate transporter, system Xag), member 1 (SLC1A1), mRNA.	EAAC1; EAAT3	0.0168829	3.57057
ILMN_1758497	TUBA1A	12	Homo sapiens tubulin, alpha 1a (TUBA1A), mRNA.	LIS3; FLJ25113; TUBA3;	0.043401	3.57045
ILMN_1780799	TNFRSF25	1	Homo sapiens tumor necrosis factor receptor superfamily, member 25 (TNFRSF25), transcript variant 10, mRNA.	WSL-LR; TNFRSF12; TR3	0.0279398	3.56781
ILMN_1691364	MT1A	16	Homo sapiens metallothionein 1A (MT1A), mRNA.	MTC; MT1; MGC32848; M	0.0405619	3.56167
ILMN_1687652	CEP68	2	Homo sapiens centrosomal protein 68kDa (CEP68), mRNA.	FLJ25920; KIAA0582	0.00339245	3.54584
ILMN_2193325	PPP1R13L	19	Homo sapiens protein phosphatase 1, regulatory (inhibitor) subunit 13 like (PPP1R13L), mRNA.	IASPP; RAI; iASPP gene;	0.00156644	3.54342
ILMN_2174805	SLC44A3	1	Homo sapiens solute carrier family 44, member 3 (SLC44A3), mRNA.	CTL3; MGC45474	0.0295907	3.54199
ILMN_1684349	DAB2	5	Homo sapiens disabled homolog 2, mitogen-responsive phosphoprotein (Drosophila) (DAB2), mRNA.	FLJ26626; DOC2; DOC-2	0.0278776	3.53155

ILMN_2336609	CD6	11	Homo sapiens CD6 molecule (CD6), mRNA.	TP120	0.0112768	3.53115
ILMN_2174341	BCL2	18	Homo sapiens B-cell CLL/lymphoma 2 (BCL2), nuclear gene encoding mitochondrial protein, transcript variant alpha, mRNA.	Bcl-2	0.0334303	3.52841
ILMN_1735930	FUT4	11	Homo sapiens fucosyltransferase 4 (alpha (1,3) fucosyltransferase, myeloid-specific) (FUT4), mRNA.	FCT3A; ELFT; FUC-TIV; C	0.00316266	3.52289
ILMN_2043079	MEG3	14	Homo sapiens maternally expressed 3 (MEG3) on chromosome 14. XR_001346-XR_001372	PRO2160; FP504; GTL2;	0.0242919	3.51509
ILMN_1702231	GJA1	6	Homo sapiens gap junction protein, alpha 1, 43kDa (GJA1), mRNA.	GJAL; DFNB38; CX43; OD	0.00511737	3.49804
ILMN_1801077	KDELR2	7	Homo sapiens KDEL (Lys-Asp-Glu-Leu) endoplasmic reticulum protein retention receptor 2 (KDELR2), transcript variant 1, mRNA.	ELP-1; ERD2.2	0.0198852	3.48751
ILMN_1775931	DTX3L	3	Homo sapiens deltex 3-like (Drosophila) (DTX3L), mRNA.	BBAP	0.0157886	3.4862
ILMN_1778924	SOX9	17	Homo sapiens SRY (sex determining region Y)-box 9 (campomelic dysplasia, autosomal sex-reversal) (SOX9), mRNA.	CMPD1; CMD1; SRA1	0.0118169	3.48593
ILMN_1659913	SERPINA10	14	Homo sapiens serpin peptidase inhibitor, clade A (alpha-1 antiproteinase, antitrypsin), member 10 (SERPINA10), transcript variant 1, mRNA.	PZI; ZPI	0.0173188	3.48355

ILMN_1653203	C20orf121	20	Homo sapiens chromosome 20 open reading frame 121 (C20orf121), transcript variant 1, mRNA.	DKFZp686E0870; MGC24	0.00264013	3.47969
ILMN_1781010	ZFPM2	8	Homo sapiens zinc finger protein, multitype 2 (ZFPM2), mRNA.	DIH3; ZNF89B; FOG2; hF	0.00719513	3.46297
ILMN_1652631	DPH5	1	Homo sapiens DPH5 homolog (S. cerevisiae) (DPH5), transcript variant 3, mRNA.	MGC61450; AD-018; NPD	0.00139887	3.45134
ILMN_1716397	POLE2	14	Homo sapiens polymerase (DNA directed), epsilon 2 (p59 subunit) (POLE2), mRNA.	DPE2	0.032266	3.44745
ILMN_2197365	PTGS2	1	Homo sapiens prostaglandin-endoperoxide synthase 2 (prostaglandin G/H synthase and cyclooxygenase) (PTGS2), mRNA.	PHS-2; COX-2; hCox-2; C	0.00397639	3.43995
ILMN_1778723	VEGFC	4	Homo sapiens vascular endothelial growth factor C (VEGFC), mRNA.	VRP; Flt4-L	0.0042059	3.42198
ILMN_1662419	C21orf33	21	Homo sapiens chromosome 21 open reading frame 33 (C21orf33), nuclear gene encoding mitochondrial protein, transcript variant 1, mRNA.	ES1; GT335; HES1; KNP-	0.00267637	3.42088
ILMN_1808404	ITGB2	21	Homo sapiens integrin, beta 2 (complement component 3 receptor 3 and 4 subunit) (ITGB2), mRNA.	MAC-1; LFA-1; MF17; LC/	0.00607652	3.41776
ILMN_2125747	WAS	X	Homo sapiens Wiskott-Aldrich syndrome (eczema-thrombocytopenia) (WAS), mRNA.	WASP; IMD2; THC	0.0146269	3.41691
ILMN_2395375	HLA-H	6	Homo sapiens major histocompatibility complex, class I, H (pseudogene) (HLA-H) on chromosome 6.	HLAHP	0.0125307	3.41456

ILMN_2347145	VSNL1	2	Homo sapiens visinin-like 1 (VSNL1), mRNA.	HPCAL3; VILIP-1; HUVISL	0.0154167	3.41378
ILMN_1757552	ALDH1A3	15	Homo sapiens aldehyde dehydrogenase 1 family, member A3 (ALDH1A3), mRNA.	RALDH3; ALDH6; ALDH1A	0.00365564	3.40622
ILMN_1756992	MAOA	X	Homo sapiens monoamine oxidase A (MAOA), nuclear gene encoding mitochondrial protein, mRNA.		0.0353614	3.39124
ILMN_1815652	SLC25A26	3	Homo sapiens solute carrier family 25, member 26 (SLC25A26), nuclear gene encoding mitochondrial protein, mRNA.	SAMC; DKFZp434E079; F	0.0362036	3.38503
ILMN_1795089	HLA-DMA	6	Homo sapiens major histocompatibility complex, class II, DM alpha (HLA-DMA), mRNA.	HLADM; RING6; DMA; D6	0.018532	3.38201
ILMN_1670490	KLHL17	1	Homo sapiens kelch-like 17 (Drosophila) (KLHL17), mRNA.	RP11-54O7.6	0.00483998	3.37282
ILMN_1659936	MT1F	16	Homo sapiens metallothionein 1F (MT1F), mRNA.	MT1; MGC32732	0.0235234	3.3669
ILMN_1664362	FGL1	8	Homo sapiens fibrinogen-like 1 (FGL1), transcript variant 1, mRNA.	LFIRE1; HP-041; MGC124	0.00313359	3.35019
ILMN_2376859	SLC2A6	9	Homo sapiens solute carrier family 2 (facilitated glucose transporter), member 6 (SLC2A6), mRNA.	GLUT6; HSA011372; GLU	0.0471558	3.34416
ILMN_1807439	CDH11	16	Homo sapiens cadherin 11, type 2, OB-cadherin (osteoblast) (CDH11), mRNA.	CDHOB; CAD11; OB; OSF	0.0104832	3.34181
ILMN_1724718	MYL9	20	Homo sapiens myosin, light chain 9, regulatory (MYL9), transcript variant 1, mRNA.	LC20; MYRL2; MRLC1; MI	0.0174655	3.33683
ILMN_1813139			Homo sapiens mRNA; cDNA DKFZp434C128 (from clone DKFZp434C128)		0.00572145	3.3342

ILMN_1710937	LOC730994		PREDICTED: Homo sapiens similar to NACHT, leucine rich repeat and PYD (pyrin domain) containing 1, transcript variant 1 (LOC730994), mRNA.		0.0139195	3.33398
ILMN_1699931	GYG2	X	Homo sapiens glycogenin 2 (GYG2), transcript variant 2, mRNA.	GN-2; GN2	0.0491526	3.32517
ILMN_1700310	NKD2	5	Homo sapiens naked cuticle homolog 2 (Drosophila) (NKD2), mRNA.	Naked2	0.0401528	3.31883
ILMN_1765109	FLJ14213	11	Homo sapiens protor-2 (FLJ14213), mRNA.	MGC16218	0.00649338	3.3113
ILMN_1666819	EI24	11	Homo sapiens etoposide induced 2.4 mRNA (EI24), transcript variant 1, mRNA.	TP53I8; PIG8	0.00724179	3.30037
ILMN_1803094	FAM131A	3	Homo sapiens family with sequence similarity 131, member A (FAM131A), mRNA.	MGC21688; PRO1378; FL	0.0148076	3.29858
ILMN_1721876	COL6A1	21	Homo sapiens collagen, type VI, alpha 1 (COL6A1), mRNA.	OPLL	0.0165028	3.2978
ILMN_2231928	LOC652493		PREDICTED: Homo sapiens similar to Ig kappa chain V-I region HK102 precursor (LOC652493), mRNA.		0.0290279	3.2805
ILMN_1736939	C8orf70	8	Homo sapiens chromosome 8 open reading frame 70 (C8orf70), mRNA.	CGI-62	0.00929602	3.2747
ILMN_1769575	GATS	7	Homo sapiens opposite strand transcription unit to STAG3 (GATS), mRNA.	DKFZp686B07267	0.000652331	3.26857
ILMN_2208413	CLEC2B	12	Homo sapiens C-type lectin domain family 2, member B (CLEC2B), mRNA.	AICL; IFNRG1; HP10085;	0.0201189	3.26228
ILMN_1754969	TGFB3	14	Homo sapiens transforming growth factor, beta 3 (TGFB3), mRNA.	FLJ16571; ARVD; TGF-be	0.00192331	3.26149

ILMN_2373791	ENPP2	8	Homo sapiens ectonucleotide pyrophosphatase/phosphodiesterase 2 (autotaxin) (ENPP2), transcript variant 2, mRNA.	NPP2; ATX; LysoPLD; ATX	0.0266877	3.2608
ILMN_1704554	HAMP	19	Homo sapiens hepcidin antimicrobial peptide (HAMP), mRNA.	HFE2B; LEAP1; LEAP-1; H	0.00168964	3.25383
ILMN_1814194	ENPP5	6	Homo sapiens ectonucleotide pyrophosphatase/phosphodiesterase 5 (putative function) (ENPP5), mRNA.	KIAA0879	0.00357115	3.25364
ILMN_2181892	HM13	20	Homo sapiens histocompatibility (minor) 13 (HM13), transcript variant 2, mRNA.	MSTP086; H13; IMPAS; S	0.0423547	3.25187
ILMN_1769926	COL15A1	9	Homo sapiens collagen, type XV, alpha 1 (COL15A1), mRNA.	FLJ38566	0.0415359	3.25061
ILMN_2157441	LOC728358	8	Homo sapiens defensin, alpha 1 (LOC728358), mRNA.		0.027375	3.24604
ILMN_2376847	BCL11A	2	Homo sapiens B-cell CLL/lymphoma 11A (zinc finger protein) (BCL11A), transcript variant 1, mRNA.	BCL11A-L; CTIP1; FLJ101	0.00694029	3.23678
ILMN_1691508	FCGR2A	1	Homo sapiens Fc fragment of IgG, low affinity IIa, receptor (CD32) (FCGR2A), mRNA.	FCGR2A1; CDw32; CD32/	0.00122377	3.23427
ILMN_1675062	TGFBI	5	Homo sapiens transforming growth factor, beta-induced, 68kDa (TGFBI), mRNA.	CSD1; CSD2; CDB1; CDG	0.00225935	3.22982
ILMN_1735192	RGS11	16	Homo sapiens regulator of G-protein signaling 11 (RGS11), transcript variant 1, mRNA.	RS11	0.0449565	3.22368
ILMN_1710209	DHODH	16	Homo sapiens dihydroorotate dehydrogenase (DHODH), nuclear gene encoding mitochondrial protein, transcript variant 2, mRNA.	DHOdehase	0.00816658	3.21572

ILMN_1698732	MMP23B	1	Homo sapiens matrix metallopeptidase 23B (MMP23B), mRNA.	MIFR-1; MIFR; MMP22	0.012499	3.21561
ILMN_2137789	NR2F6	19	Homo sapiens nuclear receptor subfamily 2, group F, member 6 (NR2F6), mRNA.	EAR-2; EAR2; ERBAL2	0.0014239	3.21284
ILMN_1748281	SLC25A12	2	Homo sapiens solute carrier family 25 (mitochondrial carrier, Aralar), member 12 (SLC25A12), nuclear gene encoding mitochondrial protein, mRNA.	ARALAR1; ARALAR	0.0340309	3.20947
ILMN_1676088	PCSK9	1	Homo sapiens proprotein convertase subtilisin/kexin type 9 (PCSK9), mRNA.	NARC1; NARC-1; HCHOLA	0.0018674	3.20732
ILMN_1790859	WISP1	8	Homo sapiens WNT1 inducible signaling pathway protein 1 (WISP1), transcript variant 1, mRNA.	WISP1tc; WISP1i; WISP1	0.000655317	3.20655
ILMN_2254635	GSTM1	1	Homo sapiens glutathione S-transferase M1 (GSTM1), transcript variant 1, mRNA.	MGC26563; GSTM1-1; H-	0.00535395	3.202
ILMN_1789096	ARMET	3	Homo sapiens arginine-rich, mutated in early stage tumors (ARMET), mRNA.	MANF; MGC142150; ARP:	0.0159442	3.20135
ILMN_1801119	MPO	17	Homo sapiens myeloperoxidase (MPO), nuclear gene encoding mitochondrial protein, mRNA.		0.029587	3.19845
ILMN_2415179	MX1	21	Homo sapiens myxovirus (influenza virus) resistance 1, interferon-inducible protein p78 (mouse) (MX1), mRNA.	MxA; IFI78; MX; IFI-78K	0.00601598	3.19151
ILMN_1663080	ZBTB46	20	Homo sapiens zinc finger and BTB domain containing 46 (ZBTB46), mRNA.	BTBD4; ZNF340; FLJ1350	1.62E-05	3.17081
ILMN_1764266	UCRC	22	Homo sapiens ubiquinol-cytochrome c reductase complex (7.2 kD) (UCRC), transcript variant 1, mRNA.	HSPC151; HSPC051; HSP	0.0364089	3.15348

ILMN_1673363	MAPKAP1	9	Homo sapiens mitogen-activated protein kinase associated protein 1 (MAPKAP1), transcript variant 1, mRNA.	MGC2745; MIP1; SIN1b;	0.00603643	3.15236
ILMN_2218208	BBOX1	11	Homo sapiens butyrobetaine (gamma), 2-oxoglutarate dioxygenase (gamma-butyrobetaine hydroxylase) 1 (BBOX1), mRNA.	G-BBH; BBOX; gamma-B	0.00286577	3.14109
ILMN_2405009	ADH5	4	Homo sapiens alcohol dehydrogenase 5 (class III), chi polypeptide (ADH5), mRNA.	ADH-3; ADHX; FDH	0.0027022	3.12901
ILMN_1752520	CD163	12	Homo sapiens CD163 molecule (CD163), transcript variant 2, mRNA.	MM130; M130	0.0114604	3.12242
ILMN_1721770	SHMT1	17	Homo sapiens serine hydroxymethyltransferase 1 (soluble) (SHMT1), transcript variant 1, mRNA.	MGC15229; MGC24556; S	0.024263	3.12196
ILMN_2340259	S100A10	1	Homo sapiens S100 calcium binding protein A10 (annexin II ligand, calpactin I, light polypeptide (p11)) (S100A10), mRNA.	ANX2L; MGC111133; ANX	0.00579303	3.12178
ILMN_1701204	CSRP1	1	Homo sapiens cysteine and glycine-rich protein 1 (CSRP1), mRNA.	CRP; CRP1; DKFZp686M1	0.0113099	3.12027
ILMN_2407389	C8orf38	8	Homo sapiens chromosome 8 open reading frame 38 (C8orf38), mRNA.	MGC40214	0.0301294	3.10865
ILMN_1801504	TMEM177	2	Homo sapiens transmembrane protein 177 (TMEM177), mRNA.	MGC10993	0.000548708	3.10125
ILMN_1668063	LOC642755	15	PREDICTED: Homo sapiens similar to DEXI, transcript variant 1 (LOC642755), mRNA.		0.0261742	3.09984

ILMN_1655796	SLC20A1	2	Homo sapiens solute carrier family 20 (phosphate transporter), member 1 (SLC20A1), mRNA.	Glvr-1; PiT-1; FLJ41426;	0.027237	3.09854
ILMN_1717557	C4BPB	1	Homo sapiens complement component 4 binding protein, beta (C4BPB), transcript variant 5, mRNA.	C4BP	0.0271004	3.0985
ILMN_1688780	MGP	12	Homo sapiens matrix Gla protein (MGP), mRNA.	NTI; GIG36; MGLAP	0.016416	3.09264
ILMN_1770290	MYLIP	6	Homo sapiens myosin regulatory light chain interacting protein (MYLIP), mRNA.	MIR	0.0106294	3.07787
ILMN_1751851	LOC729085	3	PREDICTED: Homo sapiens hypothetical protein LOC729085 (LOC729085), mRNA.		0.0153343	3.07581
ILMN_1770338	NRP1	10	Homo sapiens neuropilin 1 (NRP1), transcript variant 1, mRNA.	DKFZp781F1414; VEGF16	0.0152799	3.07571
ILMN_1788019	FLNC	7	Homo sapiens filamin C, gamma (actin binding protein 280) (FLNC), mRNA.	FLN2; ABPL; ABPA; FLJ10	0.0360008	3.07444
ILMN_1782788	SEPX1	16	Homo sapiens selenoprotein X, 1 (SEPX1), mRNA.	MSRB1; SELR; MGC3344;	0.00248361	3.07365
ILMN_1654735	SLC30A1	1	Homo sapiens solute carrier family 30 (zinc transporter), member 1 (SLC30A1), mRNA.	ZNT1; ZRC1	0.00706931	3.07207
ILMN_1727532	OSBPL5	11	Homo sapiens oxysterol binding protein-like 5 (OSBPL5), transcript variant 2, mRNA.	ORP5; FLJ42929; OBPH1	0.0434045	3.0687
ILMN_1693334	PTPRE	10	Homo sapiens protein tyrosine phosphatase, receptor type, E (PTPRE), transcript variant 2, mRNA.	DKFZp313F1310; PTPE; H	0.0367033	3.04901
ILMN_2145116	C11orf9	11	Homo sapiens chromosome 11 open reading frame 9 (C11orf9), mRNA.	MGC10781; KIAA0954	0.00452912	3.03204

ILMN_1777325	RELB	19	Homo sapiens v-rel reticuloendotheliosis viral oncogene homolog B, nuclear factor of kappa light polypeptide gene enhancer in B-cells 3 (avian) (RELB), mRNA.	I-REL	0.0242155	3.01611
ILMN_1785202	THPO	3	Homo sapiens thrombopoietin (myeloproliferative leukemia virus oncogene ligand, megakaryocyte growth and development factor) (THPO), mRNA.	TPO; MPLLG; MGC163194	0.0183557	3.00537
ILMN_1692058	RNASE2	14	Homo sapiens ribonuclease, RNase A family, 2 (liver, eosinophil-derived neurotoxin) (RNASE2), mRNA.	EDN; RNS2	0.00811795	3.00518
ILMN_1661755	MT1H	16	Homo sapiens metallothionein 1H (MT1H), mRNA.	MGC70702; MT1	0.0145562	2.99956
ILMN_1702636	PPIL1	6	Homo sapiens peptidylprolyl isomerase (cyclophilin)-like 1 (PPIL1), mRNA.	hCyPX; PPIase; CYPL1; C	0.0148831	2.99916
ILMN_1665792	CD163	12	Homo sapiens CD163 molecule (CD163), transcript variant 1, mRNA.	MM130; M130	0.0226717	2.99191
ILMN_2206746	SERPINA1	14	Homo sapiens serpin peptidase inhibitor, clade A (alpha-1 antiproteinase, antitrypsin), member 1 (SERPINA1), transcript variant 2, mRNA.	PI1; MGC23330; PRO227	0.00878347	2.99175
ILMN_2391150			Homo sapiens cDNA clone IMAGE:4830466		0.0184754	2.99009
ILMN_1697377	SLIT2	4	Homo sapiens slit homolog 2 (Drosophila) (SLIT2), mRNA.	Slit-2; SLIL3; FLJ14420	0.0105146	2.99002
ILMN_2153916	CRYAA	21	Homo sapiens crystallin, alpha A (CRYAA), mRNA.	HSPB4; CRYA1	0.00199213	2.987

ILMN_2309848	LOC652694		PREDICTED: Homo sapiens similar to Ig kappa chain V-I region HK102 precursor (LOC652694), mRNA.		0.00839992	2.98389
ILMN_1676893	ABCA1	9	Homo sapiens ATP-binding cassette, sub-family A (ABC1), member 1 (ABCA1), mRNA.	CERP; ABC-1; MGC16486	0.00229249	2.98155
ILMN_1752502	TRIB3	20	Homo sapiens tribbles homolog 3 (Drosophila) (TRIB3), mRNA.	TRB3; C20orf97; SKIP3;	0.0428968	2.9788
ILMN_1719641	FGF9	13	Homo sapiens fibroblast growth factor 9 (glia-activating factor) (FGF9), mRNA.	MGC119914; GAF; MGC1	0.0185803	2.97829
ILMN_1811258	NTN1	17	Homo sapiens netrin 1 (NTN1), mRNA.	NTN1L	0.00639808	2.97768
ILMN_1717934	EMILIN1	2	Homo sapiens elastin microfibril interfacer 1 (EMILIN1), mRNA.	EMILIN-1; EMILIN; gp115	0.00141482	2.97685
ILMN_2258816	LOC644334		PREDICTED: Homo sapiens similar to Band 4.1-like protein 5 (LOC644334), mRNA.		0.0214141	2.96756
ILMN_1725387	SLC45A4	8	PREDICTED: Homo sapiens solute carrier family 45, member 4, transcript variant 2 (SLC45A4), mRNA.		0.003121	2.96722
ILMN_1719986	TMEM99	17	Homo sapiens transmembrane protein 99 (TMEM99), mRNA.	MGC21518	0.0431822	2.9621
ILMN_1785646	RNF19A	8	Homo sapiens ring finger protein 19A (RNF19A), transcript variant 2, mRNA.	RNF19; DKFZp566B1346	0.014502	2.95884
ILMN_1717163	FAM86B1	8	Homo sapiens family with sequence similarity 86, member B1 (FAM86B1), transcript variant 1, mRNA.	MGC16279; MGC104828	0.0110616	2.95732

ILMN_1801766	GALNTL1	14	Homo sapiens UDP-N-acetyl-alpha-D-galactosamine:polypeptide N-acetylgalactosaminyltransferase-like 1 (GALNTL1), mRNA.	MGC141855; GALNT16; K	0.0342797	2.95709
ILMN_1771376	NUDT6	4	Homo sapiens nudix (nucleoside diphosphate linked moiety X)-type motif 6 (NUDT6), transcript variant 1, mRNA.	ASFGF2; gfg-1; FGF-AS;	0.000969952	2.94705
ILMN_1801043	C2orf40	2	Homo sapiens chromosome 2 open reading frame 40 (C2orf40), mRNA.	ECRG4	0.0107084	2.93389
ILMN_2149226	FAM20A	17	Homo sapiens family with sequence similarity 20, member A (FAM20A), mRNA.	DKFZp434F2322; FP2747	0.00200622	2.93341
ILMN_1652371	GRPEL1	4	Homo sapiens GrpE-like 1, mitochondrial (E. coli) (GRPEL1), nuclear gene encoding mitochondrial protein, mRNA.	HMGE; FLJ25609	0.00723447	2.93182
ILMN_2337655	LAX1	1	Homo sapiens lymphocyte transmembrane adaptor 1 (LAX1), mRNA.	LAX; FLJ20340	0.000585899	2.92714
ILMN_2332553	NTHL1	16	Homo sapiens nth endonuclease III-like 1 (E. coli) (NTHL1), mRNA.	NTH1; OCTS3	0.0115734	2.92129
ILMN_1750101	IGSF9	1	Homo sapiens immunoglobulin superfamily, member 9 (IGSF9), mRNA.	KIAA1355; FP18798; Nrt1	0.0327728	2.92007
ILMN_1744487	NR4A2	2	Homo sapiens nuclear receptor subfamily 4, group A, member 2 (NR4A2), transcript variant 1, mRNA.	RNR1; NOT; TINUR; NUR	0.0299863	2.91835
ILMN_1807339	HOPX	4	Homo sapiens HOP homeobox (HOPX), transcript variant 3, mRNA.	HOP; MGC20820; LAGY;	0.0121983	2.91485
ILMN_1766261	PRKCDBP	11	Homo sapiens protein kinase C, delta binding protein (PRKCDBP), mRNA.	HSRBC; MGC20400; SRB	0.00951458	2.91338

ILMN_2150654	ACAT2	6	Homo sapiens acetyl-Coenzyme A acetyltransferase 2 (ACAT2), mRNA.		0.0121772	2.90872
ILMN_2046730	3-Mar	5	Homo sapiens membrane-associated ring finger (C3HC4) 3 (MARCH3), mRNA.	RNF173; MARCH-III; MGC	0.0165909	2.90864
ILMN_1815154	ARNTL	11	Homo sapiens aryl hydrocarbon receptor nuclear translocator-like (ARNTL), transcript variant 2, mRNA.	PASD3; MOP3; BMAL1; M	0.0106787	2.90722
ILMN_1811921	ITGAX	16	Homo sapiens integrin, alpha X (complement component 3 receptor 4 subunit) (ITGAX), mRNA.	CD11C	0.000687516	2.89764
ILMN_1751886	PMM2	16	Homo sapiens phosphomannomutase 2 (PMM2), mRNA.	CDGS; CDG1a; CDG1	0.0191243	2.89404
ILMN_1652237	XLKD1	11	Homo sapiens extracellular link domain containing 1 (XLKD1), mRNA.	CRSBP-1; LYVE-1; HAR	0.00354995	2.8912
ILMN_2082130	STAG3	7	Homo sapiens stromal antigen 3 (STAG3), mRNA.		0.00321211	2.8861
ILMN_1732296	GPAM	10	Homo sapiens glycerol-3-phosphate acyltransferase, mitochondrial (GPAM), nuclear gene encoding mitochondrial protein, mRNA.	GPAT1; MGC26846; KIAA	0.0185042	2.88091
ILMN_1667081			602629864F1 NCI_CGAP_Skn4 Homo sapiens cDNA clone IMAGE:4754918 5, mRNA sequence		0.0112636	2.87767
ILMN_1715647	HES4	1	Homo sapiens hairy and enhancer of split 4 (Drosophila) (HES4), mRNA.		0.0100825	2.87317
ILMN_1764228	BHLHB3	12	Homo sapiens basic helix-loop-helix domain containing, class B, 3 (BHLHB3), mRNA.	SHARP1; SHARP-1; DEC2	0.00186983	2.87225

ILMN_1802654	ADRA2A	10	Homo sapiens adrenergic, alpha-2A-, receptor (ADRA2A), mRNA.	ZNF32; ALPHA2AAR; ADR	0.00328951	2.86985
ILMN_1660364	NHEDC2	4	Homo sapiens Na+/H+ exchanger domain containing 2 (NHEDC2), mRNA.	FLJ23984; NHA2	0.000659049	2.86909
ILMN_1674706	TFB2M	1	Homo sapiens transcription factor B2, mitochondrial (TFB2M), nuclear gene encoding mitochondrial protein, mRNA.	FLJ22661; Hkp1; FLJ2318	0.0121116	2.86776
ILMN_2206722	RBMS3	3	Homo sapiens RNA binding motif, single stranded interacting protein (RBMS3), transcript variant 1, mRNA.		0.00910096	2.86751
ILMN_1676528	LILRA3	19	Homo sapiens leukocyte immunoglobulin-like receptor, subfamily A (without TM domain), member 3 (LILRA3), mRNA.	HM31; LIR-4; e3; CD85E	0.0249768	2.86256
ILMN_1701875	ENG	9	Homo sapiens endoglin (Osler-Rendu-Weber syndrome 1) (ENG), mRNA.	ORW; CD105; HHT1; END	0.00737917	2.85861
ILMN_1769282	ASGR2	17	Homo sapiens asialoglycoprotein receptor 2 (ASGR2), transcript variant H2', mRNA.	L-H2; CLEC4H2; Hs.1259	0.00754756	2.85826
ILMN_1803429	ALDH1A1	9	Homo sapiens aldehyde dehydrogenase 1 family, member A1 (ALDH1A1), mRNA.	ALDH11; ALDH-E1; ALDC	0.00187591	2.8562
ILMN_2157240	CBLB	3	Homo sapiens Cas-Br-M (murine) ecotropic retroviral transforming sequence b (CBLB), mRNA.	DKFZp779F1443; FLJ411	0.00084686	2.85202
ILMN_1723978	LRRN2	1	Homo sapiens leucine rich repeat neuronal 2 (LRRN2), transcript variant 2, mRNA.	FIGLER7; GAC1; LRRN5;	0.00919083	2.85045

ILMN_1804798	GCSH	16	Homo sapiens glycine cleavage system protein H (aminomethyl carrier) (GCSH), mRNA.	NKH; GCE	0.0164526	2.84929
ILMN_1739794	GFOD2	16	Homo sapiens glucose-fructose oxidoreductase domain containing 2 (GFOD2), mRNA.	FLJ23802; MGC11335	0.0421923	2.84903
ILMN_1733538	NDN	15	Homo sapiens necdin homolog (mouse) (NDN), mRNA.	HsT16328	0.00531437	2.84812
ILMN_2128428	NEIL2	8	Homo sapiens nei like 2 (E. coli) (NEIL2), mRNA.	FLJ31644; MGC4505; MG	9.37E-05	2.848
ILMN_1810725	CCNL1	3	Homo sapiens cyclin L1 (CCNL1), mRNA.	ania-6a; PRO1073; BM-0(	0.00103669	2.84725
ILMN_1684306	SLC26A3	7	Homo sapiens solute carrier family 26, member 3 (SLC26A3), mRNA.	CLD; DRA	0.029315	2.83528
ILMN_1683664	CD93	20	Homo sapiens CD93 molecule (CD93), mRNA.	MXRA4; C1qR(P); dJ737E	0.0120647	2.83417
ILMN_1810836	ICAM3	19	Homo sapiens intercellular adhesion molecule 3 (ICAM3), mRNA.	ICAM-R; CD50; CDW50	0.00368945	2.83195
ILMN_1811437	DNAJC19	3	Homo sapiens DnaJ (Hsp40) homolog, subfamily C, member 19 (DNAJC19), mRNA.	TIM14; TIMM14	0.0373635	2.83133
ILMN_1788017	DDC	7	Homo sapiens dopa decarboxylase (aromatic L-amino acid decarboxylase) (DDC), mRNA.	AADC	0.0110469	2.82966
ILMN_1844029	SIDT1	3	Homo sapiens SID1 transmembrane family, member 1 (SIDT1), mRNA.	SID1; B830021E24Rik; Fl	0.033752	2.81498
ILMN_1660904	SLC2A10	20	Homo sapiens solute carrier family 2 (facilitated glucose transporter), member 10 (SLC2A10), mRNA.	MGC126706; ATS; GLUT1	0.0292806	2.81477
ILMN_1731714	ANGPT1	8	Homo sapiens angiopoietin 1 (ANGPT1), mRNA.	ANG1; AGP1; AGPT	0.0199838	2.80932

ILMN_1658498	COQ3	6	Homo sapiens coenzyme Q3 homolog, methyltransferase (S. cerevisiae) (COQ3), mRNA.	bA9819.1; UG0215E05	0.0270768	2.80876
ILMN_1809928	SPINT2	19	Homo sapiens serine peptidase inhibitor, Kunitz type, 2 (SPINT2), mRNA.	Kop; PB; HAI-2; HAI2	0.0493912	2.80845
ILMN_1738147	ACOT1	14	Homo sapiens acyl-CoA thioesterase 1 (ACOT1), mRNA.	LACH2; ACH2; CTE-1	0.0120939	2.80213
ILMN_2190414	DBN1	5	Homo sapiens drebrin 1 (DBN1), transcript variant 1, mRNA.	DKFZp434D064; D0S117	0.00521392	2.7958
ILMN_1808590	CMTM8	3	Homo sapiens CKLF-like MARVEL transmembrane domain containing 8 (CMTM8), mRNA.	CKLFSF8; CKLFSF8-V2	0.023154	2.76839
ILMN_2285404			BX104232 Soares fetal liver spleen 1NFLS Homo sapiens cDNA clone IMAGp998L09114, mRNA sequence		0.0164482	2.7626
ILMN_1812392	CPVL	7	Homo sapiens carboxypeptidase, vitellogenic-like (CPVL), transcript variant 1, mRNA.	HVLP; MGC10029	0.00334629	2.75945
ILMN_1783304	GPER	7	Homo sapiens G protein-coupled estrogen receptor 1 (GPER), transcript variant 3, mRNA.	LyGPR; DRY12; GPCR-Br;	0.00445667	2.75911
ILMN_1672503	VLDLR	9	Homo sapiens very low density lipoprotein receptor (VLDLR), transcript variant 2, mRNA.	FLJ35024; VLDLRCH	0.00921	2.7589
ILMN_1734543	TAGLN	11	Homo sapiens transgelin (TAGLN), transcript variant 2, mRNA.	TAGLN1; WS3-10; SM22;	0.000775196	2.75733
ILMN_2376822	CAMP	3	Homo sapiens cathelicidin antimicrobial peptide (CAMP), mRNA.	LL37; FALL39; CAP18; HS	0.0344462	2.75648

ILMN_1693338	HPS5	11	Homo sapiens Hermansky-Pudlak syndrome 5 (HPS5), transcript variant 2, mRNA.	KIAA1017; AIBP63	0.0181176	2.7559
ILMN_1808500	IMPA2	18	Homo sapiens inositol(myo)-1(or 4)-monophosphatase 2 (IMPA2), mRNA.		0.000384648	2.75386
ILMN_2366041	IL1RAP	3	Homo sapiens interleukin 1 receptor accessory protein (IL1RAP), transcript variant 2, mRNA.	FLJ37788; C3orf13; IL1R:	0.0159579	2.75076
ILMN_1713561	PACSIN3	11	Homo sapiens protein kinase C and casein kinase substrate in neurons 3 (PACSIN3), mRNA.	SDPIII	0.00318316	2.74895
ILMN_1705442	C1orf123	1	Homo sapiens chromosome 1 open reading frame 123 (C1orf123), mRNA.	FLJ20580; RP5-1024G6.3	0.00249218	2.74874
ILMN_1660923	GRM8	7	Homo sapiens glutamate receptor, metabotropic 8 (GRM8), mRNA.	FLJ41058; GLUR8; mGlu8	0.0276038	2.74282
ILMN_1761968	KIAA0363		PREDICTED: Homo sapiens KIAA0363 protein (KIAA0363), mRNA.		0.000443203	2.74273
ILMN_2138765	BTN3A3	6	Homo sapiens butyrophilin, subfamily 3, member A3 (BTN3A3), transcript variant 2, mRNA.	BTF3	0.0243279	2.74209
ILMN_1729117	FAHD2A	2	Homo sapiens fumarylacetoacetate hydrolase domain containing 2A (FAHD2A), mRNA.	MGC131995; CGI-105	0.00725412	2.73584
ILMN_1802109	CLEC4M	19	Homo sapiens C-type lectin domain family 4, member M (CLEC4M), transcript variant 6, mRNA.	MGC129964; DCSIGNR; D	0.0120583	2.73319
ILMN_2415235	TRIM15	6	Homo sapiens tripartite motif-containing 15 (TRIM15), mRNA.	ZNF178; ZNFB7; RNF93	0.00933103	2.72367

ILMN_2128770	CD74	5	Homo sapiens CD74 molecule, major histocompatibility complex, class II invariant chain (CD74), transcript variant 2, mRNA.	Ia-GAMMA; DHLAG; HLAD	0.0337353	2.72262
ILMN_1764571	SLC7A6	16	Homo sapiens solute carrier family 7 (cationic amino acid transporter, y+ system), member 6 (SLC7A6), transcript variant 2, mRNA.	DKFZp686K15246; KIAA(	0.000917993	2.71016
ILMN_1677827	CHRDL2	11	Homo sapiens chordin-like 2 (CHRDL2), mRNA.	DKFZp586N2124; CHL2;	0.000555632	2.70596
ILMN_1676822	SLC2A10	20	Homo sapiens solute carrier family 2 (facilitated glucose transporter), member 10 (SLC2A10), mRNA.	MGC126706; ATS; GLUT1	0.00629137	2.70289
ILMN_2348788	UBASH3A	21	Homo sapiens ubiquitin associated and SH3 domain containing, A (UBASH3A), transcript variant 2, mRNA.	TULA; CLIP4; STS-2	0.0040962	2.69983
ILMN_1732151	COX7A1	19	Homo sapiens cytochrome c oxidase subunit VIIa polypeptide 1 (muscle) (COX7A1), mRNA.	COX7AH; COX7A; COX7A	0.00765905	2.69935
ILMN_2088437	TSPAN4	11	Homo sapiens tetraspanin 4 (TSPAN4), transcript variant 6, mRNA.	NAG-2; NAG2; TETRASPA	0.00074567	2.69731
ILMN_1746465	HSPA12A	10	Homo sapiens heat shock 70kDa protein 12A (HSPA12A), mRNA.	KIAA0417; FLJ13874	0.0363864	2.68968
ILMN_1668822	PPP1R14A	19	Homo sapiens protein phosphatase 1, regulatory (inhibitor) subunit 14A (PPP1R14A), mRNA.	CPI-17; CPI17; PPP1INL	0.00619827	2.68938
ILMN_1781285	IRF8	16	Homo sapiens interferon regulatory factor 8 (IRF8), mRNA.	H-ICSBP; ICSBP; IRF-8; I	0.0265523	2.68785

ILMN_2400947	MOCS2	5	Homo sapiens molybdenum cofactor synthesis 2 (MOCS2), transcript variant 1, mRNA.	MPTS; MCBPE; MOCS2B;	0.0265285	2.68652
ILMN_1682332	PAQR8	6	Homo sapiens progestin and adipoQ receptor family member VIII (PAQR8), mRNA.	FLJ32521; LMPB1; FLJ46	0.0126777	2.68339
ILMN_1739496	ADARB1	21	Homo sapiens adenosine deaminase, RNA-specific, B1 (RED1 homolog rat) (ADARB1), transcript variant 1, mRNA.	ADAR2a; ADAR2; DRABA	0.0156538	2.67578
ILMN_2358560	HLA-DPB1	6	Homo sapiens major histocompatibility complex, class II, DP beta 1 (HLA-DPB1), mRNA.	HLA-DP1B; DPB1; MHC D	0.0491253	2.67028
ILMN_1672662	TPST1	7	Homo sapiens tyrosylprotein sulfotransferase 1 (TPST1), mRNA.		0.0289364	2.66202
ILMN_1687768	GNE	9	Homo sapiens glucosamine (UDP-N-acetyl)-2-epimerase/N-acetylmannosamine kinase (GNE), mRNA.	NM; IBM2; Uae1; DMRV;	0.00224263	2.65917
ILMN_1776188	SGSM2	17	Homo sapiens small G protein signaling modulator 2 (SGSM2), transcript variant 1, mRNA.	KIAA0397; RUTBC1; SGS	0.0118308	2.65635
ILMN_1754476	PDE1A	2	Homo sapiens phosphodiesterase 1A, calmodulin-dependent (PDE1A), transcript variant 2, mRNA.	HSPDE1A; MGC26303; H	0.0357286	2.65434
ILMN_1696657	HSD17B12	11	Homo sapiens hydroxysteroid (17-beta) dehydrogenase 12 (HSD17B12), mRNA.	KAR	0.0027941	2.65224
ILMN_1812618	MFGE8	15	Homo sapiens milk fat globule-EGF factor 8 protein (MFGE8), mRNA.	HsT19888; BA46; OAcGD	3.86E-05	2.65034
ILMN_1735038	TLN2	15	Homo sapiens talin 2 (TLN2), mRNA.	KIAA0320; ILWEQ; DKFZ	0.0191678	2.64732

ILMN_1774901	HSPA2	14	Homo sapiens heat shock 70kDa protein 2 (HSPA2), mRNA.		0.00296981	2.64513
ILMN_1740512	TAGLN	11	Homo sapiens transgelin (TAGLN), transcript variant 2, mRNA.	TAGLN1; WS3-10; SM22;	0.00247149	2.64375
ILMN_2370976	GPR83	11	Homo sapiens G protein-coupled receptor 83 (GPR83), mRNA.	GPR72; GIR	0.00398289	2.64337
ILMN_1773059	PDGFD	11	Homo sapiens platelet derived growth factor D (PDGFD), transcript variant 2, mRNA.	IEGF; SCDGF-B; MSTP03	0.0172593	2.64289
ILMN_1803392	ARHGAP15	2	Homo sapiens Rho GTPase activating protein 15 (ARHGAP15), mRNA.	BM046	0.0442094	2.63806
ILMN_1726368	CCDC146	7	Homo sapiens coiled-coil domain containing 146 (CCDC146), mRNA.	KIAA1505	0.00183178	2.63256
ILMN_1654518	SC4MOL	4	Homo sapiens sterol-C4-methyl oxidase-like (SC4MOL), transcript variant 2, mRNA.	MGC104344; ERG25; DE	0.0256258	2.62937
ILMN_1788192	ACSS2	20	Homo sapiens acyl-CoA synthetase short-chain family member 2 (ACSS2), transcript variant 2, mRNA.	AceCS; ACAS2; ACS; AC	0.01446	2.62517
ILMN_1660357	MBNL3	X	Homo sapiens muscleblind-like 3 (Drosophila) (MBNL3), transcript variant R, mRNA.	MBLX39; MBLX; FLJ1131	0.00854041	2.62257
ILMN_1716803	HSPB2	11	Homo sapiens heat shock 27kDa protein 2 (HSPB2), mRNA.	Hs.78846; LOH11CR1K;	0.0141188	2.61649
ILMN_1791494	RGS10	10	Homo sapiens regulator of G-protein signaling 10 (RGS10), transcript variant 1, mRNA.		0.0113724	2.61586
ILMN_1804339	SPON1	11	Homo sapiens spondin 1, extracellular matrix protein (SPON1), mRNA.	KIAA0762; MGC10724; f-	0.0070287	2.61583

ILMN_1772686	MRPL45	17	Homo sapiens mitochondrial ribosomal protein L45 (MRPL45), nuclear gene encoding mitochondrial protein, mRNA.	MGC11321	0.0498559	2.61321
ILMN_1795930	CES7	16	Homo sapiens carboxylesterase 7 (CES7), mRNA.	FLJ31547; CES4C1; CAUX	0.0236743	2.61229
ILMN_2404625	IFI6	1	Homo sapiens interferon, alpha-inducible protein 6 (IFI6), transcript variant 3, mRNA.	IFI-6-16; 6-16; IFI616; G	0.0181819	2.61
ILMN_2049766	ABCC11	16	Homo sapiens ATP-binding cassette, sub-family C (CFTR/MRP), member 11 (ABCC11), transcript variant 3, mRNA.	MRP8	0.0129333	2.60439
ILMN_1810214	C14orf2	14	Homo sapiens chromosome 14 open reading frame 2 (C14orf2), mRNA.	PLPM; MP68	0.0211083	2.5987
ILMN_1701441	PDK3	X	Homo sapiens pyruvate dehydrogenase kinase, isozyme 3 (PDK3), mRNA.		0.0238458	2.5961
ILMN_1796537	ACSS2	20	Homo sapiens acyl-CoA synthetase short-chain family member 2 (ACSS2), transcript variant 1, mRNA.	AceCS; ACAS2; ACS; DKF	0.0348529	2.59565
ILMN_2326512	TBC1D10C	11	Homo sapiens TBC1 domain family, member 10C (TBC1D10C), mRNA.	MGC46488; FLJ00332	0.0115893	2.59407
ILMN_2066066	VIL2	6	Homo sapiens villin 2 (ezrin) (VIL2), mRNA.	MGC1584; CVL; FLJ26216	0.0411776	2.59368
ILMN_1763941	BEX2	X	Homo sapiens brain expressed X-linked 2 (BEX2), mRNA.	BEX1; DJ79P11.1	0.000974141	2.58706
ILMN_2342066			BX098667 NCI_CGAP_Kid11 Homo sapiens cDNA clone IMAGp998B025926, mRNA sequence		0.0293664	2.58586

ILMN_1739805	SVEP1		Homo sapiens sushi, von Willebrand factor type A, EGF and pentraxin domain containing 1 (SVEP1), mRNA.	FLJ16013; POLYDOM; CC	0.000322507	2.58583
ILMN_1780825	KCNN2	5	Homo sapiens potassium intermediate/small conductance calcium-activated channel, subfamily N, member 2 (KCNN2), transcript variant 1, mRNA.	KCa2.2; SKCA2; SK2; hS	0.00898761	2.58578
ILMN_2169439	DHRS13	17	Homo sapiens dehydrogenase/reductase (SDR family) member 13 (DHRS13), mRNA.	MGC23280	0.0087191	2.58149
ILMN_1763891	S100A6	1	Homo sapiens S100 calcium binding protein A6 (S100A6), mRNA.	PRA; CABP; 2A9; 5B10; C	0.00724396	2.57939
ILMN_1795762	IGLL3	22	Homo sapiens immunoglobulin lambda-like polypeptide 3 (IGLL3), mRNA.	16.1	0.0356746	2.57883
ILMN_1745282	SLC17A2	6	Homo sapiens solute carrier family 17 (sodium phosphate), member 2 (SLC17A2), mRNA.	MGC138238; NPT3	0.0355649	2.57606
ILMN_1686679	GPR128	3	Homo sapiens G protein-coupled receptor 128 (GPR128), mRNA.	MGC163260; FLJ29035; M	0.0344448	2.57177
ILMN_1796712	HLA-DMB	6	Homo sapiens major histocompatibility complex, class II, DM beta (HLA-DMB), mRNA.	D6S221E; RING7	0.0211538	2.56787
ILMN_1795937	CD44	11	Homo sapiens CD44 molecule (Indian blood group) (CD44), transcript variant 4, mRNA.	LHR; CDW44; ECMR-III; I	0.00607625	2.56473
ILMN_1728677	SAR1B	5	Homo sapiens SAR1 gene homolog B (S. cerevisiae) (SAR1B), transcript variant 1, mRNA.	CMRD; SARA2; GTBPB	0.0120873	2.56002
ILMN_1768284	NES	1	Homo sapiens nestin (NES), mRNA.	FLJ21841; Nbla00170	0.0346878	2.55728

ILMN_2370208	GSTM2	1	Homo sapiens glutathione S-transferase M2 (muscle) (GSTM2), mRNA.	GST4; GSTM; GSTM2-2; (	0.000510039	2.55428
ILMN_1723048	ZNF135	19	Homo sapiens zinc finger protein 135 (ZNF135), mRNA.	ZNF61; pT3; pHZ-17; ZN	0.0223	2.55422
ILMN_1748751	AEBP1	7	Homo sapiens AE binding protein 1 (AEBP1), mRNA.	FLJ33612; ACLP	0.0144988	2.55392
ILMN_2081087	FANCD2	3	Homo sapiens Fanconi anemia, complementation group D2 (FANCD2), transcript variant 2, mRNA.	FACD; FA-D2; FA4; FANC	0.0152307	2.55136
ILMN_2095660	TM4SF4	3	Homo sapiens transmembrane 4 L six family member 4 (TM4SF4), mRNA.	ILTMP; il-TMP; FLJ31015	0.0394779	2.54839
ILMN_1756928	TSHZ3	19	Homo sapiens teashirt zinc finger homeobox 3 (TSHZ3), mRNA.	ZNF537; TSH3; KIAA147	0.0223212	2.54758
ILMN_1706261	CPZ	4	Homo sapiens carboxypeptidase Z (CPZ), transcript variant 1, mRNA.	MGC99682	0.019163	2.54428
ILMN_2140974	CYP1B1	2	Homo sapiens cytochrome P450, family 1, subfamily B, polypeptide 1 (CYP1B1), mRNA.	GLC3A; CP1B	0.0171865	2.5422
ILMN_2132809	BATF	14	Homo sapiens basic leucine zipper transcription factor, ATF-like (BATF), mRNA.	B-ATF; BATF1; SFA-2	0.00232362	2.54093
ILMN_1770940	TNFAIP3	6	Homo sapiens tumor necrosis factor, alpha-induced protein 3 (TNFAIP3), mRNA.	A20; MGC138687; OTUD	0.0087185	2.53523
ILMN_1666392			K-EST0216649 L17N670205n1 Homo sapiens cDNA clone L17N670205n1-10-D09 5, mRNA sequence		0.0455814	2.53267
ILMN_1762531	C1orf198	1	Homo sapiens chromosome 1 open reading frame 198 (C1orf198), mRNA.	FLJ14525; FLJ16283; DK	0.000877929	2.52812

ILMN_1773963	SERPINH1	11	Homo sapiens serpin peptidase inhibitor, clade H (heat shock protein 47), member 1, (collagen binding protein 1) (SERPINH1), mRNA.	RA-A47; AsTP3; PIG14; g	0.019896	2.528
ILMN_1793476	FABP5	8	Homo sapiens fatty acid binding protein 5 (psoriasis-associated) (FABP5), mRNA.	PA-FABP; PAFABP; E-FAB	0.0406499	2.52682
ILMN_1662731	SLC9A4	2	Homo sapiens solute carrier family 9 (sodium/hydrogen exchanger), member 4 (SLC9A4), mRNA.	NHE4; DKFZp313B031	0.0184358	2.52552
ILMN_2070052	ABHD8	19	Homo sapiens abhydrolase domain containing 8 (ABHD8), mRNA.	FLJ11743; MGC14280; M	0.015324	2.51881
ILMN_1775163	KIAA1949	6	Homo sapiens KIAA1949 (KIAA1949), mRNA.	HKMT1098	0.0194134	2.5171
ILMN_1690963	DNAJC12	10	Homo sapiens DnaJ (Hsp40) homolog, subfamily C, member 12 (DNAJC12), transcript variant 1, mRNA.	RP11-57G10.2; JDP1	0.00659293	2.50956
ILMN_1814305	P2RY5	13	Homo sapiens purinergic receptor P2Y, G-protein coupled, 5 (P2RY5), mRNA.	MGC120358; P2Y5	0.00806803	2.50264
ILMN_1824362	LOC642197		PREDICTED: Homo sapiens similar to Protein FAM82B (LOC642197), mRNA.		0.0127787	2.50237
ILMN_1767349	PDXK	21	Homo sapiens pyridoxal (pyridoxine, vitamin B6) kinase (PDXK), mRNA.	PKH; MGC52346; FLJ319	0.00481662	2.5014
ILMN_1680738	CMIP	16	Homo sapiens c-Maf-inducing protein (CMIP), transcript variant Tc-mip, mRNA.	KIAA1694	0.0414944	2.50087
ILMN_1663866	ADRA1A	8	Homo sapiens adrenergic, alpha-1A-, receptor (ADRA1A), transcript variant 3, mRNA.	ADRA1C; ALPHA1AAR; AD	0.00170817	2.5001
ILMN_1789558	CRELD2	22	Homo sapiens cysteine-rich with EGF-like domains 2 (CRELD2), mRNA.	DKFZp667O055; MGC112	0.00740768	2.49955

ILMN_2087702	TMEM136	11	Homo sapiens transmembrane protein 136 (TMEM136), mRNA.	MGC17839	0.00427529	2.49919
ILMN_1740493	LEAP-2	5	Homo sapiens liver-expressed antimicrobial peptide 2 (LEAP-2), mRNA.		0.0028095	2.49256
ILMN_2313901	GSTM1	1	Homo sapiens glutathione S-transferase M1 (GSTM1), transcript variant 1, mRNA.	MGC26563; GSTM1-1; H-	0.00319553	2.48823
ILMN_1774982	MSRB3	12	Homo sapiens methionine sulfoxide reductase B3 (MSRB3), transcript variant 1, mRNA.	FLJ36866; DKFZp686C11	0.00107601	2.48092
ILMN_2405684	CCL5	17	Homo sapiens chemokine (C-C motif) ligand 5 (CCL5), mRNA.	TCP228; SISd; MGC1716	0.000844306	2.47865
ILMN_1807972	SRC	20	Homo sapiens v-src sarcoma (Schmidt-Ruppin A-2) viral oncogene homolog (avian) (SRC), transcript variant 2, mRNA.	SRC1; p60-Src; c-SRC; A	0.00450023	2.47829
ILMN_1744604	FBLN1	22	Homo sapiens fibulin 1 (FBLN1), transcript variant C, mRNA.	FBLN	0.0476947	2.47788
ILMN_1796762	STAT3	17	Homo sapiens signal transducer and activator of transcription 3 (acute-phase response factor) (STAT3), transcript variant 1, mRNA.	APRF; FLJ20882; MGC16	0.00545168	2.47753
ILMN_1807359	CLIC5	6	Homo sapiens chloride intracellular channel 5 (CLIC5), mRNA.	MST130; MSTP130; FLJ9	0.0237916	2.47324
ILMN_2073446	TUG1	22	Homo sapiens taurine upregulated gene 1 (TUG1) on chromosome 22.	MGC46067; FLJ20618	0.04409	2.47203
ILMN_1780861	SC4MOL	4	Homo sapiens sterol-C4-methyl oxidase-like (SC4MOL), transcript variant 1, mRNA.	MGC104344; ERG25; DES	0.0218345	2.47122

ILMN_1668559	LOC650215		PREDICTED: Homo sapiens similar to Exportin-T (tRNA exportin) (Exportin(tRNA)) (LOC650215), mRNA.		0.0256843	2.46951
ILMN_1654778	MMACHC	1	Homo sapiens methylmalonic aciduria (cobalamin deficiency) cblC type, with homocystinuria (MMACHC), mRNA.	RP11-291L19.3; FLJ2567	0.0043004	2.46873
ILMN_1782922	NLRP3	1	Homo sapiens NLR family, pyrin domain containing 3 (NLRP3), transcript variant 3, mRNA.	AII/AVP; AGTAVPRL; PYP/	0.0461373	2.46794
ILMN_1674874	SLC9A1	1	Homo sapiens solute carrier family 9 (sodium/hydrogen exchanger), member 1 (antiporter, Na+/H+, amiloride sensitive) (SLC9A1), mRNA.	APNH; FLJ42224; NHE1	0.0174173	2.46482
ILMN_1801307	GUCY1A3	4	Homo sapiens guanylate cyclase 1, soluble, alpha 3 (GUCY1A3), mRNA.	GUCA3; GC-SA3; GUC1A3	0.0331712	2.46344
ILMN_2338348	PNKD	2	Homo sapiens paroxysmal nonkinesiogenic dyskinesia (PNKD), transcript variant 2, mRNA.	FKSG19; KIPP1184; DKFZ	0.0178641	2.46315
ILMN_1792455	CCNI	4	Homo sapiens cyclin I (CCNI), mRNA.	CYC1; CYI	0.0275032	2.45688
ILMN_2295518	SP110	2	Homo sapiens SP110 nuclear body protein (SP110), transcript variant b, mRNA.	FLJ22835; IFI75; VODI; I	0.0108666	2.45543
ILMN_1668039	CREB5	7	Homo sapiens cAMP responsive element binding protein 5 (CREB5), transcript variant 4, mRNA.	CRE-BPA	0.011207	2.45186
ILMN_1701933	DARC	1	Homo sapiens Duffy blood group, chemokine receptor (DARC), transcript variant 2, mRNA.	FY; Dfy; WBCQ1; CCBP1;	0.0100875	2.44421

ILMN_1656111	MUC1	1	Homo sapiens mucin 1, cell surface associated (MUC1), transcript variant 6, mRNA.	PEMT; PUM; PEM; EMA; H	0.00952959	2.44238
ILMN_1653180	NAMPT	7	Homo sapiens nicotinamide phosphoribosyltransferase (NAMPT), mRNA.	DKFZP666B131; PBEF; 1	0.00467347	2.44112
ILMN_2075189	CLEC10A	17	Homo sapiens C-type lectin domain family 10, member A (CLEC10A), transcript variant 2, mRNA.	HML; CD301; HML2; CLE	0.00365733	2.43481
ILMN_1748907	CSNK1E	22	Homo sapiens casein kinase 1, epsilon (CSNK1E), transcript variant 1, mRNA.	HCKIE; MGC10398	0.0108492	2.43418
ILMN_2355831	HCP5	6	Homo sapiens HLA complex P5 (HCP5), mRNA.	P5-1; D6S2650E	0.0342074	2.42997
ILMN_1757845	LAMC3	9	Homo sapiens laminin, gamma 3 (LAMC3), mRNA.	DKFZp434E202	0.0051931	2.42869
ILMN_1778964	SWAP70	11	Homo sapiens SWAP-70 protein (SWAP70), mRNA.	HSPC321; FLJ39540; KIA	0.00861114	2.42731
ILMN_2077952	CYBRD1	2	Homo sapiens cytochrome b reductase 1 (CYBRD1), mRNA.	FLJ23462; FRRS3; DCYTE	0.0475955	2.42456
ILMN_1792072	CD44	11	Homo sapiens CD44 molecule (Indian blood group) (CD44), transcript variant 5, mRNA.	LHR; CDW44; ECMR-III;	0.027525	2.41899
ILMN_2402600	SAMD3	6	Homo sapiens sterile alpha motif domain containing 3 (SAMD3), transcript variant 1, mRNA.	MGC35163; FLJ34563	0.048321	2.41641
ILMN_1751028	EEF1B2	2	Homo sapiens eukaryotic translation elongation factor 1 beta 2 (EEF1B2), transcript variant 2, mRNA.	EEF1B; EF1B; EEF1B1	0.00776971	2.41188

ILMN_1815346	MTHFD2	2	Homo sapiens methylenetetrahydrofolate dehydrogenase (NADP+ dependent) 2, methenyltetrahydrofolate cyclohydrolase (MTHFD2), nuclear gene encoding mitochondrial protein, transcript variant 1, mRNA.	NMDMC	0.0199047	2.41104
ILMN_1657398	ATL3	11	Homo sapiens atlastin 3 (ATL3), mRNA.	ATL3; DKFZp564J0863	0.0405896	2.41039
ILMN_1814077	SGK2	20	Homo sapiens serum/glucocorticoid regulated kinase 2 (SGK2), transcript variant 1, mRNA.	H-SGK2; dJ138B7.2	0.0195467	2.41036
ILMN_1655418			oj08g11.y1 Human lacrimal gland, unamplified: oj Homo sapiens cDNA clone oj08g11 5, mRNA sequence		0.0383652	2.40975
ILMN_1745397	PDE4B	1	Homo sapiens phosphodiesterase 4B, cAMP-specific (phosphodiesterase E4 dunce homolog, Drosophila) (PDE4B), transcript variant a, mRNA.	DKFZp686F2182; MGC12	0.0385426	2.40909
ILMN_2413508	RNASE3	14	Homo sapiens ribonuclease, RNase A family, 3 (eosinophil cationic protein) (RNASE3), mRNA.	RNS3; ECP	0.000691944	2.40653
ILMN_1680774	PDGFRB	5	Homo sapiens platelet-derived growth factor receptor, beta polypeptide (PDGFRB), mRNA.	PDGFR; JTK12; CD140B;	0.00176177	2.40394
ILMN_1678928	GYG2	X	Homo sapiens glycogenin 2 (GYG2), mRNA.	GN-2; GN2	0.00965651	2.4012
ILMN_2373831	ZNF14	19	Homo sapiens zinc finger protein 14 (ZNF14), mRNA.	GIOT-4; KOX6	0.0108226	2.40089
ILMN_1917341	VCAN	5	Homo sapiens versican (VCAN), mRNA.	DKFZp686K06110; WGN;	0.012986	2.40079

ILMN_2325347	HSD17B14	19	Homo sapiens hydroxysteroid (17-beta) dehydrogenase 14 (HSD17B14), mRNA.	DHRS10; retSDR3	0.00393008	2.40046
ILMN_2306540	PDE7A	8	Homo sapiens phosphodiesterase 7A (PDE7A), transcript variant 2, mRNA.	HCP1; PDE7	0.0141675	2.39713
ILMN_1712026	SLC2A12	6	Homo sapiens solute carrier family 2 (facilitated glucose transporter), member 12 (SLC2A12), mRNA.	GLUT12; GLUT8	0.00512111	2.39646
ILMN_2326509	SAMD3	6	Homo sapiens sterile alpha motif domain containing 3 (SAMD3), transcript variant 1, mRNA.	MGC35163; FLJ34563	0.0185066	2.39549
ILMN_1810852	UCP2	11	Homo sapiens uncoupling protein 2 (mitochondrial, proton carrier) (UCP2), nuclear gene encoding mitochondrial protein, mRNA.	SLC25A8; UCPH	0.0156855	2.39237
ILMN_1666594	STARD5	15	Homo sapiens StAR-related lipid transfer (START) domain containing 5 (STARD5), mRNA.	MGC10327	0.0426748	2.39188
ILMN_1730612	ENO2	12	Homo sapiens enolase 2 (gamma, neuronal) (ENO2), mRNA.	NSE	0.0419129	2.38898
ILMN_1803423	LRCH4	7	Homo sapiens leucine-rich repeats and calponin homology (CH) domain containing 4 (LRCH4), mRNA.	LRRN1; PP14183; SAP25;	0.0285742	2.38651
ILMN_1671149	CD248	11	Homo sapiens CD248 molecule, endosialin (CD248), mRNA.	MGC119478; CD164L1; T	0.0274709	2.38524
ILMN_1667692	TMEM126A	11	Homo sapiens transmembrane protein 126A (TMEM126A), mRNA.	DKFZp586C1924	0.0192787	2.38209
ILMN_1795429	TDO2	4	Homo sapiens tryptophan 2,3-dioxygenase (TDO2), mRNA.	TRPO; TDO; TPH2	0.00171762	2.38134

ILMN_1802646	GYPC	2	Homo sapiens glycophorin C (Gerbich blood group) (GYPC), transcript variant 2, mRNA.	MGC126191; GE; CD236F	0.0162817	2.38101
ILMN_1800425	CKMT2	5	Homo sapiens creatine kinase, mitochondrial 2 (sarcomeric) (CKMT2), nuclear gene encoding mitochondrial protein, transcript variant 1, mRNA.	SMTCK	0.0020058	2.37942
ILMN_1682929	MGC42367	2	Homo sapiens similar to 2010300C02Rik protein (MGC42367), mRNA.		0.0269208	2.37632
ILMN_2383611	FMO1	1	Homo sapiens flavin containing monooxygenase 1 (FMO1), mRNA.		0.00100163	2.37463
ILMN_1789599	CLEC5A	7	Homo sapiens C-type lectin domain family 5, member A (CLEC5A), mRNA.	MDL1; MGC138304; CLEC	0.00735065	2.37395
ILMN_1696048	CTTN	11	Homo sapiens cortactin (CTTN), transcript variant 1, mRNA.	EMS1; FLJ34459	0.0104275	2.37308
ILMN_1724181	MOCOS	18	Homo sapiens molybdenum cofactor sulfurase (MOCOS), mRNA.	MOS; FLJ20733; HMCS	0.0181139	2.36639
ILMN_1675024	VKORC1L1	7	Homo sapiens vitamin K epoxide reductase complex, subunit 1-like 1 (VKORC1L1), mRNA.	DKFZp762H0113	5.39E-05	2.3647
ILMN_2104295	QPRT	16	Homo sapiens quinolinate phosphoribosyltransferase (nicotinate-nucleotide pyrophosphorylase (carboxylating)) (QPRT), mRNA.	QPRTase	0.0291336	2.36291
ILMN_1724066	CNTNAP1	17	Homo sapiens contactin associated protein 1 (CNTNAP1), mRNA.	P190; NRXN4; CNTNAP; C	0.000832739	2.36004
ILMN_1656868	C20orf127	20	Homo sapiens chromosome 20 open reading frame 127 (C20orf127), mRNA.	dJ614O4.6; MGC118948	0.031861	2.35948

ILMN_1658847	MT1X	16	Homo sapiens metallothionein 1X (MT1X), mRNA.	MT1; MT-1l	0.0207393	2.35877
ILMN_1668592	ASB13	10	Homo sapiens ankyrin repeat and SOCS box-containing 13 (ASB13), mRNA.	FLJ13134; MGC19879	0.0340066	2.35861
ILMN_1847130	COL12A1	6	Homo sapiens collagen, type XII, alpha 1 (COL12A1), transcript variant short, mRNA.	BA209D8.1; DJ234P15.1;	0.0338814	2.35858
ILMN_1711439	KLF6	10	Homo sapiens Kruppel-like factor 6 (KLF6), transcript variant 2, mRNA.	GBF; ZF9; ST12; CPBP; B	0.00572723	2.35702
ILMN_1814998	CLU	8	Homo sapiens clusterin (CLU), transcript variant 2, mRNA.	SP-40; MGC24903; CLI; A	0.0130913	2.35675
ILMN_1760778	MGC13057	2	Homo sapiens hypothetical protein MGC13057 (MGC13057), mRNA.		0.00897793	2.35666
ILMN_1675191	ZSCAN18	19	Homo sapiens zinc finger and SCAN domain containing 18 (ZSCAN18), mRNA.	ZNF447; DKFZp586B1122	0.00442088	2.35328
ILMN_2188722	EBPL	13	Homo sapiens emopamil binding protein-like (EBPL), mRNA.	EBRP; RP11-432M24.2	0.0083961	2.35247
ILMN_1776121	SLFN11	17	Homo sapiens schlafen family member 11 (SLFN11), mRNA.	FLJ34922; SLFN8/9	0.00751504	2.34985
ILMN_1713829	HIPK2	7	Homo sapiens homeodomain interacting protein kinase 2 (HIPK2), mRNA.	PRO0593	0.0319339	2.34927
ILMN_1726392	RERG	12	Homo sapiens RAS-like, estrogen-regulated, growth inhibitor (RERG), mRNA.	MGC15754	0.00372176	2.34895
ILMN_1903914	PDE4B	1	Homo sapiens phosphodiesterase 4B, cAMP-specific (phosphodiesterase E4 dunce homolog, Drosophila) (PDE4B), transcript variant a, mRNA.	DKFZp686F2182; MGC12	0.0147984	2.34659

ILMN_1740490	GABBR1	6	Homo sapiens gamma-aminobutyric acid (GABA) B receptor, 1 (GABBR1), transcript variant 4, mRNA.	dJ271M21.1.1; dJ271M21	0.000730172	2.34123
ILMN_2175912	MEF2C	5	Homo sapiens myocyte enhancer factor 2C (MEF2C), mRNA.		0.0214134	2.33974
ILMN_1792256	NCK2	2	Homo sapiens NCK adaptor protein 2 (NCK2), transcript variant 2, mRNA.	NCKbeta; GRB4	0.0267312	2.3366
ILMN_1765446	LOC653158	5	PREDICTED: Homo sapiens similar to hypothetical protein MGC40405, transcript variant 1 (LOC653158), mRNA.		0.0080671	2.33608
ILMN_2382990	USH2A	1	Homo sapiens Usher syndrome 2A (autosomal recessive, mild) (USH2A), transcript variant 2, mRNA.	dJ1111A8.1; US2; USH2;	0.0149031	2.33382
ILMN_1680453	CSDA	12	Homo sapiens cold shock domain protein A (CSDA), mRNA.	CSDA1; DBPA; ZONAB	0.0218482	2.33102
ILMN_1738992	DHCR24	1	Homo sapiens 24-dehydrocholesterol reductase (DHCR24), mRNA.	Nbla03646; seladin-1; SE	0.0169504	2.32803
ILMN_1780465	NBL1	1	Homo sapiens neuroblastoma, suppression of tumorigenicity 1 (NBL1), transcript variant 1, mRNA.	D1S1733E; NO3; DAND1	0.00758088	2.32723
ILMN_1720124	GPAM	10	Homo sapiens glycerol-3-phosphate acyltransferase, mitochondrial (GPAM), mRNA.	GPAT1; MGC26846; KIAA	0.00489151	2.32576
ILMN_2203950	KHK	2	Homo sapiens ketohexokinase (fructokinase) (KHK), transcript variant a, mRNA.		0.00183071	2.32521
ILMN_2253732	LOC728358	8	Homo sapiens defensin, alpha 1 (LOC728358), mRNA.		0.000652375	2.32474
ILMN_1672611	GALK2	15	Homo sapiens galactokinase 2 (GALK2), transcript variant 1, mRNA.	GK2; MGC1745	0.00639486	2.32268

ILMN_1692295	P2RY2	11	Homo sapiens purinergic receptor P2Y, G-protein coupled, 2 (P2RY2), transcript variant 2, mRNA.	P2Y2R; P2Y2; P2U; MGC4	0.0101455	2.32157
ILMN_1669376	SULT1B1	4	Homo sapiens sulfotransferase family, cytosolic, 1B, member 1 (SULT1B1), mRNA.	ST1B2; MGC13356; SULT	0.00107258	2.32036
ILMN_1811636	ADFP	9	Homo sapiens adipose differentiation-related protein (ADFP), mRNA.	ADRP; MGC10598	0.00100549	2.31792
ILMN_2317923	FYB	5	Homo sapiens FYN binding protein (FYB-120/130) (FYB), transcript variant 1, mRNA.	PRO0823; SLAP-130; AD/	0.0399301	2.31552
ILMN_1685580	COL6A3	2	Homo sapiens collagen, type VI, alpha 3 (COL6A3), transcript variant 3, mRNA.	DKFZp686K04147; FLJ34	0.0197641	2.31401
ILMN_2366634	THOP1	19	Homo sapiens thimet oligopeptidase 1 (THOP1), mRNA.	MEPD_HUMAN; MP78; EP	0.00282974	2.31154
ILMN_1705258	SPARCL1	4	Homo sapiens SPARC-like 1 (mast9, hevin) (SPARCL1), mRNA.	PIG33; SC1	0.00492703	2.30777
ILMN_1831106	ZNF827	4	Homo sapiens zinc finger protein 827 (ZNF827), mRNA.		0.00168875	2.30658
ILMN_1778755	WDR54	2	Homo sapiens WD repeat domain 54 (WDR54), mRNA.	FLJ12953	0.0179644	2.3033
ILMN_1807969	ZBTB4	17	Homo sapiens zinc finger and BTB domain containing 4 (ZBTB4), mRNA.	KAISO-L1; KIAA1538	0.000968637	2.29834
ILMN_1654946	KCNK5	6	Homo sapiens potassium channel, subfamily K, member 5 (KCNK5), mRNA.	FLJ11035; K2p5.1; TASK-	0.0107646	2.29731
ILMN_1799725			602572519F1 NIH_MGC_77 Homo sapiens cDNA clone IMAGE:4700548 5, mRNA sequence		0.0376568	2.29671
ILMN_2307032	PIK3IP1	22	Homo sapiens phosphoinositide-3-kinase interacting protein 1 (PIK3IP1), mRNA.	HGFL; hHGFL(S); MGC17	0.0264015	2.29669

ILMN_1759628	TMEM43	3	Homo sapiens transmembrane protein 43 (TMEM43), mRNA.	DKFZp586G1919; MGC32	0.016604	2.29595
ILMN_1732967	CFI	4	Homo sapiens complement factor I (CFI), mRNA.	IF; KAF; FI; factor I; C3b	0.0119427	2.29554
ILMN_1689251	ANTXR1	2	Homo sapiens anthrax toxin receptor 1 (ANTXR1), transcript variant 1, mRNA.	ATR; FLJ21776; FLJ11298	0.00056275	2.295
ILMN_2195482	NGEF	2	Homo sapiens neuronal guanine nucleotide exchange factor (NGEF), mRNA.	EPHEXIN	0.00422767	2.29497
ILMN_1654396	ABCC9	12	Homo sapiens ATP-binding cassette, sub-family C (CFTR/MRP), member 9 (ABCC9), transcript variant SUR2A-delta-14, mRNA.	ABC37; FLJ36852; SUR2;	0.0370543	2.29493
ILMN_1680070	CTTN	11	Homo sapiens cortactin (CTTN), transcript variant 2, mRNA.	EMS1; FLJ34459	0.024683	2.2949
ILMN_1760027	SYTL2	11	Homo sapiens synaptotagmin-like 2 (SYTL2), transcript variant e, mRNA.	MGC102768; CHR11SYT;	0.0469287	2.29218
ILMN_2057981	LIPC	15	Homo sapiens lipase, hepatic (LIPC), mRNA.	LIPH; HL; HTGL	0.00126934	2.29059
ILMN_1753112	ID3	1	Homo sapiens inhibitor of DNA binding 3, dominant negative helix-loop-helix protein (ID3), mRNA.	HEIR-1	0.00977273	2.28926
ILMN_1658677	GPHN	14	Homo sapiens gephyrin (GPHN), transcript variant 2, mRNA.	GPHRYN; GPH; GEPH; KIA	0.0170707	2.28602
ILMN_1682818	OSBPL3	7	Homo sapiens oxysterol binding protein-like 3 (OSBPL3), transcript variant 4, mRNA.	MGC21526; DKFZp667P1	0.0253895	2.28409
ILMN_2351638	LAMP3	3	Homo sapiens lysosomal-associated membrane protein 3 (LAMP3), mRNA.	TSC403; DC-LAMP; CD20	0.0366684	2.28168

ILMN_1672878	CHCHD8	11	Homo sapiens coiled-coil-helix-coiled-coil-helix domain containing 8 (CHCHD8), mRNA.	DKFZp762H1711; E2IG2;	0.0087062	2.27906
ILMN_1684401	LOC642755	15	PREDICTED: Homo sapiens similar to DEXI, transcript variant 1 (LOC642755), mRNA.		0.0391681	2.27754
ILMN_1710092	ITPR3	6	Homo sapiens inositol 1,4,5-triphosphate receptor, type 3 (ITPR3), mRNA.	FLJ36205; IP3R; IP3R3	0.00085123	2.27552
ILMN_1681886	HRG	3	Homo sapiens histidine-rich glycoprotein (HRG), mRNA.	HRGP; DKFZp779H1622;	0.00046123	2.27071
ILMN_1694213	LOXL4	10	Homo sapiens lysyl oxidase-like 4 (LOXL4), mRNA.	FLJ21889; LOXC	0.00279443	2.26645
ILMN_1738773	ITM2C	2	Homo sapiens integral membrane protein 2C (ITM2C), transcript variant 2, mRNA.	E25; ITM3; BRI3; E25C; I	0.0138005	2.26508
ILMN_1657554	DEXI	16	Homo sapiens dexamethasone-induced transcript (DEXI), mRNA.	MYLE	0.0100067	2.26346
ILMN_2095273	LOC653600	8	PREDICTED: Homo sapiens similar to Neutrophil defensin 1 precursor (HNP-1) (HP-1) (HP1) (Defensin, alpha 1) (LOC653600), mRNA.		0.0011609	2.26279
ILMN_2116299	SMOC2	6	Homo sapiens SPARC related modular calcium binding 2 (SMOC2), mRNA.	dJ421D16.1; MSTP140; b	0.0218412	2.2627
ILMN_1698934	FBLN1	22	Homo sapiens fibulin 1 (FBLN1), transcript variant A, mRNA.	FBLN	0.0475103	2.2612
ILMN_1659490	CENTD3	5	Homo sapiens centaurin, delta 3 (CENTD3), mRNA.	FLJ21065; ARAP3; DRAG1	0.0123584	2.26009
ILMN_1765796	ZSWIM4	19	Homo sapiens zinc finger, SWIM-type containing 4 (ZSWIM4), mRNA.		0.00303645	2.25632
ILMN_1810486	RPL29	3	Homo sapiens ribosomal protein L29 (RPL29), mRNA.	HUMRPL29; MGC88589; H	0.00092964	2.25547

ILMN_1727577	VCL	10	Homo sapiens vinculin (VCL), transcript variant 1, mRNA.	MVCL	0.000761061	2.2506
ILMN_1741200	CRP	1	Homo sapiens C-reactive protein, pentraxin-related (CRP), mRNA.	MGC149895; PTX1; MGC8	5.86E-05	2.2459
ILMN_2410145	STAT1	2	Homo sapiens signal transducer and activator of transcription 1, 91kDa (STAT1), transcript variant beta, mRNA.	ISGF-3; STAT91; DKFZp6	0.0181059	2.24539
ILMN_1689378	TPM2	9	Homo sapiens tropomyosin 2 (beta) (TPM2), transcript variant 2, mRNA.	AMCD1; TMSB; DA1	0.0460804	2.2453
ILMN_1803367	TBC1D16	17	Homo sapiens TBC1 domain family, member 16 (TBC1D16), mRNA.	FLJ20748; MGC25062	0.0132404	2.24291
ILMN_2381197	TSPAN13	7	Homo sapiens tetraspanin 13 (TSPAN13), mRNA.	FLJ22934; NET-6; TM4SF	0.00849826	2.24242
ILMN_1742981	HBG1	11	Homo sapiens hemoglobin, gamma A (HBG1), mRNA.	HBGR; HSGGL1; HBGA; F	0.0155566	2.24236
ILMN_1764729	FRMD6	14	Homo sapiens FERM domain containing 6 (FRMD6), mRNA.	c14_5320; MGC17921; C	0.0373737	2.24088
ILMN_1742332	MXRA7	17	Homo sapiens matrix-remodelling associated 7 (MXRA7), transcript variant 1, mRNA.	FLJ46603; TMAP1; PS1TP	0.0152472	2.23933
ILMN_1694426	DHODH	16	Homo sapiens dihydroorotate dehydrogenase (DHODH), nuclear gene encoding mitochondrial protein, mRNA.	DHOdehase	0.000145601	2.23888
ILMN_1788053	PLEKHO1	1	Homo sapiens pleckstrin homology domain containing, family O member 1 (PLEKHO1), mRNA.	CKIP-1; OC120; RP11-45	0.00129529	2.23691
ILMN_1661875	ZHX3	20	Homo sapiens zinc fingers and homeoboxes 3 (ZHX3), mRNA.	KIAA0395; TIX1	0.0473921	2.23125
ILMN_1705231	ADCY3	2	Homo sapiens adenylate cyclase 3 (ADCY3), mRNA.	KIAA0511; AC3	0.0235138	2.22966

ILMN_1677432	C19orf59	19	Homo sapiens chromosome 19 open reading frame 59 (C19orf59), mRNA.	MGC132456; MCEMP1	0.00280076	2.2295
ILMN_1855278	C7orf23	7	Homo sapiens chromosome 7 open reading frame 23 (C7orf23), mRNA.	MM-TRAG; MGC4175	0.0481675	2.22871
ILMN_1715748	C8orf70	8	Homo sapiens chromosome 8 open reading frame 70 (C8orf70), mRNA.	CGI-62	0.00332512	2.22646
ILMN_2393712	CDKN2AIPN	5	Homo sapiens CDKN2A interacting protein N-terminal like (CDKN2AIPNL), mRNA.	MGC13017	0.00348373	2.2263
ILMN_1811890	TMEM156	4	Homo sapiens transmembrane protein 156 (TMEM156), mRNA.	FLJ23235	0.0278262	2.22333
ILMN_1691539	ABAT	16	Homo sapiens 4-aminobutyrate aminotransferase (ABAT), nuclear gene encoding mitochondrial protein, transcript variant 1, mRNA.	NPD009; GABAT; GABA-A	0.0369793	2.22028
ILMN_1729987	DRAM	12	Homo sapiens damage-regulated autophagy modulator (DRAM), mRNA.	FLJ11259	0.0193039	2.21821
ILMN_1801928	NUDT2	9	Homo sapiens nudix (nucleoside diphosphate linked moiety X)-type motif 2 (NUDT2), transcript variant 3, mRNA.	APAH1; MGC10404	0.00759779	2.21537
ILMN_1815745	OLR1	12	Homo sapiens oxidized low density lipoprotein (lectin-like) receptor 1 (OLR1), mRNA.	SCARE1; LOX1; CLEC8A	0.00984225	2.21279
ILMN_1763704	TMEM119	12	Homo sapiens transmembrane protein 119 (TMEM119), mRNA.		0.0303565	2.21231

ILMN_1796629			BX118953 Soares_pregnant_uterus_NbHPU Homo sapiens cDNA clone IMAGp998K024347, mRNA sequence		0.00558734	2.21055
ILMN_2161357	GSTM4	1	Homo sapiens glutathione S-transferase M4 (GSTM4), transcript variant 1, mRNA.	GSTM4-4; MGC131945; M	0.0271133	2.20675
ILMN_1709348	EGFLAM	5	Homo sapiens EGF-like, fibronectin type III and laminin G domains (EGFLAM), transcript variant 4, mRNA.	AGRINL; AGRNL; FLJ3915	0.0193378	2.20628
ILMN_1751079	RCC2	1	Homo sapiens regulator of chromosome condensation 2 (RCC2), mRNA.	DKFZp762N0610; KIAA14	0.0347433	2.20363
ILMN_2059452	NCF1	7	Homo sapiens neutrophil cytosolic factor 1, (chronic granulomatous disease, autosomal 1) (NCF1), mRNA.	NOXO2; SH3PXD1A; NCF	0.00887014	2.20174
ILMN_1770824	EMILIN2	18	Homo sapiens elastin microfibril interfacer 2 (EMILIN2), mRNA.	FLJ33200; FOAP-10; EMI	0.00930304	2.20124
ILMN_1804277	CPN2	3	Homo sapiens carboxypeptidase N, polypeptide 2 (CPN2), mRNA.	ACBP	0.0290881	2.20046
ILMN_1797009	HSPA2	14	Homo sapiens heat shock 70kDa protein 2 (HSPA2), mRNA.		0.0344169	2.19697
ILMN_1718046	PTPRE	10	Homo sapiens protein tyrosine phosphatase, receptor type, E (PTPRE), transcript variant 2, mRNA.	DKFZp313F1310; PTPE; H	0.0156872	2.18803
ILMN_1749078	JAG1	20	Homo sapiens jagged 1 (Alagille syndrome) (JAG1), mRNA.	CD339; AWS; HJ1; JAGL1	0.0241429	2.18737
ILMN_1677723	MGP	12	Homo sapiens matrix Gla protein (MGP), mRNA.	NTI; GIG36; MGLAP	0.00270838	2.18727

ILMN_1742789	ITM2C	2	Homo sapiens integral membrane protein 2C (ITM2C), transcript variant 2, mRNA.	E25; ITM3; BRI3; E25C; I	0.0470026	2.18688
ILMN_1743933	LBH	2	Homo sapiens limb bud and heart development homolog (mouse) (LBH), mRNA.	MGC163287; MGC104312	0.0141094	2.1825
ILMN_1729217	GFRA1	10	Homo sapiens GDNF family receptor alpha 1 (GFRA1), transcript variant 1, mRNA.	MGC23045; RET1L; TRNR	0.0169591	2.1819
ILMN_1706015	COL1A2	7	Homo sapiens collagen, type I, alpha 2 (COL1A2), mRNA.	OI4	0.0122191	2.18165
ILMN_1702301	SYT11	1	Homo sapiens synaptotagmin XI (SYT11), mRNA.	MGC17226; DKFZp781D0	0.0438955	2.17826
ILMN_1789364	UGCG	9	Homo sapiens UDP-glucose ceramide glucosyltransferase (UGCG), mRNA.	GCS	0.0304713	2.17826
ILMN_2361862	CD300LG	17	Homo sapiens CD300 molecule-like family member g (CD300LG), mRNA.	CLM9; TREM4	0.000257406	2.17756
ILMN_1812559	ACACA	17	Homo sapiens acetyl-Coenzyme A carboxylase alpha (ACACA), transcript variant 3, mRNA.	ACC; ACC1; ACAC; ACCA	0.0126641	2.17571
ILMN_1674380	FCN2	9	Homo sapiens ficolin (collagen/fibrinogen domain containing lectin) 2 (hucolin) (FCN2), transcript variant SV1, mRNA.	EBP-37; FCNL; ficolin-2; I	0.0215165	2.17257
ILMN_1659371			UI-1-BC1p-aky-f-10-0-UI.s1 NCI_CGAP_Pl3 Homo sapiens cDNA clone UI-1-BC1p-aky-f-10-0-UI 3, mRNA sequence		0.0232676	2.1639
ILMN_1758825	NBL1	1	Homo sapiens neuroblastoma, suppression of tumorigenicity 1 (NBL1), transcript variant 2, mRNA.	D1S1733E; NB; NO3; DA	0.020094	2.16358

ILMN_1713499	CACNB3	12	Homo sapiens calcium channel, voltage-dependent, beta 3 subunit (CACNB3), mRNA.	CACNLB3	0.0348385	2.15994
ILMN_2094776	PRO0628	20	Homo sapiens PRO0628 protein (PRO0628) on chromosome 20.		0.0323215	2.1571
ILMN_1698605	FLRT2	14	Homo sapiens fibronectin leucine rich transmembrane protein 2 (FLRT2), mRNA.	KIAA0405	0.00770136	2.15558
ILMN_1778321	HBD	11	Homo sapiens hemoglobin, delta (HBD), mRNA.		0.0231288	2.15507
ILMN_1751143	BIRC3	11	Homo sapiens baculoviral IAP repeat-containing 3 (BIRC3), transcript variant 2, mRNA.	RNF49; MALT2; API2; HIA	0.031214	2.15433
ILMN_1733756			Homo sapiens cDNA FLJ32401 fis, clone SKMUS2000339		0.0252298	2.15256
ILMN_1785170	CCDC3	10	Homo sapiens coiled-coil domain containing 3 (CCDC3), mRNA.	FLJ20925; DKFZP761F24	0.0208191	2.15212
ILMN_1791569	SYTL2	11	Homo sapiens synaptotagmin-like 2 (SYTL2), transcript variant d, mRNA.	MGC102768; CHR11SYT;	0.00948592	2.15169
ILMN_1699574	GPR37	7	Homo sapiens G protein-coupled receptor 37 (endothelin receptor type B-like) (GPR37), mRNA.	hET(B)R-LP; EDNRBL; PA	0.0148028	2.15115
ILMN_1677511	RALYL	8	Homo sapiens RALY RNA binding protein-like (RALYL), transcript variant 3, mRNA.	HNRPCL3	0.0374307	2.15073
ILMN_1807003	THRSP	11	Homo sapiens thyroid hormone responsive (SPOT14 homolog, rat) (THRSP), mRNA.	MGC21659; S14; SPOT14	0.0112811	2.14884
ILMN_1724753	TFF3	21	Homo sapiens trefoil factor 3 (intestinal) (TFF3), mRNA.	TFI; hP1.B; HITF; ITF	0.000844158	2.14849
ILMN_1911042	MAD1L1	7	Homo sapiens MAD1 mitotic arrest deficient-like 1 (yeast) (MAD1L1), transcript variant 2, mRNA.	PIG9; HsMAD1; TXBP181	0.0192663	2.14727

ILMN_1784608	JAG2	14	Homo sapiens jagged 2 (JAG2), transcript variant 1, mRNA.	HJ2	0.0176016	2.14617
ILMN_1685625			full-length cDNA clone XCL0BB001ZD04 of Neuroblastoma of Homo sapiens (human)		0.0100555	2.14412
ILMN_2097793	STK39	2	Homo sapiens serine threonine kinase 39 (STE20/SPS1 homolog, yeast) (STK39), mRNA.	DCHT; PASK; SPAK; DKF	0.0111041	2.1427
ILMN_1699208	CCL2	17	Homo sapiens chemokine (C-C motif) ligand 2 (CCL2), mRNA.	SMC-CF; MCP1; MCAF; H	0.00667662	2.14199
ILMN_1743836	GAS1	9	Homo sapiens growth arrest-specific 1 (GAS1), mRNA.		0.00553338	2.14144
ILMN_2331231	DIDO1	20	Homo sapiens death inducer-obliterator 1 (DIDO1), transcript variant 3, mRNA.	dJ885L7.8; DIDO3; DATF	0.0175381	2.14093
ILMN_1659544	STOM	9	Homo sapiens stomatin (STOM), transcript variant 1, mRNA.	EPB7; EPB72; BND7	0.00211031	2.13947
ILMN_1652246	C21orf41	21	Homo sapiens chromosome 21 open reading frame 41 (C21orf41) on chromosome 21.		0.0286606	2.13553
ILMN_1653166	PID1	2	Homo sapiens phosphotyrosine interaction domain containing 1 (PID1), mRNA.	HMFN2073; FLJ20701; N	0.0454781	2.13551
ILMN_2103547	C7	5	Homo sapiens complement component 7 (C7), mRNA.		0.0308929	2.1344
ILMN_1795118	ELA2	19	Homo sapiens elastase 2, neutrophil (ELA2), mRNA.	HLE; GE; PMN-E; NE; HN	0.00708129	2.13408
ILMN_1716488	MTM	16	Homo sapiens metallothionein M (MTM), mRNA.		0.0242461	2.13043
ILMN_2318638	BTN3A2	6	Homo sapiens butyrophilin, subfamily 3, member A2 (BTN3A2), mRNA.	BT3.3; BT3.2; BTF4	0.00257961	2.12969

ILMN_1697309	FCN3	1	Homo sapiens ficolin (collagen/fibrinogen domain containing) 3 (Hakata antigen) (FCN3), transcript variant 2, mRNA.	MGC22543; FCNH; HAKA	0.0163677	2.12774
ILMN_1765459	CD97	19	Homo sapiens CD97 molecule (CD97), transcript variant 1, mRNA.	TM7LN1	0.0139021	2.12625
ILMN_2390859	GSTM1	1	Homo sapiens glutathione S-transferase M1 (GSTM1), transcript variant 1, mRNA.	MGC26563; GSTM1-1; H-	0.0376445	2.12488
ILMN_1661491	MRPL22	5	Homo sapiens mitochondrial ribosomal protein L22 (MRPL22), nuclear gene encoding mitochondrial protein, transcript variant 1, mRNA.	HSPC158; DKFZp781F10	0.0151015	2.1242
ILMN_1697268	LIPG	18	Homo sapiens lipase, endothelial (LIPG), mRNA.	EL; PRO719; EDL	0.0285904	2.1241
ILMN_1721833	PDE9A	21	Homo sapiens phosphodiesterase 9A (PDE9A), transcript variant 2, mRNA.	HSPDE9A2	0.0494721	2.11965
ILMN_1806403	HPGD	4	Homo sapiens hydroxyprostaglandin dehydrogenase 15-(NAD) (HPGD), mRNA.	PGDH; 15-PGDH; PGDH1	0.0392182	2.11897
ILMN_1763605	VWF	12	Homo sapiens von Willebrand factor (VWF), mRNA.	F8VWF; VWD	0.0221028	2.11888
ILMN_1704730	AZGP1	7	Homo sapiens alpha-2-glycoprotein 1, zinc-binding (AZGP1), mRNA.	ZAG; ZA2G	0.00395194	2.11776
ILMN_1663648	SH2B3	12	Homo sapiens SH2B adaptor protein 3 (SH2B3), mRNA.	LNK	0.0123267	2.11365
ILMN_2203271	TMEM17	2	Homo sapiens transmembrane protein 17 (TMEM17), mRNA.	FLJ34583	0.0191953	2.11295
ILMN_1708375	MASP2	1	Homo sapiens mannan-binding lectin serine peptidase 2 (MASP2), transcript variant 1, mRNA.	sMAP; MASP-2; MAP19	0.0481159	2.11167

ILMN_1678143	EFEMP2	11	Homo sapiens EGF-containing fibulin-like extracellular matrix protein 2 (EFEMP2), mRNA.	UPH1; FBLN4; MBP1	0.024711	2.1077
ILMN_1659158	KCNMA1	10	Homo sapiens potassium large conductance calcium-activated channel, subfamily M, alpha member 1 (KCNMA1), transcript variant 2, mRNA.	MaxiK; SLO-ALPHA; MGC	0.0426955	2.10767
ILMN_2396272	LPXN	11	Homo sapiens leupaxin (LPXN), mRNA.	LDPL	0.0178395	2.10367
ILMN_2328378	SP110	2	Homo sapiens SP110 nuclear body protein (SP110), transcript variant b, mRNA.	FLJ22835; IFI75; VODI; I	0.000710822	2.10004
ILMN_1731358	SH2D3C	9	Homo sapiens SH2 domain containing 3C (SH2D3C), transcript variant 2, mRNA.	NSP3; PRO34088; CHAT;	0.000964412	2.09846
ILMN_2319000	SLC13A5	17	Homo sapiens solute carrier family 13 (sodium-dependent citrate transporter), member 5 (SLC13A5), mRNA.	MGC138356; NACT; DKF	0.0266217	2.09586
ILMN_2318643	C6orf129	6	Homo sapiens chromosome 6 open reading frame 129 (C6orf129), mRNA.	HSPC265; MGC131656	0.0181133	2.09459
ILMN_1703477	PNKD	2	Homo sapiens paroxysmal nonkinesigenic dyskinesia (PNKD), transcript variant 2, mRNA.	FKSG19; KIPP1184; MR1	0.00395106	2.0939
ILMN_1658289	LOC653498	17	PREDICTED: Homo sapiens similar to TBC1 domain family, member 3B (LOC653498), mRNA.		0.0402943	2.09168
ILMN_1790014	MMAB	12	Homo sapiens methylmalonic aciduria (cobalamin deficiency) cblB type (MMAB), mRNA.	ATR; MGC20496	0.0251402	2.09129
ILMN_1802151	CD86	3	Homo sapiens CD86 molecule (CD86), transcript variant 2, mRNA.	B7-2; B70; LAB72; MGC3	0.0131645	2.09111

ILMN_1703273	PLAUR	19	Homo sapiens plasminogen activator, urokinase receptor (PLAUR), transcript variant 2, mRNA.	CD87; UPAR; URKR	0.000139908	2.09063
ILMN_1768595	EBP	X	Homo sapiens emopamil binding protein (sterol isomerase) (EBP), mRNA.	CDPX2; CPXD; CHO2; CP	0.000292829	2.0904
ILMN_1730625	TAT	16	Homo sapiens tyrosine aminotransferase (TAT), nuclear gene encoding mitochondrial protein, mRNA.		0.0116018	2.09018
ILMN_1846001	SDCBP2	20	Homo sapiens syndecan binding protein (syntenin) 2 (SDCBP2), transcript variant 2, mRNA.	SITAC18; FLJ12256; ST-2	0.00245188	2.09008
ILMN_2358069	LBP	20	Homo sapiens lipopolysaccharide binding protein (LBP), mRNA.	MGC22233	0.0460206	2.08984
ILMN_1787378	ZBTB16	11	Homo sapiens zinc finger and BTB domain containing 16 (ZBTB16), transcript variant 1, mRNA.	PLZF; ZNF145	0.0239864	2.08929
ILMN_1767377	FHL2	2	Homo sapiens four and a half LIM domains 2 (FHL2), transcript variant 2, mRNA.	SLIM3; AAG11; DRAL	0.0276484	2.08866
ILMN_1737833	IL1R2	2	Homo sapiens interleukin 1 receptor, type II (IL1R2), transcript variant 2, mRNA.	IL1RB; CD121b; MGC477	0.012236	2.08773
ILMN_1775734	GPR124	8	Homo sapiens G protein-coupled receptor 124 (GPR124), mRNA.	DKFZp434C211; TEM5; D	0.0121755	2.08726
ILMN_1671046	GLI2	2	Homo sapiens GLI-Kruppel family member GLI2 (GLI2), mRNA.	HPE9; THP2	0.00984793	2.08553
ILMN_2330787	ZNF462	9	Homo sapiens zinc finger protein 462 (ZNF462), mRNA.	FLJ45904; RP11-508N12.	0.0205079	2.08416
ILMN_1762594	KIAA1324L	7	Homo sapiens KIAA1324-like (KIAA1324L), mRNA.	FLJ31340	0.00692614	2.08274
ILMN_1792404	HYOU1	11	Homo sapiens hypoxia up-regulated 1 (HYOU1), mRNA.	DKFZp686N08236; ORP1	0.0105485	2.08164

ILMN_1652065	MT1M	16	Homo sapiens metallothionein 1M (MT1M), mRNA.	MT1; MGC118949; MT1K	0.00134798	2.08126
ILMN_1795835	MOXD1	6	Homo sapiens monooxygenase, DBH-like 1 (MOXD1), transcript variant 2, mRNA.	MOX; dJ248E1.1; PRO578	0.0216607	2.08057
ILMN_1718607	LRRC49	15	Homo sapiens leucine rich repeat containing 49 (LRRC49), mRNA.	FLJ20156	0.00524803	2.08048
ILMN_1726809	HBG2	11	Homo sapiens hemoglobin, gamma G (HBG2), mRNA.		0.0175117	2.08031
ILMN_1785175	FER1L4	20	Homo sapiens fer-1-like 4 (C. elegans) (FER1L4) on chromosome 20.	dJ309K20.1; FLJ22613; b	0.00530721	2.07915
ILMN_1691942	NR1I2	3	Homo sapiens nuclear receptor subfamily 1, group I, member 2 (NR1I2), transcript variant 1, mRNA.	PAR; PRR; SAR; PAR1; O	0.0151734	2.07799
ILMN_1675523	PCBD1	10	Homo sapiens 6-pyruvoyl-tetrahydropterin synthase/dimerization cofactor of hepatocyte nuclear factor 1 alpha (TCF1) (PCBD1), transcript variant 2, mRNA.	PHS; PCD; DCOH; PCBD	0.0368296	2.0779
ILMN_1752046	LSS	21	Homo sapiens lanosterol synthase (2,3-oxidosqualene-lanosterol cyclase) (LSS), transcript variant 1, mRNA.	OSC	0.000165759	2.07574
ILMN_2235137	LOC338758	12	PREDICTED: Homo sapiens hypothetical protein LOC338758 (LOC338758), mRNA.		0.0071549	2.07233
ILMN_1798663	PALLD	4	Homo sapiens palladin, cytoskeletal associated protein (PALLD), mRNA.	CGI-151; PNCA1; KIAA09	0.0222599	2.07133
ILMN_2068257	ARHGEF6	X	Homo sapiens Rac/Cdc42 guanine nucleotide exchange factor (GEF) 6 (ARHGEF6), mRNA.	COOL2; PIXA; MRX46; KI	0.0136862	2.06987

ILMN_1682799	SLC44A1	9	Homo sapiens solute carrier family 44, member 1 (SLC44A1), mRNA.	RP11-287A8.1; CHTL1; C	0.0137977	2.06922
ILMN_1714296	KIAA1641		PREDICTED: Homo sapiens KIAA1641, transcript variant 7 (KIAA1641), mRNA.		0.0211211	2.06859
ILMN_2130078	ECE2	3	Homo sapiens endothelin converting enzyme 2 (ECE2), transcript variant 1, mRNA.	MGC2408; KIAA0604	0.0345077	2.06563
ILMN_2233366	P2RY8	Un|NW_001841157.1	Homo sapiens purinergic receptor P2Y, G-protein coupled, 8 (P2RY8), mRNA.	MGC50878; P2Y8	0.000310018	2.06527
ILMN_1694589	PCSK6	15	Homo sapiens proprotein convertase subtilisin/kexin type 6 (PCSK6), transcript variant 6, mRNA.	PACE4; SPC4	0.00059495	2.06417
ILMN_1731418	PPP2R1B	11	Homo sapiens protein phosphatase 2 (formerly 2A), regulatory subunit A, beta isoform (PPP2R1B), transcript variant 2, mRNA.	MGC26454; PR65B	0.0114281	2.06234
ILMN_2059549	PC	11	Homo sapiens pyruvate carboxylase (PC), nuclear gene encoding mitochondrial protein, transcript variant 2, mRNA.	PCB	0.0151343	2.06085
ILMN_2317751	LOC647450		PREDICTED: Homo sapiens similar to Ig kappa chain V-I region HK101 precursor (LOC647450), mRNA.		0.0167757	2.05954
ILMN_1803945	BIRC3	11	Homo sapiens baculoviral IAP repeat-containing 3 (BIRC3), transcript variant 1, mRNA.	RNF49; MALT2; MIHC; HA	0.0404014	2.05931
ILMN_1714602	HAX1	1	Homo sapiens HCLS1 associated protein X-1 (HAX1), transcript variant 1, mRNA.	HCLSBP1; SCN3; HS1BP1	0.0403148	2.05867
ILMN_1768311	STC2	5	Homo sapiens stanniocalcin 2 (STC2), mRNA.	STCRP; STC-2	0.0339275	2.05564

ILMN_2340027	FCN1	9	Homo sapiens ficolin (collagen/fibrinogen domain containing) 1 (FCN1), mRNA.	FCNM	0.0140761	2.05478
ILMN_1669390	FANCC	9	Homo sapiens Fanconi anemia, complementation group C (FANCC), mRNA.	FLJ14675; FA3; FACC; FA	0.0326355	2.05386
ILMN_1774077	VANGL2	1	Homo sapiens vang-like 2 (van gogh, Drosophila) (VANGL2), mRNA.	MGC119404; LTAP; MGC1	0.0359089	2.05209
ILMN_1745778	CCND2	12	Homo sapiens cyclin D2 (CCND2), mRNA.	KIAK0002; MGC102758	0.0402852	2.05102
ILMN_1771599	TMEM37	2	Homo sapiens transmembrane protein 37 (TMEM37), mRNA.	PR1; PR; CACNG5	0.00142863	2.04753
ILMN_1744912	ABCB4	7	Homo sapiens ATP-binding cassette, sub-family B (MDR/TAP), member 4 (ABCB4), transcript variant C, mRNA.	PGY3; PFIC-3; MDR2/3; M	0.00602958	2.04601
ILMN_1894072	SLC12A2	5	Homo sapiens solute carrier family 12 (sodium/potassium/chloride transporters), member 2 (SLC12A2), mRNA.	NKCC1; BSC2; MGC1042	0.0112535	2.04237
ILMN_2210386	GLYAT	11	Homo sapiens glycine-N-acyltransferase (GLYAT), nuclear gene encoding mitochondrial protein, transcript variant 1, mRNA.	ACGNAT; CAT; GAT	0.00968689	2.04232
ILMN_1660727			AGENCOURT_8122036 Lupski_dorsal_root_ganglion Homo sapiens cDNA clone IMAGE:6179261 5, mRNA sequence		0.00984507	2.04229
ILMN_2408576	OAS3	12	Homo sapiens 2'-5'-oligoadenylate synthetase 3, 100kDa (OAS3), mRNA.	MGC133260; p100	0.0182049	2.04074
ILMN_1668374	NTN4	12	Homo sapiens netrin 4 (NTN4), mRNA.	PRO3091; FLJ23180	0.0324203	2.04067

ILMN_1718734	MPDU1	17	Homo sapiens mannose-P-dolichol utilization defect 1 (MPDU1), mRNA.	CDGIF; HBEBP2BPA; FLJ1	0.0300331	2.04057
ILMN_1786429	SOX4	6	Homo sapiens SRY (sex determining region Y)-box 4 (SOX4), mRNA.	EVI16	0.0418488	2.04045
ILMN_2374687	ADD3	10	Homo sapiens adducin 3 (gamma) (ADD3), transcript variant 1, mRNA.	ADDL	0.0294593	2.03773
ILMN_1696843	KCNMB1	5	Homo sapiens potassium large conductance calcium-activated channel, subfamily M, beta member 1 (KCNMB1), mRNA.	K(VCA)beta; SLO-BETA; [illegible]	0.0244318	2.03609
ILMN_1717046	PAPLN	14	Homo sapiens papilin, proteoglycan-like sulfated glycoprotein (PAPLN), mRNA.	DKFZp434F053; MGC504	0.00163891	2.0345
ILMN_1799026	SPINK1	5	Homo sapiens serine peptidase inhibitor, Kazal type 1 (SPINK1), mRNA.	Spink3; PCTT; PSTI; TAT[illegible]	0.0224878	2.03397
ILMN_1777721	STEAP3	2	Homo sapiens STEAP family member 3 (STEAP3), transcript variant 1, mRNA.	STMP3; dudlin-2; TSAP6	0.00183293	2.03395
ILMN_1692145	ACMSD	2	Homo sapiens aminocarboxymuconate semialdehyde decarboxylase (ACMSD), mRNA.		0.0297658	2.03334
ILMN_1815121	NPC1L1	7	Homo sapiens NPC1 (Niemann-Pick disease, type C1, gene)-like 1 (NPC1L1), mRNA.		0.00210376	2.03103
ILMN_1666078	ARHGAP23	17	PREDICTED: Homo sapiens Rho GTPase activating protein 23, transcript variant 1 (ARHGAP23), mRNA.		0.0483363	2.03095
ILMN_1754179	LHFPL3	7	Homo sapiens lipoma HMGIC fusion partner-like 3 (LHFPL3), mRNA.	LHFPL4	0.010345	2.03064
ILMN_1779031	S100A11	1	Homo sapiens S100 calcium binding protein A11 (S100A11), mRNA.	S100C; MLN70	0.0332373	2.03048

ILMN_1695959	PODXL	7	Homo sapiens podocalyxin-like (PODXL), transcript variant 1, mRNA.	MGC138240; PCLP; Gp20	0.0464917	2.02975
ILMN_1727689	TADA1L	1	Homo sapiens transcriptional adaptor 1 (HFI1 homolog, yeast)-like (TADA1L), mRNA.	RP1-9E21.4; KIAA0764; S	0.00751697	2.02887
ILMN_1756071	PCSK6	15	Homo sapiens proprotein convertase subtilisin/kexin type 6 (PCSK6), transcript variant 7, mRNA.	PACE4; SPC4	0.0399408	2.02816
ILMN_1665040	LYVE1	11	Homo sapiens lymphatic vessel endothelial hyaluronan receptor 1 (LYVE1), mRNA.	CRSBP-1; XLKD1; LYVE-1	0.0412418	2.02806
ILMN_2062714	PTGS2	1	Homo sapiens prostaglandin-endoperoxide synthase 2 (prostaglandin G/H synthase and cyclooxygenase) (PTGS2), mRNA.	PHS-2; COX-2; hCox-2; P	0.0318892	2.01992
ILMN_2213834	RALGPS1	9	Homo sapiens Ral GEF with PH domain and SH3 binding motif 1 (RALGPS1), mRNA.	KIAA0351; RALGPS1A; RI	0.0340917	2.01698
ILMN_2380494	HCG4	6	Homo sapiens HLA complex group 4 (HCG4) on chromosome 6.	HCGIV.9	0.000631986	2.01582
ILMN_2415748	LOC654174		PREDICTED: Homo sapiens similar to lethal (2) k00619 CG4775-PA (LOC654174), mRNA.		0.0225725	2.01575
ILMN_1692398	CAV1	7	Homo sapiens caveolin 1, caveolae protein, 22kDa (CAV1), mRNA.	CAV; VIP21; MSTP085	0.00730033	2.01482
ILMN_1693766	S100A8	1	Homo sapiens S100 calcium binding protein A8 (S100A8), mRNA.	CFAG; 60B8AG; MRP8; C(	0.0177728	2.01381

ILMN_1744212	SEMA4B	15	Homo sapiens sema domain, immunoglobulin domain (Ig), transmembrane domain (TM) and short cytoplasmic domain, (semaphorin) 4B (SEMA4B), transcript variant 2, mRNA.	KIAA1745; SemC; SEMAC	0.00291036	2.01306
ILMN_2415144	LOC651898		PREDICTED: Homo sapiens similar to Ig kappa chain V-II region Cum (LOC651898), mRNA.		0.0151825	2.01289
ILMN_1697491	SRPRB	3	Homo sapiens signal recognition particle receptor, B subunit (SRPRB), mRNA.	APMCF1	0.0062727	2.01288
ILMN_1726981	ZNF545	19	Homo sapiens zinc finger protein 545 (ZNF545), mRNA.	MGC45380; KIAA1948	0.0129472	2.0128
ILMN_1727183	VSIG4	X	Homo sapiens V-set and immunoglobulin domain containing 4 (VSIG4), transcript variant 1, mRNA.	CRIg; Z39IG	0.0185799	2.01146
ILMN_1674135	CTSK	1	Homo sapiens cathepsin K (CTSK), mRNA.	CTSO2; CTSO; CTSO1; PI	0.039881	2.01065
ILMN_1682783	HARS	5	Homo sapiens histidyl-tRNA synthetase (HARS), mRNA.	FLJ20491; HRS	0.00731457	2.00937
ILMN_1660193	STARD5	15	Homo sapiens StAR-related lipid transfer (START) domain containing 5 (STARD5), mRNA.	MGC10327	0.00680213	2.00925
ILMN_2208802	ALDOC	17	Homo sapiens aldolase C, fructose-bisphosphate (ALDOC), mRNA.	ALDC	0.00160603	2.00888
ILMN_1756982	LRRC1	6	Homo sapiens leucine rich repeat containing 1 (LRRC1), mRNA.	dJ523E19.1; LANO; FLJ11	0.009707	2.00846
ILMN_1804419	P4HA1	10	Homo sapiens procollagen-proline, 2-oxoglutarate 4-dioxygenase (proline 4-hydroxylase), alpha polypeptide I (P4HA1), transcript variant 1, mRNA.	P4HA; 4-PH alpha-1	0.0132439	2.00808

ILMN_2400759	RAC2	22	Homo sapiens ras-related C3 botulinum toxin substrate 2 (rho family, small GTP binding protein Rac2) (RAC2), mRNA.	Gx; EN-7; HSPC022	0.0297931	2.0078
ILMN_1781812	RXRA	9	Homo sapiens retinoid X receptor, alpha (RXRA), mRNA.	FLJ16020; FLJ16733; NR2	0.0133568	2.00691
ILMN_1698038	C11orf71	11	Homo sapiens chromosome 11 open reading frame 71 (C11orf71), mRNA.	FLJ20010	0.0385418	2.0059
ILMN_1722872	COL4A2	13	Homo sapiens collagen, type IV, alpha 2 (COL4A2), mRNA.	FLJ22259; DKFZp686I142	0.0261755	2.00565
ILMN_1679797	SPRYD4	12	Homo sapiens SPRY domain containing 4 (SPRYD4), mRNA.	DKFZp686N0877	0.00911102	2.00501
ILMN_1694432	SAA4	11	Homo sapiens serum amyloid A4, constitutive (SAA4), mRNA.	CSAA; C-SAA	0.0146507	2.00459
ILMN_1747160	BEX4	X	Homo sapiens BEX family member 4 (BEX4), mRNA.	BEXL1; BEX4; FLJ10097	0.00577082	2.00411
ILMN_2364376	PTGES	9	Homo sapiens prostaglandin E synthase (PTGES), mRNA.	mPGES-1; MGST1L1; MG	0.00190793	2.00345
ILMN_1727574	PSCD4	22	Homo sapiens pleckstrin homology, Sec7 and coiled-coil domains 4 (PSCD4), mRNA.	DJ63G5.1; CYT4	0.0376192	2.00294
ILMN_1779171	ZPLD1	3	Homo sapiens zona pellucida-like domain containing 1 (ZPLD1), mRNA.		0.0219516	2.0027
ILMN_1765310	GCHFR	15	Homo sapiens GTP cyclohydrolase I feedback regulator (GCHFR), mRNA.	HsT16933; MGC138467;	0.00252761	2.00246
ILMN_1788689	KIAA1881		PREDICTED: Homo sapiens KIAA1881 (KIAA1881), mRNA.		0.0162561	2.00216
ILMN_1757237	CRHBP	5	Homo sapiens corticotropin releasing hormone binding protein (CRHBP), mRNA.	CRFBP; CRF-BP	0.0186613	2.00195
ILMN_1677261	FER1L3	10	Homo sapiens fer-1-like 3, myoferlin (C. elegans) (FER1L3), transcript variant 2, mRNA.	FLJ36571; FLJ90777; MY(	0.00115636	2.00119

ILMN_1661342	SC5DL	11	Homo sapiens sterol-C5-desaturase (ERG3 delta-5-desaturase homolog, S. cerevisiae)-like (SC5DL), transcript variant 1, mRNA.	ERG3; SC5D; S5DES	0.0357156	2.00118
ILMN_1742544	SRGAP1	12	Homo sapiens SLIT-ROBO Rho GTPase activating protein 1 (SRGAP1), mRNA.	ARHGAP13; KIAA1304; FI	0.0156556	2.00083
ILMN_1912737	LOC730525		PREDICTED: Homo sapiens hypothetical protein LOC730525 (LOC730525), mRNA.		0.0108632	-2.00128
ILMN_1892608	ANXA11	10	Homo sapiens annexin A11 (ANXA11), transcript variant c, mRNA.	ANX11; CAP50	0.000743834	-2.00134
ILMN_1722407	TSPYL2	X	Homo sapiens TSPY-like 2 (TSPYL2), mRNA.	CDA1; CINAP; CTCL; SE2	2.29E-08	-2.00198
ILMN_1742109	PPP2R1B	11	Homo sapiens protein phosphatase 2 (formerly 2A), regulatory subunit A, beta isoform (PPP2R1B), transcript variant 2, mRNA.	MGC26454; PR65B	0.0035976	-2.00267
ILMN_1754304	MNDA	1	Homo sapiens myeloid cell nuclear differentiation antigen (MNDA), mRNA.	PYHIN3	0.0111264	-2.00494
ILMN_1750974	PMEPA1	20	Homo sapiens prostate transmembrane protein, androgen induced 1 (PMEPA1), transcript variant 2, mRNA.	STAG1; PMEPA1	0.0256156	-2.00535
ILMN_1675117	ACOT4	14	Homo sapiens acyl-CoA thioesterase 4 (ACOT4), mRNA.	PTE-Ib; PTE2B; PTE1B	0.0444027	-2.00664
ILMN_1812091	TAX1BP3	17	Homo sapiens Tax1 (human T-cell leukemia virus type I) binding protein 3 (TAX1BP3), mRNA.	TIP-1	0.0168257	-2.00798

ILMN_1661490	PLA2G4C	19	Homo sapiens phospholipase A2, group IVC (cytosolic, calcium-independent) (PLA2G4C), mRNA.	CPLA2-gamma; DKFZp58	0.00177672	-2.01167
ILMN_1691156	C1orf165	1	Homo sapiens chromosome 1 open reading frame 165 (C1orf165), mRNA.	FLJ11588	0.00330232	-2.01218
ILMN_1756402	SPARC	5	Homo sapiens secreted protein, acidic, cysteine-rich (osteonectin) (SPARC), mRNA.	ON	0.00925803	-2.0134
ILMN_1794505	LOC123876	16	Homo sapiens hypothetical protein LOC123876 (LOC123876), mRNA.	MGC150530; A-923A4.1	0.000434776	-2.01485
ILMN_1712707	MSRB3	12	Homo sapiens methionine sulfoxide reductase B3 (MSRB3), transcript variant 1, mRNA.	FLJ36866; DKFZp686C11	0.00945163	-2.01577
ILMN_1688242	NUDT5	10	Homo sapiens nudix (nucleoside diphosphate linked moiety X)-type motif 5 (NUDT5), mRNA.	hYSAH1; YSA1H	0.00223046	-2.01807
ILMN_1676413	CCL23	17	Homo sapiens chemokine (C-C motif) ligand 23 (CCL23), transcript variant CKbeta8, mRNA.	Ckb-8-1; CKb8; CK-BETA	0.046338	-2.0182
ILMN_2274923	LOC728888	16	PREDICTED: Homo sapiens similar to Protein KIAA0220 (LOC728888), mRNA.		0.0228774	-2.01922
ILMN_1774949	SEC14L4	22	Homo sapiens SEC14-like 4 (S. cerevisiae) (SEC14L4), mRNA.	TAP3	0.0200366	-2.01952
ILMN_1801378	ALPL	1	Homo sapiens alkaline phosphatase, liver/bone/kidney (ALPL), mRNA.	TNSALP; HOPS; FLJ40094	1.85E-05	-2.01973
ILMN_1752574	EFEMP1	2	Homo sapiens EGF-containing fibulin-like extracellular matrix protein 1 (EFEMP1), transcript variant 1, mRNA.	FBNL; MLVT; FLJ35535; F	0.00200239	-2.0217

ILMN_2067852	FKSG30	2	Homo sapiens actin-like protein (FKSG30), mRNA.	ACT	0.00677074	-2.02275
ILMN_1765668	SNAP25	20	Homo sapiens synaptosomal-associated protein, 25kDa (SNAP25), transcript variant 2, mRNA.	SNAP; dJ1068F16.2; SNA	0.0134551	-2.02309
ILMN_2410986	GBP2	1	Homo sapiens guanylate binding protein 2, interferon-inducible (GBP2), mRNA.		0.00246736	-2.02342
ILMN_1766054	ZBTB16	11	Homo sapiens zinc finger and BTB domain containing 16 (ZBTB16), transcript variant 1, mRNA.	PLZF; ZNF145	0.000902024	-2.02356
ILMN_1663220	LFNG	7	Homo sapiens LFNG O-fucosylpeptide 3-beta-N-acetylglucosaminyltransferase (LFNG), transcript variant 1, mRNA.	SCDO3	8.54E-06	-2.02542
ILMN_1809613	ILDR1	3	Homo sapiens immunoglobulin-like domain containing receptor 1 (ILDR1), mRNA.	ILDR1alpha; MGC50831;	0.00782111	-2.02776
ILMN_1663718	NR1I2	3	Homo sapiens nuclear receptor subfamily 1, group I, member 2 (NR1I2), transcript variant 3, mRNA.	PAR; PRR; SAR; PAR1; O	0.00138394	-2.03189
ILMN_1808301	C5orf13	5	Homo sapiens chromosome 5 open reading frame 13 (C5orf13), mRNA.	PTZ17; D4S114; PRO187	0.00365644	-2.03437
ILMN_1689156	AFMID	17	Homo sapiens arylformamidase (AFMID), mRNA.	KF; MGC167063; DKFZp6	0.00479997	-2.03485
ILMN_2223350	THADA	2	Homo sapiens thyroid adenoma associated (THADA), transcript variant 1, mRNA.	GITA; FLJ44016; KIAA17	0.0057117	-2.03599
ILMN_1691946			601452348F1 NIH_MGC_66 Homo sapiens cDNA clone IMAGE:3856355 5, mRNA sequence		4.30E-05	-2.0377

ILMN_1791678	OIT3	10	Homo sapiens oncoprotein induced transcript 3 (OIT3), mRNA.	LZP; FLJ39116	0.00623792	-2.03887
ILMN_1754055	MXRA8	1	Homo sapiens matrix-remodelling associated 8 (MXRA8), mRNA.	MGC3047; DKFZp586E20	0.0415432	-2.03957
ILMN_2194649	CBLN3	14	Homo sapiens cerebellin 3 precursor (CBLN3), mRNA.	PRO1486	0.0165369	-2.04221
ILMN_1696946	SAR1B	5	Homo sapiens SAR1 gene homolog B (S. cerevisiae) (SAR1B), transcript variant 1, mRNA.	CMRD; SARA2; GTBPB	0.0326459	-2.04356
ILMN_1759117	SNCAIP	5	Homo sapiens synuclein, alpha interacting protein (synphilin) (SNCAIP), mRNA.	MGC39814; SYPH1	0.0370416	-2.04689
ILMN_1725773	C1orf54	1	Homo sapiens chromosome 1 open reading frame 54 (C1orf54), mRNA.	FLJ23221	0.000774008	-2.04774
ILMN_1793628	DTX3	12	Homo sapiens deltex 3 homolog (Drosophila) (DTX3), mRNA.	FLJ34766; MGC138863; N	0.000121171	-2.04948
ILMN_2352609	LOC401845	16	PREDICTED: Homo sapiens similar to Ig heavy chain V-II region SESS precursor (LOC401845), mRNA.		0.00371459	-2.05018
ILMN_1761577	GPER	7	Homo sapiens G protein-coupled estrogen receptor 1 (GPER), transcript variant 3, mRNA.	LyGPR; DRY12; GPCR-Br;	0.00845901	-2.05059
ILMN_1700950	DLG4	17	Homo sapiens discs, large homolog 4 (Drosophila) (DLG4), mRNA.	SAP90; PSD95	0.00197651	-2.05232
ILMN_1776582	OGG1	3	Homo sapiens 8-oxoguanine DNA glycosylase (OGG1), nuclear gene encoding mitochondrial protein, transcript variant 2a, mRNA.	HOGG1; MUTM; HMMH; C	0.000309685	-2.05404

ILMN_1709408	INPP5D	2	Homo sapiens inositol polyphosphate-5-phosphatase, 145kDa (INPP5D), transcript variant 2, mRNA.	hp51CN; MGC142140; SH	0.00157578	-2.05876
ILMN_1676520	ABAT	16	Homo sapiens 4-aminobutyrate aminotransferase (ABAT), nuclear gene encoding mitochondrial protein, transcript variant 2, mRNA.	NPD009; GABAT; GABA-A	0.00355307	-2.05922
ILMN_1735347	JUNB	19	Homo sapiens jun B proto-oncogene (JUNB), mRNA.		0.00241644	-2.06014
ILMN_1805225	METRNL	17	Homo sapiens meteorin, glial cell differentiation regulator-like (METRNL), mRNA.	MGC99788	0.0127281	-2.06018
ILMN_1754421	COL5A1	9	Homo sapiens collagen, type V, alpha 1 (COL5A1), mRNA.		0.00113391	-2.06056
ILMN_1687315	OSBPL5	11	Homo sapiens oxysterol binding protein-like 5 (OSBPL5), transcript variant 1, mRNA.	ORP5; FLJ42929; OBPH1	0.00417236	-2.06242
ILMN_1912827	CCL8	17	Homo sapiens chemokine (C-C motif) ligand 8 (CCL8), mRNA.	HC14; MCP2; SCYA10; S(	2.05E-05	-2.06327
ILMN_1716979	CRIP2	14	Homo sapiens cysteine-rich protein 2 (CRIP2), mRNA.	CRP2; CRIP; ESP1	0.00751787	-2.06357
ILMN_1742947	CDH1	16	Homo sapiens cadherin 1, type 1, E-cadherin (epithelial) (CDH1), mRNA.	CDHE; Arc-1; UVO; CD32	0.00769423	-2.0649
ILMN_1750160	NUDT6	4	Homo sapiens nudix (nucleoside diphosphate linked moiety X)-type motif 6 (NUDT6), transcript variant 1, mRNA.	ASFGF2; gfg-1; FGF-AS;	0.000307056	-2.06764
ILMN_1672080	ADRA1A	8	Homo sapiens adrenergic, alpha-1A-, receptor (ADRA1A), transcript variant 2, mRNA.	ADRA1C; ALPHA1AAR; AD	0.0160088	-2.07137
ILMN_2112730	APOA5	11	Homo sapiens apolipoprotein A-V (APOA5), mRNA.	APOA-V; MGC126838; M(	3.32E-05	-2.07161

ILMN_1708833	CCDC102A	16	Homo sapiens coiled-coil domain containing 102A (CCDC102A), mRNA.	MGC13119; MGC10992	0.00155181	-2.07423
ILMN_1838949	EPHB6	7	Homo sapiens EPH receptor B6 (EPHB6), mRNA.	MGC129910; MGC129911	1.55E-05	-2.07814
ILMN_1686920	CD163	12	Homo sapiens CD163 molecule (CD163), transcript variant 2, mRNA.	MM130; M130	0.0187019	-2.07942
ILMN_1693352	EDN1	6	Homo sapiens endothelin 1 (EDN1), mRNA.	ET1	0.000100041	-2.07975
ILMN_2217809	HLA-DRB6	6	Homo sapiens major histocompatibility complex, class II, DR beta 6 (pseudogene) (HLA-DRB6) on chromosome 6.		1.54E-05	-2.08027
ILMN_1753005	C1QTNF5	11	Homo sapiens C1q and tumor necrosis factor related protein 5 (C1QTNF5), mRNA.	LORD; DKFZp586B0621;	0.0400935	-2.08305
ILMN_1755215	MYH9	22	Homo sapiens myosin, heavy chain 9, non-muscle (MYH9), mRNA.	NMMHCA; DFNA17; EPST	0.000854332	-2.08353
ILMN_1725510	JAM3	11	Homo sapiens junctional adhesion molecule 3 (JAM3), mRNA.	FLJ14529; JAMC; JAM-C	0.00393452	-2.08378
ILMN_1715543	TRO	X	Homo sapiens trophinin (TRO), transcript variant 6, mRNA.	KIAA1114; MAGE-d3; MA	0.00754805	-2.08437
ILMN_1795976	LAMC1	1	Homo sapiens laminin, gamma 1 (formerly LAMB2) (LAMC1), mRNA.	LAMB2; MGC87297	0.00152677	-2.08455
ILMN_1751086	CBR3	21	Homo sapiens carbonyl reductase 3 (CBR3), mRNA.	hCBR3	0.00080383	-2.08543
ILMN_2218002			BX097190 Soares placenta Nb2HP Homo sapiens cDNA clone IMAGp998G19212, mRNA sequence		0.0306253	-2.08579
ILMN_2155480	ZNF529	19	Homo sapiens zinc finger protein 529 (ZNF529), mRNA.	KIAA1615	0.0229306	-2.08778

ILMN_2069224	ADAMTS1	21	Homo sapiens ADAM metallopeptidase with thrombospondin type 1 motif, 1 (ADAMTS1), mRNA.	KIAA1346; C3-C5; METH1	0.0226189	-2.08893
ILMN_1696432	MRPL20	1	Homo sapiens mitochondrial ribosomal protein L20 (MRPL20), nuclear gene encoding mitochondrial protein, mRNA.	MGC4779; L20mt; MGC74	0.0148317	-2.08936
ILMN_2366714	PLEK	2	Homo sapiens pleckstrin (PLEK), mRNA.	P47; FLJ27168	0.00160014	-2.09076
ILMN_2406654	TAP1	6	Homo sapiens transporter 1, ATP-binding cassette, sub-family B (MDR/TAP) (TAP1), mRNA.	ABC17; PSF1; FLJ26666;	0.00394114	-2.09198
ILMN_1679929	CX3CL1	16	Homo sapiens chemokine (C-X3-C motif) ligand 1 (CX3CL1), mRNA.	NTN; CXC3C; NTT; fracta	2.55E-05	-2.09342
ILMN_1802257	LSP1	11	Homo sapiens lymphocyte-specific protein 1 (LSP1), transcript variant 2, mRNA.	WP34; pp52	0.00477512	-2.09385
ILMN_2357334	LIPC	15	Homo sapiens lipase, hepatic (LIPC), mRNA.	LIPH; HL; HTGL	0.01906	-2.09474
ILMN_1749751	NUDT6	4	Homo sapiens nudix (nucleoside diphosphate linked moiety X)-type motif 6 (NUDT6), transcript variant 1, mRNA.	ASFGF2; gfg-1; FGF-AS;	0.000489304	-2.09539
ILMN_1783469	INHBE	12	Homo sapiens inhibin, beta E (INHBE), mRNA.	MGC4638	0.00698095	-2.09614
ILMN_1687440	MTHFS	15	Homo sapiens 5,10-methenyltetrahydrofolate synthetase (5-formyltetrahydrofolate cyclo-ligase) (MTHFS), mRNA.	HsT19268	0.00181516	-2.09657
ILMN_1805922	PLXNA1	3	Homo sapiens plexin A1 (PLXNA1), mRNA.	NOV; PLXN1; NOVP; PLEX	0.0133646	-2.09754

ILMN_1668055	PLAGL1	6	Homo sapiens pleiomorphic adenoma gene-like 1 (PLAGL1), transcript variant 3, mRNA.	MGC126276; LOT1; ZAC;	0.0008995	-2.09966
ILMN_1722622	PDE5A	4	Homo sapiens phosphodiesterase 5A, cGMP-specific (PDE5A), transcript variant 1, mRNA.	CGB-PDE; PDE5; PDE5A1	0.0114276	-2.10455
ILMN_1690371	ADRA1A	8	Homo sapiens adrenergic, alpha-1A-, receptor (ADRA1A), transcript variant 3, mRNA.	ADRA1C; ALPHA1AAR; AI	3.11E-06	-2.10464
ILMN_1725692	ADAMTS5	21	Homo sapiens ADAM metallopeptidase with thrombospondin type 1 motif, 5 (aggrecanase-2) (ADAMTS5), mRNA.	ADAMTS11; FLJ36738; AI	1.25E-05	-2.10992
ILMN_2346831	HBA2	16	Homo sapiens hemoglobin, alpha 2 (HBA2), mRNA.	HBA1	0.000141286	-2.11101
ILMN_1733270	UGDH	4	Homo sapiens UDP-glucose dehydrogenase (UGDH), mRNA.	UDPGDH; UGD; UDP-GlcD	0.000464545	-2.11138
ILMN_2379599	HBB	11	Homo sapiens hemoglobin, beta (HBB), mRNA.	HBD; CD113t-C	0.00231483	-2.11201
ILMN_1735367	LOC648470		PREDICTED: Homo sapiens similar to Caspase-4 precursor (CASP-4) (ICH-2 protease) (TX protease) (ICE(rel)-II) (LOC648470), mRNA.		0.0214233	-2.1157
ILMN_1816634	PACS1	11	Homo sapiens phosphofurin acidic cluster sorting protein 1 (PACS1), mRNA.	FLJ10209; KIAA1175	0.0438303	-2.11772
ILMN_1727815	TPM4	19	Homo sapiens tropomyosin 4 (TPM4), mRNA.		0.00508228	-2.11876
ILMN_1751072	PDE1A	2	Homo sapiens phosphodiesterase 1A, calmodulin-dependent (PDE1A), transcript variant 2, mRNA.	HSPDE1A; MGC26303; H(	0.00143711	-2.11897

ILMN_1750496	PFDN6	6	Homo sapiens prefoldin subunit 6 (PFDN6), mRNA.	HKE2; PFD6; H2-KE2; KE	0.0112528	-2.12046
ILMN_2341724	HKDC1	10	Homo sapiens hexokinase domain containing 1 (HKDC1), mRNA.	MGC125688; FLJ37767; F	0.00110783	-2.12154
ILMN_1737683	CLEC11A	19	Homo sapiens C-type lectin domain family 11, member A (CLEC11A), mRNA.	LSLCL; P47; SCGF; CLECS	0.000583539	-2.12216
ILMN_1706873	LRG1	19	Homo sapiens leucine-rich alpha-2-glycoprotein 1 (LRG1), mRNA.	HMFT1766; LRG	3.58E-05	-2.12305
ILMN_1799579	GYPC	2	Homo sapiens glycophorin C (Gerbich blood group) (GYPC), transcript variant 2, mRNA.	MGC126191; GE; CD236F	0.000226659	-2.12329
ILMN_1741096	MCEE	2	Homo sapiens methylmalonyl CoA epimerase (MCEE), mRNA.	GLOD2	0.00348302	-2.12471
ILMN_1662942	ROR2	9	Homo sapiens receptor tyrosine kinase-like orphan receptor 2 (ROR2), mRNA.	BDB; BDB1; MGC163394	0.0190925	-2.12499
ILMN_1714197	EPHB1	3	Homo sapiens EPH receptor B1 (EPHB1), mRNA.	Hek6; EPHT2; NET; FLJ37	0.0194486	-2.12507
ILMN_1691526	C20orf3	20	Homo sapiens chromosome 20 open reading frame 3 (C20orf3), mRNA.	BSCv; APMAP	4.98E-05	-2.126
ILMN_1733374	PTPN3	9	Homo sapiens protein tyrosine phosphatase, non-receptor type 3 (PTPN3), mRNA.	DKFZp686N0569; PTPH1	0.000947137	-2.12693
ILMN_1678173	LOC134145	5	Homo sapiens hypothetical protein LOC134145 (LOC134145), mRNA.	FLJ20667	0.0395328	-2.12777
ILMN_2372915	ZNF789	7	Homo sapiens zinc finger protein 789 (ZNF789), transcript variant 1, mRNA.		0.000749259	-2.12843
ILMN_2113490	C21orf63	21	Homo sapiens chromosome 21 open reading frame 63 (C21orf63), mRNA.	PRED34; B19; SUE21; B1	8.69E-05	-2.13064
ILMN_2244841	6-Sep	X	Homo sapiens septin 6 (SEPT6), transcript variant I, mRNA.	SEP2; RP5-876A24.2; MG	0.0056184	-2.13118

ILMN_1690252	FER1L3	10	Homo sapiens fer-1-like 3, myoferlin (C. elegans) (FER1L3), transcript variant 1, mRNA.	FLJ36571; FLJ90777; MY(	0.00203854	-2.1327
ILMN_1843721	ADFP	9	Homo sapiens adipose differentiation-related protein (ADFP), mRNA.	ADRP; MGC10598	0.00954343	-2.13325
ILMN_1764175	C1orf116	1	Homo sapiens chromosome 1 open reading frame 116 (C1orf116), mRNA.	MGC4309; DKFZp666H20	0.0186892	-2.1333
ILMN_1750658			PREDICTED: Homo sapiens hypothetical LOC400043 (LOC400043), mRNA		0.000617485	-2.13442
ILMN_2075334	FNDC1	6	Homo sapiens fibronectin type III domain containing 1 (FNDC1), mRNA.	bA243O10.1; AGS8; RP1	0.000400751	-2.13524
ILMN_1772123	KLF2	19	Homo sapiens Kruppel-like factor 2 (lung) (KLF2), mRNA.	LKLF	0.00633638	-2.13546
ILMN_1655051	GPR109B		PREDICTED: Homo sapiens G protein-coupled receptor 109B, transcript variant 3 (GPR109B), mRNA.		0.00211538	-2.13663
ILMN_2337923	C3orf23	3	Homo sapiens chromosome 3 open reading frame 23 (C3orf23), transcript variant 3, mRNA.	MGC119533; MGC11953(	0.00158288	-2.1377
ILMN_2049693	PC	11	Homo sapiens pyruvate carboxylase (PC), nuclear gene encoding mitochondrial protein, transcript variant A, mRNA.	PCB	0.000125263	-2.13777
ILMN_1651987	SAA1	11	Homo sapiens serum amyloid A1 (SAA1), transcript variant 1, mRNA.	MGC111216; SAA; PIG4;	0.0229625	-2.13814
ILMN_1682812	S100A9	1	Homo sapiens S100 calcium binding protein A9 (calgranulin B) (S100A9), mRNA.	CFAG; MAC387; 60B8AG;	1.34E-05	-2.13949
ILMN_2189424	PZP	12	Homo sapiens pregnancy-zone protein (PZP), mRNA.	CPAMD6; MGC133093	0.000208143	-2.14284

ILMN_1710315	CD34	1	Homo sapiens CD34 molecule (CD34), transcript variant 2, mRNA.		3.63E-05	-2.14491
ILMN_1717294	GPR128	3	Homo sapiens G protein-coupled receptor 128 (GPR128), mRNA.	MGC163260; FLJ29035; M	0.00206714	-2.14538
ILMN_1687864	MOBKL2B	9	Homo sapiens MOB1, Mps One Binder kinase activator-like 2B (yeast) (MOBKL2B), mRNA.	FLJ13204; FLJ23916; MO	0.00342477	-2.1473
ILMN_2349444	ABI3BP	3	Homo sapiens ABI gene family, member 3 (NESH) binding protein (ABI3BP), mRNA.	FLJ41743; FLJ41754; TAF	0.00383246	-2.14851
ILMN_1727091	ZNF532	18	Homo sapiens zinc finger protein 532 (ZNF532), mRNA.	FLJ10697	0.000416606	-2.14862
ILMN_1732538	ZBTB16	11	Homo sapiens zinc finger and BTB domain containing 16 (ZBTB16), transcript variant 2, mRNA.	PLZF; ZNF145	0.0397455	-2.14907
ILMN_1670117	MT1G	16	Homo sapiens metallothionein 1G (MT1G), mRNA.	MT1; MT1K; MGC12386	0.000161988	-2.1497
ILMN_2311089	LOC651621		PREDICTED: Homo sapiens similar to hydroxysteroid (17-beta) dehydrogenase 7 (LOC651621), mRNA.		0.00319861	-2.1512
ILMN_1669881	CSAG3B		Homo sapiens CSAG family, member 3B (CSAG3B), mRNA.	CSAG2	0.0480861	-2.15319
ILMN_1736888	CLIP3	19	Homo sapiens CAP-GLY domain containing linker protein 3 (CLIP3), mRNA.	RSNL1; CLIPR-59; CLIPR5	0.0034469	-2.16071
ILMN_1716237	PDE1A	2	Homo sapiens phosphodiesterase 1A, calmodulin-dependent (PDE1A), transcript variant 2, mRNA.	HSPDE1A; MGC26303; H	0.00935529	-2.1614
ILMN_1702168	LOC653506	17	PREDICTED: Homo sapiens similar to meteorin, glial cell differentiation regulator-like (LOC653506), mRNA.		0.000523211	-2.16261

ILMN_1657760	PDLIM3	4	Homo sapiens PDZ and LIM domain 3 (PDLIM3), mRNA.	DKFZp686L0362; ALP	0.0491863	-2.16575
ILMN_1785336	SCRN1	7	Homo sapiens secernin 1 (SCRN1), mRNA.	SES1; KIAA0193	0.00403005	-2.16704
ILMN_1713008	PTPN13	4	Homo sapiens protein tyrosine phosphatase, non-receptor type 13 (APO-1/CD95 (Fas)-associated phosphatase) (PTPN13), transcript variant 3, mRNA.	PTP1E; PNP1; PTP-BL; PT	0.00534155	-2.16717
ILMN_1681520	CCL23	17	Homo sapiens chemokine (C-C motif) ligand 23 (CCL23), transcript variant CKbeta8-1, mRNA.	Ckb-8-1; CKb8; CK-BETA	0.00161339	-2.16732
ILMN_1758173	MYH10	17	Homo sapiens myosin, heavy chain 10, non-muscle (MYH10), mRNA.	NMMHCB; MGC134914; M	0.000581643	-2.17216
ILMN_1790228	AHSG	3	Homo sapiens alpha-2-HS-glycoprotein (AHSG), mRNA.	HSGA; A2HS; AHS; FETU	0.0174384	-2.17327
ILMN_2355971	DOCK10	2	Homo sapiens dedicator of cytokinesis 10 (DOCK10), mRNA.	Nbla10300; DKFZp781A1	0.0378765	-2.17442
ILMN_1811767	KCTD12	13	Homo sapiens potassium channel tetramerisation domain containing 12 (KCTD12), mRNA.	FLJ33073; KIAA1778; C1	0.0237916	-2.17459
ILMN_1790781	LEPR	1	Homo sapiens leptin receptor (LEPR), transcript variant 1, mRNA.	OBR; CD295	0.0178883	-2.17821
ILMN_2070815	OLFML3	1	Homo sapiens olfactomedin-like 3 (OLFML3), mRNA.	OLF44; HNOEL-iso	0.000529852	-2.17909
ILMN_1681938	PAM	5	Homo sapiens peptidylglycine alpha-amidating monooxygenase (PAM), transcript variant 3, mRNA.	PHM; PAL	0.0306314	-2.18023
ILMN_1724293	PPP1R15A	19	Homo sapiens protein phosphatase 1, regulatory (inhibitor) subunit 15A (PPP1R15A), mRNA.	GADD34	0.00326256	-2.18092

ILMN_2090782	S100A4	1	Homo sapiens S100 calcium binding protein A4 (S100A4), transcript variant 2, mRNA.	FSP1; MTS1; 18A2; PEL9	0.00179798	-2.18155
ILMN_2054392	TTYH1	19	Homo sapiens tweety homolog 1 (Drosophila) (TTYH1), transcript variant 1, mRNA.		0.000577895	-2.18167
ILMN_1653871	DPH5	1	Homo sapiens DPH5 homolog (S. cerevisiae) (DPH5), transcript variant 1, mRNA.	MGC61450; AD-018; NPD	0.00116093	-2.18311
ILMN_1779841	ALDH4A1	1	Homo sapiens aldehyde dehydrogenase 4 family, member A1 (ALDH4A1), nuclear gene encoding mitochondrial protein, transcript variant P5CDhL, mRNA.	P5CDh; P5CD; ALDH4; P5	0.0117734	-2.19271
ILMN_1723607	DKK3	11	Homo sapiens dickkopf homolog 3 (Xenopus laevis) (DKK3), transcript variant 2, mRNA.	REIC	0.00255322	-2.19388
ILMN_1669409			BX109986 Soares fetal liver spleen 1NFLS Homo sapiens cDNA clone IMAGp998K14124, mRNA sequence		0.00458558	-2.19722
ILMN_1749996	SFXN2	10	Homo sapiens sideroflexin 2 (SFXN2), mRNA.		0.00253684	-2.19919
ILMN_1757134	DDEF2	2	Homo sapiens development and differentiation enhancing factor 2 (DDEF2), mRNA.	PAG3; Pap-alpha; SHAG1	0.01459	-2.20674
ILMN_1700268	EGR1	5	Homo sapiens early growth response 1 (EGR1), mRNA.	G0S30; AT225; TIS8; ZN	0.0358255	-2.20734
ILMN_1771348	FNDC1	6	Homo sapiens fibronectin type III domain containing 1 (FNDC1), mRNA.	bA243O10.1; AGS8; RP1[illegible]	8.79E-06	-2.20772
ILMN_1802894	ATP1B3		PREDICTED: Homo sapiens ATPase, Na+/K+ transporting, beta 3 polypeptide, transcript variant 2 (ATP1B3), mRNA.		4.79E-05	-2.20837

ILMN_1737943	PMP22	17	Homo sapiens peripheral myelin protein 22 (PMP22), transcript variant 2, mRNA.	CMT1A; GAS-3; DSS; CM	0.00387616	-2.20917
ILMN_1654109	CHD3	17	Homo sapiens chromodomain helicase DNA binding protein 3 (CHD3), transcript variant 1, mRNA.	Mi2-ALPHA; Mi-2a; ZFH	0.000562178	-2.21224
ILMN_2073604	EPHA3	3	Homo sapiens EPH receptor A3 (EPHA3), transcript variant 1, mRNA.	HEK4; ETK1; HEK; TYRO4	0.00762257	-2.21375
ILMN_1744244	HLA-DRB3	6	Homo sapiens major histocompatibility complex, class II, DR beta 3 (HLA-DRB3), mRNA.	MGC117330; HLA-DR3B	0.00763141	-2.21386
ILMN_1690282	RHBDF1	16	Homo sapiens rhomboid 5 homolog 1 (Drosophila) (RHBDF1), mRNA.	gene -90; gene -89; C16	0.00112942	-2.21513
ILMN_1731044	CDR2L	17	Homo sapiens cerebellar degeneration-related protein 2-like (CDR2L), mRNA.	HUMPPA	3.30E-05	-2.21515
ILMN_2212763	LAT	16	Homo sapiens linker for activation of T cells (LAT), transcript variant 2, mRNA.	pp36; LAT1	0.00669875	-2.21586
ILMN_1786024	CD74	5	Homo sapiens CD74 molecule, major histocompatibility complex, class II invariant chain (CD74), transcript variant 1, mRNA.	Ia-GAMMA; DHLAG; HLAD	0.000551301	-2.21711
ILMN_1652161	IL1R2	2	Homo sapiens interleukin 1 receptor, type II (IL1R2), transcript variant 2, mRNA.	IL1RB; CD121b; MGC477	0.000207181	-2.21792
ILMN_1798971	CMTM7	3	Homo sapiens CKLF-like MARVEL transmembrane domain containing 7 (CMTM7), transcript variant 2, mRNA.	CKLFSF7; FLJ30992	0.000752429	-2.218
ILMN_2366972			AGENCOURT_10400346 NIH_MGC_82 Homo sapiens cDNA clone IMAGE:6616124 5, mRNA sequence		0.000983118	-2.21849

ILMN_1765971	COL5A2	2	Homo sapiens collagen, type V, alpha 2 (COL5A2), mRNA.	MGC105115	0.00110371	-2.21865
ILMN_1789003	MVK	12	Homo sapiens mevalonate kinase (mevalonic aciduria) (MVK), mRNA.	MVLK; LRBP	0.00119784	-2.22324
ILMN_1755974	METRNL		PREDICTED: Homo sapiens meteorin, glial cell differentiation regulator-like (METRNL), mRNA.		0.0229111	-2.22452
ILMN_1909886	MGC4677	2	Homo sapiens hypothetical protein MGC4677 (MGC4677), mRNA.	MGC17532; MGC88182	0.000364615	-2.22559
ILMN_1811345	CASP1	11	Homo sapiens caspase 1, apoptosis-related cysteine peptidase (interleukin 1, beta, convertase) (CASP1), transcript variant delta, mRNA.	IL1BC; ICE; P45	0.0429803	-2.22735
ILMN_1684585	ARHGEF10	8	Homo sapiens Rho guanine nucleotide exchange factor (GEF) 10 (ARHGEF10), mRNA.	GEF10; DKFZp686H0726	3.35E-05	-2.22772
ILMN_1787556	ITGA2	5	Homo sapiens integrin, alpha 2 (CD49B, alpha 2 subunit of VLA-2 receptor) (ITGA2), mRNA.	CD49B; VLAA2; VLA-2; B	0.0161056	-2.2293
ILMN_1686989	C9	5	Homo sapiens complement component 9 (C9), mRNA.		0.00467219	-2.22943
ILMN_1766657	AMICA1	11	Homo sapiens adhesion molecule, interacts with CXADR antigen 1 (AMICA1), mRNA.	MGC118814; AMICA; FLJ	0.000537793	-2.22963
ILMN_1688041	SYT15	10	Homo sapiens synaptotagmin XV (SYT15), transcript variant b, mRNA.	sytXV; CHR10SYT; syt XV	4.81E-05	-2.23076
ILMN_1710124			Homo sapiens mRNA; cDNA DKFZp686J0156 (from clone DKFZp686J0156)		0.0155125	-2.23335
ILMN_1700695	VEGFB	11	Homo sapiens vascular endothelial growth factor B (VEGFB), mRNA.	VRF; VEGFL	0.000611587	-2.23377

ILMN_1784380	3-Mar		PREDICTED: Homo sapiens membrane-associated ring finger (C3HC4) 3 (MARCH3), mRNA.		0.00208102	-2.23614
ILMN_2095653	RGS10	10	Homo sapiens regulator of G-protein signaling 10 (RGS10), transcript variant 1, mRNA.		0.0147793	-2.23674
ILMN_1696099	CPXM2	10	Homo sapiens carboxypeptidase X (M14 family), member 2 (CPXM2), mRNA.	UNQ676	0.0031561	-2.24246
ILMN_1673681	CYGB	17	Homo sapiens cytoglobin (CYGB), mRNA.	HGB; STAP	0.00112379	-2.24414
ILMN_1663351	ADK	10	Homo sapiens adenosine kinase (ADK), transcript variant ADK-short, mRNA.	AK	0.000100338	-2.24492
ILMN_1665192	IL1B	2	Homo sapiens interleukin 1, beta (IL1B), mRNA.	IL1F2; IL1-BETA; IL-1	0.00188432	-2.24661
ILMN_1786105	C13orf33	13	Homo sapiens chromosome 13 open reading frame 33 (C13orf33), mRNA.	MGC126673; MGC126675	0.00179509	-2.24831
ILMN_1770048	LOC650369		PREDICTED: Homo sapiens similar to family with sequence similarity 60, member A (LOC650369), mRNA.		8.15E-08	-2.24986
ILMN_1803194	REC8	14	Homo sapiens REC8 homolog (yeast) (REC8), transcript variant 1, mRNA.	HR21spB; REC8; MGC950	0.0171849	-2.25296
ILMN_2406557	CXCL13	4	Homo sapiens chemokine (C-X-C motif) ligand 13 (B-cell chemoattractant) (CXCL13), mRNA.	SCYB13; ANGIE; BCA1; A	0.000624707	-2.25366
ILMN_2383349	MT1B	16	Homo sapiens metallothionein 1B (MT1B), mRNA.	MTP; MT1; MT1Q	0.000260885	-2.2543
ILMN_1682957	MAPRE1	20	Homo sapiens microtubule-associated protein, RP/EB family, member 1 (MAPRE1), mRNA.	EB1; MGC129946; MGC1	0.000640076	-2.25557

ILMN_1810560	HYOU1	11	Homo sapiens hypoxia up-regulated 1 (HYOU1), mRNA.	DKFZp686N08236; ORP1	0.0410531	-2.25825
ILMN_1715680	MOGAT1	2	Homo sapiens monoacylglycerol O-acyltransferase 1 (MOGAT1), mRNA.	DGAT2L1; DGAT2L; MGAT	0.0105934	-2.25838
ILMN_1751753	F13A1	6	Homo sapiens coagulation factor XIII, A1 polypeptide (F13A1), mRNA.	F13A	0.000234231	-2.25947
ILMN_1651950	SAA2	11	Homo sapiens serum amyloid A2 (SAA2), mRNA.		0.00146212	-2.26397
ILMN_1763523	ANXA11	10	Homo sapiens annexin A11 (ANXA11), transcript variant a, mRNA.	CAP50; ANX11	0.000109949	-2.26494
ILMN_1764321	FZD5	2	Homo sapiens frizzled homolog 5 (Drosophila) (FZD5), mRNA.	DKFZP434E2135; C2orf31	0.000152444	-2.26511
ILMN_1782237	MOXD1	6	Homo sapiens monooxygenase, DBH-like 1 (MOXD1), transcript variant 2, mRNA.	MOX; dJ248E1.1; PRO578	2.09E-06	-2.26548
ILMN_1769158	ADORA3	1	Homo sapiens adenosine A3 receptor (ADORA3), transcript variant 3, mRNA.	AD026; RP11-552M11.7;	0.0221563	-2.26562
ILMN_1748915	MICAL1	6	Homo sapiens microtubule associated monoxygenase, calponin and LIM domain containing 1 (MICAL1), mRNA.	NICAL; DKFZp434B1517;	0.0103228	-2.26688
ILMN_1704656	FAM129A	1	Homo sapiens family with sequence similarity 129, member A (FAM129A), transcript variant 2, mRNA.	NIBAN; FLJ38228; C1orf2	0.00651254	-2.26712
ILMN_1672589	STEAP1		PREDICTED: Homo sapiens six transmembrane epithelial antigen of the prostate 1 (STEAP1), mRNA.		0.00524691	-2.26717
ILMN_1663640			nae09h04.x1 NCI_CGAP_Ov18 Homo sapiens cDNA clone IMAGE:3435102 3, mRNA sequence		0.00247416	-2.26976

ILMN_1795906	GDPD3	16	Homo sapiens glycerophosphodiester phosphodiesterase domain containing 3 (GDPD3), mRNA.	FLJ22603; MGC4171	0.000324381	-2.27241
ILMN_1732750	DUSP5	10	Homo sapiens dual specificity phosphatase 5 (DUSP5), mRNA.	HVH3; DUSP	6.01E-05	-2.27329
ILMN_1652722	SEC61A2	10	Homo sapiens Sec61 alpha 2 subunit (S. cerevisiae) (SEC61A2), mRNA.	FLJ10578	0.00301018	-2.27438
ILMN_1668460	LIPG	18	Homo sapiens lipase, endothelial (LIPG), mRNA.	EL; PRO719; EDL	0.000950616	-2.27588
ILMN_1692261	CRIP1	14	Homo sapiens cysteine-rich protein 1 (intestinal) (CRIP1), mRNA.	CRIP; CRP1; CRHP	0.000909894	-2.27713
ILMN_1699987	PTRF	17	Homo sapiens polymerase I and transcript release factor (PTRF), mRNA.	FKSG13	5.20E-06	-2.27883
ILMN_1815102	TMEM51	1	Homo sapiens transmembrane protein 51 (TMEM51), mRNA.	FLJ10199; C1orf72	0.0192406	-2.27926
ILMN_2331636	FGD3	9	Homo sapiens FYVE, RhoGEF and PH domain containing 3 (FGD3), transcript variant 2, mRNA.	MGC117260; FLJ00004; Z	0.0118203	-2.28136
ILMN_2358626	LPAL2	6	Homo sapiens lipoprotein, Lp(a)-like 2 (LPAL2), transcript variant 2, mRNA.	RP11-72O9.2; MGC12953	6.57E-05	-2.28321
ILMN_1806561	LOC641825		PREDICTED: Homo sapiens hypothetical protein LOC641825 (LOC641825), mRNA.		0.000193764	-2.28856
ILMN_1737308	PRTN3	19	Homo sapiens proteinase 3 (serine proteinase, neutrophil, Wegener granulomatosis autoantigen) (PRTN3), mRNA.	P29; MBT; AGP7; PR-3; C	4.70E-05	-2.29014
ILMN_1760506	NIN	14	Homo sapiens ninein (GSK3B interacting protein) (NIN), transcript variant 2, mRNA.	KIAA1565	0.00328394	-2.29395
ILMN_1743711	DOCK4	7	Homo sapiens dedicator of cytokinesis 4 (DOCK4), mRNA.	MGC134911; FLJ34238; M	8.76E-05	-2.29555

ILMN_1662768	PDLIM3	4	Homo sapiens PDZ and LIM domain 3 (PDLIM3), mRNA.	DKFZp686L0362; ALP	1.22E-05	-2.29868
ILMN_1815261	C13orf1	13	Homo sapiens chromosome 13 open reading frame 1 (C13orf1), mRNA.	CLLD6	0.0182459	-2.29964
ILMN_1783337	GPR56	16	Homo sapiens G protein-coupled receptor 56 (GPR56), transcript variant 3, mRNA.	BFPP; DKFZp781L1398; T	0.00295864	-2.29985
ILMN_2336595	FURIN	15	Homo sapiens furin (paired basic amino acid cleaving enzyme) (FURIN), mRNA.	PACE; FUR; SPC1; PCSK3	0.016526	-2.30586
ILMN_1668219	ABHD6	3	Homo sapiens abhydrolase domain containing 6 (ABHD6), mRNA.		0.00297308	-2.30841
ILMN_1801044	FRZB	2	Homo sapiens frizzled-related protein (FRZB), mRNA.	FRZB-1; FRZB-PEN; SRFP	0.00809353	-2.31608
ILMN_1909468	GRRP1	1	Homo sapiens glycine/arginine rich protein 1 (GRRP1), mRNA.	RP11-96L14.5; FLJ14050	0.000249021	-2.31831
ILMN_1654064	MTE		Homo sapiens metallothionein E (MTE), mRNA.	MT1I	0.00689114	-2.3272
ILMN_1661599	SLC25A30	13	Homo sapiens solute carrier family 25, member 30 (SLC25A30), mRNA.	KMCP1	0.00951052	-2.334
ILMN_1754137	IGFBP7	4	Homo sapiens insulin-like growth factor binding protein 7 (IGFBP7), mRNA.	MAC25; IGFBP-7; IGFBP-	0.0305795	-2.33688
ILMN_1711314	DGAT2	11	Homo sapiens diacylglycerol O-acyltransferase homolog 2 (mouse) (DGAT2), mRNA.	HMFN1045; DKFZp686A1	0.000238183	-2.34309
ILMN_1782429	RABEPK	9	Homo sapiens Rab9 effector protein with kelch motifs (RABEPK), mRNA.	bA65N13.1; DKFZp686P1	0.000837002	-2.3431
ILMN_1787526	LOC649495		PREDICTED: Homo sapiens similar to Alpha-tocopherol transfer protein (Alpha-TTP) (LOC649495), mRNA.		0.00230619	-2.34496
ILMN_2120247	IRF1	5	Homo sapiens interferon regulatory factor 1 (IRF1), mRNA.	IRF-1; MAR	7.30E-06	-2.34795

ILMN_2251766	CD5	11	Homo sapiens CD5 molecule (CD5), mRNA.	T1; LEU1	0.000690804	-2.34851
ILMN_2280203	C6orf111	6	Homo sapiens chromosome 6 open reading frame 111 (C6orf111), mRNA.	DKFZp564B0769; FLJ901	5.91E-07	-2.34911
ILMN_1670817	PLAC9	10	Homo sapiens placenta-specific 9 (PLAC9), mRNA.	MGC104710	3.42E-06	-2.35091
ILMN_1871361	NAP1L1	12	Homo sapiens nucleosome assembly protein 1-like 1 (NAP1L1), transcript variant 1, mRNA.	NAP1; NRP; NAP1L; FLJ1	0.0291813	-2.35141
ILMN_1727840	FCN3	1	Homo sapiens ficolin (collagen/fibrinogen domain containing) 3 (Hakata antigen) (FCN3), transcript variant 1, mRNA.	MGC22543; FCNH; HAKA	0.00012868	-2.3543
ILMN_1798298	ITGB5	3	Homo sapiens integrin, beta 5 (ITGB5), mRNA. XM_944688 XM_944693	FLJ26658	0.00942353	-2.35472
ILMN_2183510	GPNMB	7	Homo sapiens glycoprotein (transmembrane) nmb (GPNMB), transcript variant 2, mRNA.	NMB; HGFIN	0.0029463	-2.35924
ILMN_2367428	LGALS3BP	17	Homo sapiens lectin, galactoside-binding, soluble, 3 binding protein (LGALS3BP), mRNA.	MAC-2-BP; 90K	0.000310361	-2.36031
ILMN_1793287	RPL29	3	Homo sapiens ribosomal protein L29 (RPL29), mRNA.	HUMRPL29; MGC88589;	9.81E-05	-2.36301
ILMN_1664283	RPL34	4	Homo sapiens ribosomal protein L34 (RPL34), transcript variant 1, mRNA.		0.0344626	-2.36315
ILMN_1780659	ALDH1A3	15	Homo sapiens aldehyde dehydrogenase 1 family, member A3 (ALDH1A3), mRNA.	RALDH3; ALDH6; ALDH1	0.0016831	-2.36825
ILMN_2400183	CCDC109B	4	Homo sapiens coiled-coil domain containing 109B (CCDC109B), mRNA.	FLJ20647	2.03E-08	-2.37068

ILMN_1773865	STAMBPL1	10	Homo sapiens STAM binding protein-like 1 (STAMBPL1), mRNA.	bA399O19.2; AMSH-LP; A	1.20E-05	-2.37097
ILMN_1734929	DUSP1	5	Homo sapiens dual specificity phosphatase 1 (DUSP1), mRNA.	HVH1; MKP1; MKP-1; PTP	0.028442	-2.37244
ILMN_2113126	CUGBP2	10	Homo sapiens CUG triplet repeat, RNA binding protein 2 (CUGBP2), transcript variant 2, mRNA.	BRUNOL3; ETR-3; NAPOR	0.0372717	-2.3731
ILMN_1707484	TUBB6	18	Homo sapiens tubulin, beta 6 (TUBB6), mRNA.	MGC132410; HsT1601; M	1.75E-05	-2.37389
ILMN_2386205	PCDH20	13	Homo sapiens protocadherin 20 (PCDH20), mRNA.	PCDH13; FLJ22218	6.20E-06	-2.37396
ILMN_1719661	APCS	1	Homo sapiens amyloid P component, serum (APCS), mRNA.	SAP; MGC88159; PTX2	0.0155566	-2.37461
ILMN_2256050	LRMP	12	Homo sapiens lymphoid-restricted membrane protein (LRMP), mRNA.	JAW1	0.0219967	-2.37925
ILMN_2049184	SAMD9L	7	Homo sapiens sterile alpha motif domain containing 9-like (SAMD9L), mRNA.	FLJ39885; DRIF2; C7orf6	0.00994917	-2.38412
ILMN_2067709	S100A10	1	Homo sapiens S100 calcium binding protein A10 (S100A10), mRNA.	ANX2L; MGC111133; GP1	0.00027838	-2.3847
ILMN_2370414	LAT	16	Homo sapiens linker for activation of T cells (LAT), transcript variant 2, mRNA.	pp36; LAT1	8.50E-07	-2.38504
ILMN_1772409	LOC646567	1	PREDICTED: Homo sapiens similar to HSPC307 (LOC646567), mRNA.		0.000688319	-2.38762
ILMN_1672504	C6orf115	6	Homo sapiens chromosome 6 open reading frame 115 (C6orf115), mRNA.	HSPC280; PRO2013	0.00489705	-2.38778
ILMN_1731688	MGC39900		PREDICTED: Homo sapiens hypothetical protein MGC39900 (MGC39900), mRNA.		0.000195434	-2.38825

ILMN_1706344	POLR3H	22	Homo sapiens polymerase (RNA) III (DNA directed) polypeptide H (22.9kD) (POLR3H), transcript variant 2, mRNA.	RPC8; MGC29654; MGC1	4.32E-05	-2.38989
ILMN_1744006	KIAA0746	4	Homo sapiens KIAA0746 protein (KIAA0746), mRNA.	FLJ21629; FLJ41299; DKI	0.00144806	-2.39398
ILMN_1766918	TMED6	16	Homo sapiens transmembrane emp24 protein transport domain containing 6 (TMED6), mRNA.	PRO34237; SPLL9146; M(	8.64E-06	-2.39482
ILMN_1754517	ABLIM2	4	Homo sapiens actin binding LIM protein family, member 2 (ABLIM2), mRNA.	FLJ39684; KIAA1808; DK	0.00653631	-2.39982
ILMN_1816342	TBX2	17	Homo sapiens T-box 2 (TBX2), mRNA.	FLJ10169	0.00058685	-2.40063
ILMN_1683112	PEA15	1	Homo sapiens phosphoprotein enriched in astrocytes 15 (PEA15), mRNA.	HUMMAT1H; PEA-15; PED	2.04E-05	-2.40495
ILMN_2297765	DOCK2	5	Homo sapiens dedicator of cytokinesis 2 (DOCK2), mRNA.	FLJ46592; KIAA0209	2.06E-05	-2.40899
ILMN_1786310	TMEM38B	9	Homo sapiens transmembrane protein 38B (TMEM38B), mRNA.	RP11-219P18.1; FLJ1049	0.0330442	-2.41
ILMN_1784870	CLIC1	6	Homo sapiens chloride intracellular channel 1 (CLIC1), mRNA.	NCC27; G6	0.00618978	-2.41083
ILMN_1710070	CPB2	13	Homo sapiens carboxypeptidase B2 (plasma, carboxypeptidase U) (CPB2), transcript variant 1, mRNA.	CPU; PCPB; TAFI	0.00183292	-2.41134
ILMN_1726138	TCF4	18	Homo sapiens transcription factor 4 (TCF4), transcript variant 2, mRNA.	SEF2; ITF2; MGC149723;	2.87E-05	-2.4137
ILMN_1680626	ANKRD55	5	Homo sapiens ankyrin repeat domain 55 (ANKRD55), transcript variant 2, mRNA.	FLJ11795; MGC126014; I	0.00123127	-2.41578
ILMN_1863994	IL2RB	22	Homo sapiens interleukin 2 receptor, beta (IL2RB), mRNA.	CD122; P70-75	0.0109392	-2.42037

ILMN_1785570	RAGE	14	Homo sapiens renal tumor antigen (RAGE), mRNA.	MOK; RAGE1	0.00811938	-2.42496
ILMN_1771538	HNT	11	Homo sapiens neurotrimin (HNT), transcript variant 2, mRNA.	MGC60329; NTM	8.74E-06	-2.42597
ILMN_1821531	P8	16	Homo sapiens p8 protein (candidate of metastasis 1) (P8), mRNA.	COM1	0.000367081	-2.4304
ILMN_1892548	NR4A3	9	Homo sapiens nuclear receptor subfamily 4, group A, member 3 (NR4A3), transcript variant 4, mRNA.	CHN; CSMF; TEC; NOR1;	0.00446072	-2.43294
ILMN_1738604	RRAS	19	Homo sapiens related RAS viral (r-ras) oncogene homolog (RRAS), mRNA.		0.025116	-2.43523
ILMN_1763196	TSPAN4	11	Homo sapiens tetraspanin 4 (TSPAN4), transcript variant 3, mRNA.	NAG-2; NAG2; TETRASPA	0.0408667	-2.43856
ILMN_1694780	DNAJC12	10	Homo sapiens DnaJ (Hsp40) homolog, subfamily C, member 12 (DNAJC12), transcript variant 2, mRNA.	RP11-57G10.2; JDP1	0.00175945	-2.43903
ILMN_1657495	PLOD2	3	Homo sapiens procollagen-lysine, 2-oxoglutarate 5-dioxygenase 2 (PLOD2), transcript variant 2, mRNA.	LH2; TLH	0.000796256	-2.44146
ILMN_2366473	STAT1	2	Homo sapiens signal transducer and activator of transcription 1, 91kDa (STAT1), transcript variant alpha, mRNA.	ISGF-3; STAT91; DKFZp6	5.71E-07	-2.4417
ILMN_1674394	HBEGF	5	Homo sapiens heparin-binding EGF-like growth factor (HBEGF), mRNA.	DTS; DTSF; HEGFL; DTR	0.000154212	-2.44537
ILMN_1774336	DDIT4	10	Homo sapiens DNA-damage-inducible transcript 4 (DDIT4), mRNA.	RTP801; Dig2; REDD1; FI	0.000565341	-2.44557

ILMN_2189027	LOC613037	16	Homo sapiens nuclear pore complex interacting protein pseudogene (LOC613037) on chromosome 16.		0.000415197	-2.44585
ILMN_1774110	GPHN	14	Homo sapiens gephyrin (GPHN), transcript variant 1, mRNA.	GPHRYN; GPH; GEPH; KIA	0.0165352	-2.44627
ILMN_1738027	C1orf24	1	Homo sapiens chromosome 1 open reading frame 24 (C1orf24), transcript variant 2, mRNA.	NIBAN	3.09E-05	-2.44711
ILMN_1671387	TMEM53	1	Homo sapiens transmembrane protein 53 (TMEM53), mRNA.	FLJ22353; RP4-678E16.2	0.0190204	-2.4484
ILMN_1872032	CECR1	22	Homo sapiens cat eye syndrome chromosome region, candidate 1 (CECR1), transcript variant 2, mRNA.	ADGF; IDGFL	0.0310529	-2.4495
ILMN_1803033	FLJ20160	2	Homo sapiens FLJ20160 protein (FLJ20160), mRNA.		0.000109345	-2.45224
ILMN_2057768	CEP135	4	Homo sapiens centrosomal protein 135kDa (CEP135), mRNA.	KIAA0635; CEP4	4.94E-05	-2.45376
ILMN_1812700	REC8	14	Homo sapiens REC8 homolog (yeast) (REC8), transcript variant 1, mRNA.	HR21spB; REC8; MGC950	0.0252825	-2.45504
ILMN_1766650	HSD17B7P2	10	Homo sapiens hydroxysteroid (17-beta) dehydrogenase 7 pseudogene 2 (HSD17B7P2) on chromosome 10.	Hsd17b_2; HSD17B7; MG	4.14E-05	-2.45673
ILMN_2144088	CD83	6	Homo sapiens CD83 molecule (CD83), transcript variant 1, mRNA.	HB15; BL11	0.00459068	-2.4639
ILMN_2334359	DKFZP564O	4	Homo sapiens DKFZP564O0823 protein (DKFZP564O0823), mRNA.		0.0021205	-2.46823
ILMN_1655521	DBI	2	Homo sapiens diazepam binding inhibitor (GABA receptor modulator, acyl-Coenzyme A binding protein) (DBI), mRNA.	MGC70414; ACBD1; ACBI	0.000602557	-2.47014

ILMN_1804662	ABCC6	16	Homo sapiens ATP-binding cassette, sub-family C (CFTR/MRP), member 6 (ABCC6), transcript variant 2, mRNA.	MOATE; EST349056; ARA	0.0233879	-2.4734
ILMN_1749213	IGFBP1	7	Homo sapiens insulin-like growth factor binding protein 1 (IGFBP1), transcript variant 2, mRNA.	PP12; hIGFBP-1; IGF-BP2	0.00190283	-2.47491
ILMN_1729417	HLA-A	6	Homo sapiens major histocompatibility complex, class I, A (HLA-A), mRNA.		0.000310759	-2.47768
ILMN_1726306	FJX1	11	Homo sapiens four jointed box 1 (Drosophila) (FJX1), mRNA.	FLJ25593; FLJ22416	0.00129245	-2.47847
ILMN_1706523	NOD2	16	Homo sapiens nucleotide-binding oligomerization domain containing 2 (NOD2), mRNA.	NLRC2; CD; CARD15; PS(	0.000502599	-2.48125
ILMN_1655549	ACSL1	4	Homo sapiens acyl-CoA synthetase long-chain family member 1 (ACSL1), mRNA.	FACL2; LACS; FACL1; AC	0.000327941	-2.48593
ILMN_1685678	INSIG1	7	Homo sapiens insulin induced gene 1 (INSIG1), transcript variant 2, mRNA.	MGC1405; CL-6	0.000535161	-2.48928
ILMN_1765912	SNTB1	8	Homo sapiens syntrophin, beta 1 (dystrophin-associated protein A1, 59kDa, basic component 1) (SNTB1), mRNA.	A1B; DAPA1B; BSYN2; M(	0.004425	-2.49042
ILMN_1701603	FIT1	14	Homo sapiens fat-inducing transcript 1 (FIT1), mRNA.	MGC46490	0.00623114	-2.50009
ILMN_1693891	SPG3A	14	Homo sapiens spastic paraplegia 3A (autosomal dominant) (SPG3A), transcript variant 1, mRNA.	SPG3; GBP3; FSP1; AD-F	0.00283235	-2.50223
ILMN_1653821	FIS	5	Homo sapiens FIS (FIS), mRNA.	MGC126626	1.70E-05	-2.50356
ILMN_1729563	EMP3	19	Homo sapiens epithelial membrane protein 3 (EMP3), mRNA.	YMP	0.0204778	-2.50382

ILMN_1852022	HLA-DOA	6	Homo sapiens major histocompatibility complex, class II, DO alpha (HLA-DOA), mRNA.	HLA-DNA; HLADZ; HLA-D	0.00741677	-2.50477
ILMN_2227968	DEFA1	8	Homo sapiens defensin, alpha 1 (DEFA1), mRNA.	MGC138393; MRS; DEF1;	0.0183103	-2.50702
ILMN_1681780	HIST1H4C	6	Homo sapiens histone cluster 1, H4c (HIST1H4C), mRNA.	H4FG; dJ221C16.1; H4/g	0.0200245	-2.50884
ILMN_1760087	ACSM2B	16	Homo sapiens acyl-CoA synthetase medium-chain family member 2B (ACSM2B), nuclear gene encoding mitochondrial protein, mRNA.	HYST1046; HXMA; ACSM	0.0115371	-2.51439
ILMN_1787275	HLA-DRA	6	Homo sapiens major histocompatibility complex, class II, DR alpha (HLA-DRA), mRNA.	HLA-DRA1	0.00911163	-2.51861
ILMN_1774387	SCD5	4	Homo sapiens stearoyl-CoA desaturase 5 (SCD5), transcript variant 1, mRNA.	HSCD5; SCD4; FLJ21032	1.32E-05	-2.5189
ILMN_1854580	ACOT2	14	Homo sapiens acyl-CoA thioesterase 2 (ACOT2), nuclear gene encoding mitochondrial protein, mRNA.	Mte1; ZAP128; PTE2	6.10E-06	-2.52215
ILMN_2218935			Homo sapiens mRNA; cDNA DKFZp779M2422 (from clone DKFZp779M2422)		0.00597193	-2.52362
ILMN_1670903	GEMIN6	2	Homo sapiens gem (nuclear organelle) associated protein 6 (GEMIN6), mRNA.	FLJ23459	0.000789507	-2.52367
ILMN_2275248	ATP1B3		PREDICTED: Homo sapiens ATPase, Na+/K+ transporting, beta 3 polypeptide, transcript variant 2 (ATP1B3), mRNA.		0.0202856	-2.52754
ILMN_1660413	MANEA	6	Homo sapiens mannosidase, endo-alpha (MANEA), mRNA.	FLJ12838; hEndo; DKFZp	0.00541995	-2.53586
ILMN_2412927	GOLGA8B	15	Homo sapiens golgi autoantigen, golgin subfamily a, 8B (GOLGA8B), mRNA.	KIAA0855; GOLGA5	8.11E-05	-2.53591

ILMN_1695530	SLC35B1	17	Homo sapiens solute carrier family 35, member B1 (SLC35B1), mRNA.	UGTREL1	0.00255075	-2.53635
ILMN_1753111	DDEF1	8	Homo sapiens development and differentiation enhancing factor 1 (DDEF1), mRNA.	ZG14P; AMAP1; PAG2; AS	0.0110376	-2.5387
ILMN_1814589	TTLL3	3	Homo sapiens tubulin tyrosine ligase-like family, member 3 (TTLL3), transcript variant 2, mRNA.	MGC120532; MGC12053C	0.000211042	-2.53994
ILMN_1659843	STX3	11	Homo sapiens syntaxin 3 (STX3), mRNA.	STX3A	5.33E-05	-2.54263
ILMN_1752802	CRISPLD2	16	Homo sapiens cysteine-rich secretory protein LCCL domain containing 2 (CRISPLD2), mRNA.	DKFZP434B044; MGC748	0.00363816	-2.54395
ILMN_1654065	C20orf121	20	Homo sapiens chromosome 20 open reading frame 121 (C20orf121), transcript variant 2, mRNA.	DKFZp686E0870; MGC24	0.00801805	-2.54788
ILMN_1652431	SAA1	11	Homo sapiens serum amyloid A1 (SAA1), transcript variant 2, mRNA.	MGC111216; SAA; PIG4;	0.00356815	-2.54934
ILMN_1873621	PVALB	22	Homo sapiens parvalbumin (PVALB), mRNA.	D22S749; MGC116759	0.0153233	-2.55012
ILMN_1777976	DNAH2	17	Homo sapiens dynein, axonemal, heavy chain 2 (DNAH2), mRNA.	FLJ46675; DNAHC2; DNH	0.0111435	-2.55088
ILMN_2394296	PTGDS	9	Homo sapiens prostaglandin D2 synthase 21kDa (brain) (PTGDS), mRNA.	PGD2; PGDS; PGDS2; PD	0.000256625	-2.55658
ILMN_1748707	CREB5	7	Homo sapiens cAMP responsive element binding protein 5 (CREB5), transcript variant 1, mRNA.	CRE-BPA	0.00204682	-2.5588
ILMN_1762713	CLPTM1L	5	Homo sapiens CLPTM1-like (CLPTM1L), mRNA.	DKFZp666M1010; FLJ144	0.00389199	-2.56152

ILMN_1682737	RUNX1	21	Homo sapiens runt-related transcription factor 1 (acute myeloid leukemia 1; aml1 oncogene) (RUNX1), transcript variant 1, mRNA.	AMLCR1; PEBP2aB; EVI-1	0.000129068	-2.56401
ILMN_1768577	TPD52L1	6	Homo sapiens tumor protein D52-like 1 (TPD52L1), transcript variant 4, mRNA.	D53; MGC8556; hD53; TI	0.00075563	-2.56567
ILMN_1813598	MBOAT5	12	Homo sapiens membrane bound O-acyltransferase domain containing 5 (MBOAT5), mRNA.	C3F; OACT5; nessy	0.000266534	-2.56632
ILMN_1807833	CNN2	19	Homo sapiens calponin 2 (CNN2), transcript variant 2, mRNA.		0.0104079	-2.56742
ILMN_1729868	ALKBH2	12	Homo sapiens alkB, alkylation repair homolog 2 (E. coli) (ALKBH2), mRNA.	ABH2; MGC90512; hABH2	0.000152726	-2.57004
ILMN_1727618	FASTKD3	5	Homo sapiens FAST kinase domains 3 (FASTKD3), mRNA.	FLJ23274; MGC5297; MG	0.0429664	-2.57326
ILMN_1772302	SLCO2A1	3	Homo sapiens solute carrier organic anion transporter family, member 2A1 (SLCO2A1), mRNA.	OATP2A1; PGT; SLC21A2	0.000129527	-2.58072
ILMN_2405305	RAB43	3	Homo sapiens RAB43, member RAS oncogene family (RAB43), mRNA.	MGC90481; RAB11B; ISY	0.00519046	-2.58628
ILMN_1657838	NR4A1	12	Homo sapiens nuclear receptor subfamily 4, group A, member 1 (NR4A1), transcript variant 3, mRNA.	N10; NUR77; NP10; NAK-	0.00597954	-2.58738
ILMN_1869781	WNT4	1	Homo sapiens wingless-type MMTV integration site family, member 4 (WNT4), mRNA.	WNT-4	6.23E-05	-2.58798
ILMN_1733758	GSTP1	11	Homo sapiens glutathione S-transferase pi (GSTP1), mRNA.	DFN7; GST3; PI; FAEES3	0.0342348	-2.64368
ILMN_1659947	KLF13	15	Homo sapiens Kruppel-like factor 13 (KLF13), mRNA.	NSLP1; FKLF2; RFLAT-1;	0.00442022	-2.64813

ILMN_1758457	PTGIS	20	Homo sapiens prostaglandin I2 (prostacyclin) synthase (PTGIS), mRNA.	CYP8A1; PGIS; MGC1268	0.0125244	-2.64894
ILMN_1846406	CYBA	16	Homo sapiens cytochrome b-245, alpha polypeptide (CYBA), mRNA.		4.53E-05	-2.6512
ILMN_1655312	ABCC9	12	Homo sapiens ATP-binding cassette, sub-family C (CFTR/MRP), member 9 (ABCC9), transcript variant SUR2B, mRNA.	ABC37; FLJ36852; SUR2;	0.00271169	-2.65548
ILMN_1671489	SAMD5	6	Homo sapiens sterile alpha motif domain containing 5 (SAMD5), mRNA.	dJ875H10.1	0.0110458	-2.65932
ILMN_2305407	ZNF763	19	Homo sapiens zinc finger protein 763 (ZNF763), mRNA.	ZNF; ZNF440L	0.00965701	-2.66037
ILMN_1771051	ITGAV	2	Homo sapiens integrin, alpha V (vitronectin receptor, alpha polypeptide, antigen CD51) (ITGAV), mRNA.	CD51; DKFZp686A08142	0.0127168	-2.66197
ILMN_1670672	TRPM4	19	Homo sapiens transient receptor potential cation channel, subfamily M, member 4 (TRPM4), mRNA.	FLJ20041; TRPM4B	0.00448067	-2.66439
ILMN_1677962	VIM	10	Homo sapiens vimentin (VIM), mRNA.	FLJ36605	9.76E-06	-2.66492
ILMN_2347789	PDCD4	10	Homo sapiens programmed cell death 4 (neoplastic transformation inhibitor) (PDCD4), transcript variant 2, mRNA.	MGC33046; MGC33047; I	0.00162217	-2.66526
ILMN_1702698	TCEAL2	X	Homo sapiens transcription elongation factor A (SII)-like 2 (TCEAL2), mRNA.	MY0876G05; my048	2.62E-06	-2.67227
ILMN_1706818	TNFAIP2	14	Homo sapiens tumor necrosis factor, alpha-induced protein 2 (TNFAIP2), mRNA.	B94	0.00318423	-2.67352
ILMN_1805104	JUND	19	Homo sapiens jun D proto-oncogene (JUND), mRNA.		0.000179989	-2.67825

ILMN_1720889	RND2	17	Homo sapiens Rho family GTPase 2 (RND2), mRNA.	ARHN; RHO7; RhoN	3.55E-05	-2.68581
ILMN_1697802	LOC158160	10	Homo sapiens hypothetical protein LOC158160 (LOC158160), transcript variant 1, mRNA.	Hsd17b_2; HSD17B7; MG	0.000157398	-2.68819
ILMN_2303166	DEFA4	8	Homo sapiens defensin, alpha 4, corticostatin (DEFA4), mRNA.	MGC120099; HNP-4; MG(	4.20E-06	-2.70499
ILMN_1734702	HMBS	11	Homo sapiens hydroxymethylbilane synthase (HMBS), transcript variant 1, mRNA.	PBG-D; UPS; PBGD	2.61E-06	-2.70905
ILMN_1780575	LUM	12	Homo sapiens lumican (LUM), mRNA.	SLRR2D; LDC	0.00234988	-2.71203
ILMN_2094061	LCAT	16	Homo sapiens lecithin-cholesterol acyltransferase (LCAT), mRNA.		0.00211079	-2.71271
ILMN_1767831	PCBD1	10	Homo sapiens pterin-4 alpha-carbinolamine dehydratase/dimerization cofactor of hepatocyte nuclear factor 1 alpha (PCBD1), mRNA.	PHS; PCD; DCOH; PCBD	0.000545826	-2.71583
ILMN_1815527	GLS	2	Homo sapiens glutaminase (GLS), mRNA.	KIAA0838; GLS1; DKFZp(	0.0075979	-2.7208
ILMN_2411731	TRIM15	6	Homo sapiens tripartite motif-containing 15 (TRIM15), transcript variant 1, mRNA.	ZNF178; ZNFB7; RNF93	0.00239282	-2.72652
ILMN_1738866	DPYSL2	8	Homo sapiens dihydropyrimidinase-like 2 (DPYSL2), mRNA.	DHPRP2; DRP-2; DRP2; C	2.79E-06	-2.72701
ILMN_1689037			AV681673 GKB Homo sapiens cDNA clone GKBABD06 5, mRNA sequence		0.000146645	-2.72906
ILMN_1737517	DKK3	11	Homo sapiens dickkopf homolog 3 (Xenopus laevis) (DKK3), transcript variant 1, mRNA.	REIC	0.00899632	-2.73433

ILMN_1905548	PRRX1	1	Homo sapiens paired related homeobox 1 (PRRX1), transcript variant pmx-1a, mRNA.	PRX1; PMX1; PHOX1	0.0203703	-2.74469
ILMN_2340347	HCST	19	Homo sapiens hematopoietic cell signal transducer (HCST), transcript variant 2, mRNA.	DKFZP586C1522; DAP10	0.0106186	-2.7453
ILMN_1757026	EGR2	10	Homo sapiens early growth response 2 (Krox-20 homolog, Drosophila) (EGR2), mRNA.	KROX20; FLJ14547; DKFZ	2.20E-05	-2.75209
ILMN_1751453	HK1	10	Homo sapiens hexokinase 1 (HK1), nuclear gene encoding mitochondrial protein, transcript variant 5, mRNA.	HK1-tc; HKI; HK1-tb; HK	5.28E-07	-2.755
ILMN_1663032	FNDC4	2	Homo sapiens fibronectin type III domain containing 4 (FNDC4), mRNA.	FLJ22362; FRCP1	3.33E-05	-2.75527
ILMN_2312817	LOC732058		PREDICTED: Homo sapiens similar to Apolipoprotein(a) precursor (Apo(a)) (Lp(a)) (LOC732058), mRNA.		0.0299482	-2.75797
ILMN_1854270	ISOC2	19	Homo sapiens isochorismatase domain containing 2 (ISOC2), mRNA.		0.000553768	-2.76417
ILMN_1733443	PKM2	15	Homo sapiens pyruvate kinase, muscle (PKM2), transcript variant 3, mRNA.	PKM; THBP1; MGC3932; (	3.20E-05	-2.77083
ILMN_1782938	FXYD5	19	Homo sapiens FXYD domain containing ion transport regulator 5 (FXYD5), transcript variant 2, mRNA.	dysad; OIT2; PRO6241; H	0.00732388	-2.77407
ILMN_1676384	DNAJC12	10	Homo sapiens DnaJ (Hsp40) homolog, subfamily C, member 12 (DNAJC12), transcript variant 1, mRNA.	RP11-57G10.2; JDP1	0.00031581	-2.77512
ILMN_1697872	BRCA1	17	Homo sapiens breast cancer 1, early onset (BRCA1), transcript variant BRCA1-delta11b, mRNA.	RNF53; BRCAI; PSCP; BR	0.00239417	-2.77542

ILMN_1708672	SHFM1	7	Homo sapiens split hand/foot malformation (ectrodactyly) type 1 (SHFM1), mRNA.	SHSF1; DSS1; SHFD1; SI	0.024509	-2.77847
ILMN_1688580	GMPPB	3	Homo sapiens GDP-mannose pyrophosphorylase B (GMPPB), transcript variant 2, mRNA.	KIAA1851	0.0327913	-2.78399
ILMN_1813456			Homo sapiens mRNA; cDNA DKFZp686F1546 (from clone DKFZp686F1546)		0.000142054	-2.79654
ILMN_1757867	SERPINA1	14	Homo sapiens serpin peptidase inhibitor, clade A (alpha-1 antiproteinase, antitrypsin), member 1 (SERPINA1), transcript variant 1, mRNA.	PI1; MGC23330; PRO227	0.000858454	-2.80046
ILMN_2125395	FPR3	19	Homo sapiens formyl peptide receptor 3 (FPR3), mRNA.	FMLPY; RMLP-R-I; FPRH2	0.003247	-2.81478
ILMN_1758938	HLA-DPA1	6	Homo sapiens major histocompatibility complex, class II, DP alpha 1 (HLA-DPA1), mRNA.	HLADP; HLA-DP1A; HLAS	3.58E-06	-2.81487
ILMN_1741957	NDUFAF1	15	Homo sapiens NADH dehydrogenase (ubiquinone) 1 alpha subcomplex, assembly factor 1 (NDUFAF1), mRNA.	CGI65; CGI-65; CIA30	0.00861706	-2.82947
ILMN_1714383	ECE2	3	Homo sapiens endothelin converting enzyme 2 (ECE2), transcript variant 3, mRNA.	MGC78487; MGC17664; I	3.54E-05	-2.83227
ILMN_1764980	PRICKLE1	12	Homo sapiens prickle homolog 1 (Drosophila) (PRICKLE1), mRNA.	MGC138902; FLJ31627; I	0.00125012	-2.83782
ILMN_2141790	ITGB2	21	Homo sapiens integrin, beta 2 (antigen CD18 (p95), lymphocyte function-associated antigen 1; macrophage antigen 1 (mac-1) beta subunit) (ITGB2), mRNA.	MAC-1; LFA-1; MF17; LC/	0.00641559	-2.83987

ILMN_1677607	OSR1	2	Homo sapiens odd-skipped related 1 (Drosophila) (OSR1), mRNA.	ODD	1.96E-05	-2.84288
ILMN_1783963	SLC46A1	17	Homo sapiens solute carrier family 46 (folate transporter), member 1 (SLC46A1), mRNA.	MGC9564; HCP1; PCFT	0.00412883	-2.86546
ILMN_1811933	PPP1R1A	12	Homo sapiens protein phosphatase 1, regulatory (inhibitor) subunit 1A (PPP1R1A), mRNA.		3.95E-05	-2.86596
ILMN_1762883	ZYX	7	Homo sapiens zyxin (ZYX), transcript variant 1, mRNA.	ESP-2; HED-2	0.00065008	-2.87454
ILMN_1774604	TMEM200A	6	Homo sapiens transmembrane protein 200A (TMEM200A), mRNA.	TTMC	0.0305297	-2.88258
ILMN_2323338	LOC606724	16	Homo sapiens coronin, actin binding protein, 1A pseudogene (LOC606724) on chromosome 16.		2.85E-05	-2.8864
ILMN_1655244	ATF7IP	12	Homo sapiens activating transcription factor 7 interacting protein (ATF7IP), mRNA.	FLJ10139; FLJ10688; MC/	1.94E-05	-2.88908
ILMN_1669940	CDC42EP5	19	Homo sapiens CDC42 effector protein (Rho GTPase binding) 5 (CDC42EP5), mRNA.	MGC21945; CEP5; Borg3	0.00232556	-2.8954
ILMN_2043918	DPAGT1	11	Homo sapiens dolichyl-phosphate (UDP-N-acetylglucosamine) N-acetylglucosaminephosphotransferase 1 (GlcNAc-1-P transferase) (DPAGT1), transcript variant 1, mRNA.	GPT; DPAGT2; D11S366;	1.31E-05	-2.8962
ILMN_1729801	SLITRK3	3	Homo sapiens SLIT and NTRK-like family, member 3 (SLITRK3), mRNA.	MGC138681; KIAA0848	0.011105	-2.89938
ILMN_1684017	STEAP3	2	Homo sapiens STEAP family member 3 (STEAP3), transcript variant 2, mRNA.	STMP3; dudlin-2; TSAP6	5.78E-05	-2.90465

ILMN_1707551	S100A12	1	Homo sapiens S100 calcium binding protein A12 (S100A12), mRNA.	CAAF1; CAGC; ENRAGE;	5.59E-05	-2.91311
ILMN_1773586	FRAS1	4	Homo sapiens Fraser syndrome 1 (FRAS1), mRNA.	KIAA1500; FLJ22031; FLJ	0.00168354	-2.92328
ILMN_1693939	FILIP1L	3	Homo sapiens filamin A interacting protein 1-like (FILIP1L), transcript variant 1, mRNA.	DOC-1; GIP90; DOC1	0.0272185	-2.92467
ILMN_1726659	MFSD10	4	Homo sapiens major facilitator superfamily domain containing 10 (MFSD10), mRNA.	TETTRAN; TPO1	0.0268558	-2.92834
ILMN_1729212	CX3CR1	3	Homo sapiens chemokine (C-X3-C motif) receptor 1 (CX3CR1), mRNA.	CMKDR1; GPR13; V28; G	0.000295879	-2.94477
ILMN_1803005	STON1	2	Homo sapiens stonin 1 (STON1), mRNA.	DKFZp781K2462; SBLF; S	3.66E-07	-2.94613
ILMN_2228463	SH3GL2	9	Homo sapiens SH3-domain GRB2-like 2 (SH3GL2), mRNA.	CNSA2; SH3P4; SH3D2A;	0.00534572	-2.95001
ILMN_1755926			Homo sapiens mRNA; cDNA DKFZp686D0853 (from clone DKFZp686D0853)		9.74E-07	-2.9567
ILMN_1881206	HRC	19	Homo sapiens histidine rich calcium binding protein (HRC), mRNA.	MGC133236	0.002463	-2.98087
ILMN_1844400	ARHGEF3	3	Homo sapiens Rho guanine nucleotide exchange factor (GEF) 3 (ARHGEF3), mRNA.	DKFZP434F2429; XPLN; (	1.22E-05	-2.98567
ILMN_1716435	COL16A1	1	Homo sapiens collagen, type XVI, alpha 1 (COL16A1), mRNA.	FP1572; 447AA	2.86E-06	-2.98752
ILMN_1715024	KLF4	9	Homo sapiens Kruppel-like factor 4 (gut) (KLF4), mRNA.	GKLF; EZF	0.0325347	-2.99519
ILMN_1793410	EDNRA	4	Homo sapiens endothelin receptor type A (EDNRA), mRNA.	ETRA; ETA	0.00166149	-3.00218
ILMN_1720858	ANXA1	9	Homo sapiens annexin A1 (ANXA1), mRNA.	LPC1; ANX1	0.000172166	-3.00529

ILMN_1681916	RAB7B		Homo sapiens RAB7B, member RAS oncogene family (RAB7B), mRNA.	MGC9726; RAB7; MGC16	1.51E-05	-3.01136
ILMN_1749417	TIMP2	17	Homo sapiens TIMP metallopeptidase inhibitor 2 (TIMP2), mRNA.	CSC-21K	0.00581268	-3.03176
ILMN_1709167	FLJ35258	19	Homo sapiens hypothetical protein 284297 (FLJ35258), mRNA.		0.000100002	-3.04271
ILMN_1797154	HLA-DRA	6	Homo sapiens major histocompatibility complex, class II, DR alpha (HLA-DRA), mRNA.	HLA-DRA1	0.000428098	-3.04403
ILMN_2172091	RGS10	10	Homo sapiens regulator of G-protein signaling 10 (RGS10), transcript variant 2, mRNA.		0.00878113	-3.04552
ILMN_1658917	ERBB2	17	Homo sapiens v-erb-b2 erythroblastic leukemia viral oncogene homolog 2, neuro/glioblastoma derived oncogene homolog (avian) (ERBB2), transcript variant 1, mRNA.	c-erb B2; CD340; HER2;	0.000223274	-3.05021
ILMN_1700340	ACTA2	10	Homo sapiens actin, alpha 2, smooth muscle, aorta (ACTA2), mRNA.	ACTSA	0.00186675	-3.05253
ILMN_2381206	GALK1	17	Homo sapiens galactokinase 1 (GALK1), mRNA.	GALK; GK1	0.00130688	-3.05348
ILMN_1738268	CLSTN1	1	Homo sapiens calsyntenin 1 (CLSTN1), transcript variant 1, mRNA.	CSTN1; PIK3CD; XB31alp	0.00349966	-3.05951
ILMN_1744442	RGS2	1	Homo sapiens regulator of G-protein signalling 2, 24kDa (RGS2), mRNA.	G0S8	4.17E-07	-3.06396
ILMN_1662640	PPP1R3C	10	Homo sapiens protein phosphatase 1, regulatory (inhibitor) subunit 3C (PPP1R3C), mRNA.	PPP1R5	0.00197623	-3.07539

ILMN_2402817	OAS2	12	Homo sapiens 2'-5'-oligoadenylate synthetase 2, 69/71kDa (OAS2), transcript variant 1, mRNA.	MGC78578	0.00248	-3.07577
ILMN_1659682	GLRX	5	Homo sapiens glutaredoxin (thioltransferase) (GLRX), mRNA.	GRX; GRX1; MGC117407	3.15E-05	-3.08833
ILMN_1786426	LAMA2	6	Homo sapiens laminin, alpha 2 (merosin, congenital muscular dystrophy) (LAMA2), transcript variant 2, mRNA.	LAMM	0.000119428	-3.12243
ILMN_2056606	SYNPO2		PREDICTED: Homo sapiens synaptopodin 2, transcript variant 4 (SYNPO2), mRNA.		0.0164068	-3.12254
ILMN_1676192	LRRC32	11	Homo sapiens leucine rich repeat containing 32 (LRRC32), mRNA.	GARP; D11S833E	0.016716	-3.14644
ILMN_1758673	FOSB	19	Homo sapiens FBJ murine osteosarcoma viral oncogene homolog B (FOSB), mRNA.	GOS3; G0S3; MGC42291	1.86E-06	-3.15382
ILMN_1767474	MAPK10	4	Homo sapiens mitogen-activated protein kinase 10 (MAPK10), transcript variant 2, mRNA.	PRKM10; JNK3; FLJ33785	7.92E-06	-3.15505
ILMN_1772131	SPARCL1	4	Homo sapiens SPARC-like 1 (mast9, hevin) (SPARCL1), mRNA.	PIG33; SC1	2.79E-08	-3.1764
ILMN_1673649	FGG	4	Homo sapiens fibrinogen gamma chain (FGG), transcript variant gamma-B, mRNA.		0.00288971	-3.17646
ILMN_1761312	SLC1A7	1	Homo sapiens solute carrier family 1 (glutamate transporter), member 7 (SLC1A7), mRNA.	FLJ36602; EAAT5; AAAT	0.0130362	-3.17995
ILMN_1743784	SGIP1	1	Homo sapiens SH3-domain GRB2-like (endophilin) interacting protein 1 (SGIP1), mRNA.	FLJ43054; DKFZp761D22	0.000250199	-3.18593

ILMN_1808078	EVI1	3	Homo sapiens ecotropic viral integration site 1 (EVI1), mRNA.	EVI-1; PRDM3; MGC1633	0.000473497	-3.18654
ILMN_1730628	SLCO3A1		PREDICTED: Homo sapiens solute carrier organic anion transporter family, member 3A1, transcript variant 2 (SLCO3A1), mRNA.		0.00047471	-3.20051
ILMN_2319424	HSH2D	19	Homo sapiens hematopoietic SH2 domain containing (HSH2D), mRNA.	HSH2; ALX; FLJ14886	0.000202556	-3.20282
ILMN_1654385	LSM11	5	Homo sapiens LSM11, U7 small nuclear RNA associated (LSM11), mRNA.	FLJ38273	3.15E-05	-3.20324
ILMN_2364131	SYT17	16	Homo sapiens synaptotagmin XVII (SYT17), mRNA.		2.09E-07	-3.2176
ILMN_2136089	MLLT6	17	Homo sapiens myeloid/lymphoid or mixed-lineage leukemia (trithorax homolog, Drosophila); translocated to, 6 (MLLT6), mRNA.	AF17; FLJ23480	0.0005839	-3.22175
ILMN_1703572	MGC61598		PREDICTED: Homo sapiens similar to ankyrin-repeat protein Nrarp (MGC61598), mRNA.		0.0224985	-3.22256
ILMN_1763852			Homo sapiens cDNA FLJ26188 fis, clone ADG04821		1.67E-06	-3.23519
ILMN_1801020	GLIS3	9	Homo sapiens GLIS family zinc finger 3 (GLIS3), transcript variant 2, mRNA.	MGC33662; FLJ90578; ZN	0.000118315	-3.23612
ILMN_1672872			EST366269 MAGE resequences, MAGC Homo sapiens cDNA, mRNA sequence		0.000201662	-3.27354

ILMN_1778444	KCNN2	5	Homo sapiens potassium intermediate/small conductance calcium-activated channel, subfamily N, member 2 (KCNN2), transcript variant 1, mRNA.	KCa2.2; SKCA2; SK2; hS	0.000969828	-3.27475
ILMN_1782993	RASL12	15	Homo sapiens RAS-like, family 12 (RASL12), mRNA.	RIS	0.00050884	-3.32346
ILMN_1705629	BGN	X	Homo sapiens biglycan (BGN), mRNA.	SLRR1A; PG-S1; PGI; DS	0.000103703	-3.33061
ILMN_1787815	MAP1LC3A	20	Homo sapiens microtubule-associated protein 1 light chain 3 alpha (MAP1LC3A), transcript variant 2, mRNA.	MAP1ALC3; MAP1BLC3	0.00266895	-3.339
ILMN_1705183	NEU4	2	Homo sapiens sialidase 4 (NEU4), mRNA.	MGC102757; MGC18222	0.00612275	-3.3401
ILMN_1679401	PHLDB1	11	Homo sapiens pleckstrin homology-like domain, family B, member 1 (PHLDB1), mRNA.	FLJ90266; DKFZp686O24	7.12E-05	-3.34848
ILMN_1736533	LGALS1	22	Homo sapiens lectin, galactoside-binding, soluble, 1 (galectin 1) (LGALS1), mRNA.	DKFZp686E23103; GBP	0.000836784	-3.39455
ILMN_1779381	JMJD5	16	Homo sapiens jumonji domain containing 5 (JMJD5), mRNA.	FLJ13798	2.27E-08	-3.42298
ILMN_2111187	UBQLNL	11	Homo sapiens ubiquilin-like (UBQLNL), mRNA.	MGC26958; MGC20470	0.0468338	-3.43345
ILMN_1794844	PAPPA	9	Homo sapiens pregnancy-associated plasma protein A, pappalysin 1 (PAPPA), mRNA.	DIPLA1; IGFBP-4ase; ASE	0.00439112	-3.43684
ILMN_1767113	NR4A2	2	Homo sapiens nuclear receptor subfamily 4, group A, member 2 (NR4A2), transcript variant 1, mRNA.	RNR1; NOT; TINUR; NURI	0.00058276	-3.437
ILMN_1775170	DECR2	16	Homo sapiens 2,4-dienoyl CoA reductase 2, peroxisomal (DECR2), mRNA.	PDCR	7.96E-05	-3.4514
ILMN_1781560	KIAA0644	7	Homo sapiens KIAA0644 gene product (KIAA0644), mRNA.		1.25E-05	-3.45251

ILMN_1678170	NAT2	8	Homo sapiens N-acetyltransferase 2 (arylamine N-acetyltransferase) (NAT2), mRNA.	AAC2	0.0347798	-3.47938
ILMN_1757036	C21orf121	21	Homo sapiens chromosome 21 open reading frame 121 (C21orf121), mRNA.	PRED87	2.07E-05	-3.51815
ILMN_1728048	MYADM	19	Homo sapiens myeloid-associated differentiation marker (MYADM), transcript variant 4, mRNA.	SB135	0.000369643	-3.52455
ILMN_1808674	ARMCX2	X	Homo sapiens armadillo repeat containing, X-linked 2 (ARMCX2), mRNA.	ALEX2; MGC8742; MGC1	0.0106903	-3.52979
ILMN_1736238	PDIA6	2	Homo sapiens protein disulfide isomerase family A, member 6 (PDIA6), mRNA.	P5; TXNDC7; ERP5	0.0216247	-3.55324
ILMN_2178855	MKX	10	Homo sapiens mohawk homeobox (MKX), mRNA.	MGC39616; IFRX; IRXL1;	0.00151074	-3.59392
ILMN_2193817	NFE2L3	7	Homo sapiens nuclear factor (erythroid-derived 2)-like 3 (NFE2L3), mRNA.	NRF3	0.0071036	-3.59759
ILMN_1671891	HSPA5	9	Homo sapiens heat shock 70kDa protein 5 (glucose-regulated protein, 78kDa) (HSPA5), mRNA.	BIP; FLJ26106; MIF2; GR	5.14E-08	-3.60342
ILMN_2210753	PHIP	6	Homo sapiens pleckstrin homology domain interacting protein (PHIP), mRNA.	FLJ20705; FLJ45918; ndr	8.90E-09	-3.65413
ILMN_1898518	CASP1	11	Homo sapiens caspase 1, apoptosis-related cysteine peptidase (interleukin 1, beta, convertase) (CASP1), transcript variant delta, mRNA.	IL1BC; ICE; P45	0.000680987	-3.65767
ILMN_1784364	FABP4	8	Homo sapiens fatty acid binding protein 4, adipocyte (FABP4), mRNA.	A-FABP	1.68E-05	-3.66281

ILMN_2384056	LOC649923		PREDICTED: Homo sapiens similar to Ig gamma-2 chain C region (LOC649923), mRNA.		0.00781068	-3.67462
ILMN_1780060	ATOH8	2	Homo sapiens atonal homolog 8 (Drosophila) (ATOH8), mRNA.	HATH6; FLJ14708; FLJ38	0.00392482	-3.71253
ILMN_1809483	GJC2	1	Homo sapiens gap junction protein, gamma 2, 47kDa (GJC2), mRNA.	MGC105119; PMLDAR; C)	0.0217505	-3.71434
ILMN_1692163	CCDC51	3	Homo sapiens coiled-coil domain containing 51 (CCDC51), mRNA.	FLJ12436	0.00104376	-3.72207
ILMN_2166457	OLFM4	13	Homo sapiens olfactomedin 4 (OLFM4), mRNA.	bA209J19.1; OlfD; KIAA4	0.0030269	-3.73052
ILMN_1758213	SLC35F2	11	Homo sapiens solute carrier family 35, member F2 (SLC35F2), mRNA.	HSNOV1; FLJ13018; DKF	0.00379624	-3.75495
ILMN_2322131	HSD17B11	4	Homo sapiens hydroxysteroid (17-beta) dehydrogenase 11 (HSD17B11), mRNA.	17-BETA-HSDXI; DHRS8;	0.00721179	-3.75646
ILMN_1661631	TM4SF1	3	Homo sapiens transmembrane 4 L six family member 1 (TM4SF1), mRNA.	M3S1; L6; H-L6; TAAL6	0.000572675	-3.78186
ILMN_1668850	DLEU1	13	Homo sapiens deleted in lymphocytic leukemia, 1 (DLEU1) on chromosome 13.	XTP6; MGC22430; DLB1;	6.03E-05	-3.86124
ILMN_1783998	NIN	14	Homo sapiens ninein (GSK3B interacting protein) (NIN), transcript variant 2, mRNA.	KIAA1565	6.79E-06	-3.88273
ILMN_2391861	TMEM132A	11	Homo sapiens transmembrane protein 132A (TMEM132A), transcript variant 2, mRNA.	DKFZp547E212; HSPA5BI	0.0474044	-3.90635
ILMN_1762735	COLEC11	2	Homo sapiens collectin sub-family member 11 (COLEC11), transcript variant 1, mRNA.	CL-K1-II; CL-K1-IIb; DKF	0.000144661	-3.92862

ILMN_1805228	BCL2	18	Homo sapiens B-cell CLL/lymphoma 2 (BCL2), nuclear gene encoding mitochondrial protein, transcript variant alpha, mRNA.	Bcl-2	0.000587655	-3.96295
ILMN_1823221	CAMK1G	1	Homo sapiens calcium/calmodulin-dependent protein kinase IG (CAMK1G), mRNA.	VWS1; CLICKIII; dJ272L1	2.36E-05	-3.96684
ILMN_1689842	RAB34	17	Homo sapiens RAB34, member RAS oncogene family (RAB34), mRNA.	RAH; RAB39	0.00232974	-4.08962
ILMN_1782958			BX105338 Soares_pregnant_uterus_NbHPU Homo sapiens cDNA clone IMAGp998C114347, mRNA sequence		4.97E-06	-4.10371
ILMN_1793504	MTUS1	8	Homo sapiens mitochondrial tumor suppressor 1 (MTUS1), nuclear gene encoding mitochondrial protein, transcript variant 5, mRNA.	MTSG1; MP44; DKFZp586	0.000252359	-4.11322
ILMN_2346987	PPAP2C	19	Homo sapiens phosphatidic acid phosphatase type 2C (PPAP2C), transcript variant 3, mRNA.	PAP-2c; PAP2-g; LPP2	0.0274503	-4.11971
ILMN_1673522	ANGPTL2	9	Homo sapiens angiopoietin-like 2 (ANGPTL2), mRNA.	ARP2; MGC8889; HARP	0.00386576	-4.2391
ILMN_2201580	IL20RB	3	Homo sapiens interleukin 20 receptor beta (IL20RB), mRNA.	MGC34923; IL-20R2; FND	0.0440291	-4.2768
ILMN_1795298	LECT2	5	Homo sapiens leukocyte cell-derived chemotaxin 2 (LECT2), mRNA.	chm2; MGC126628; chm-	0.00163191	-4.28492
ILMN_2412880	LOC284757	20	Homo sapiens hypothetical protein LOC284757 (LOC284757), mRNA.	FLJ46426	0.000115282	-4.29771

ILMN_1663575	KCNA6	12	Homo sapiens potassium voltage-gated channel, shaker-related subfamily, member 6 (KCNA6), mRNA.	KV1.6; HBK2	0.00044298	-4.29838
ILMN_1694653	THBS2	6	Homo sapiens thrombospondin 2 (THBS2), mRNA.	TSP2	0.000498078	-4.30481
ILMN_1733094	NAMPT	7	Homo sapiens nicotinamide phosphoribosyltransferase (NAMPT), mRNA.	DKFZP666B131; PBEF; 1[illegible]	0.000149112	-4.32249
ILMN_1664398	MGC87042	7	PREDICTED: Homo sapiens similar to Six transmembrane epithelial antigen of prostate (MGC87042), mRNA.		0.0242862	-4.33532
ILMN_1700042	TGIF1	18	Homo sapiens TGFB-induced factor homeobox 1 (TGIF1), transcript variant 1, mRNA.	HPE4; MGC5066; MGC39[illegible]	0.00290211	-4.33749
ILMN_1733259	FLJ22374	7	Homo sapiens hypothetical protein FLJ22374 (FLJ22374), mRNA.	MGC44277	0.00224384	-4.43115
ILMN_2269256	COL4A3	2	Homo sapiens collagen, type IV, alpha 3 (Goodpasture antigen) (COL4A3), transcript variant 5, mRNA.		0.000366877	-4.5315
ILMN_1685636	GABRP	5	Homo sapiens gamma-aminobutyric acid (GABA) A receptor, pi (GABRP), mRNA.	MGC126386; MGC126387	0.00235414	-4.61934
ILMN_2245241	PCDH18	4	Homo sapiens protocadherin 18 (PCDH18), mRNA.	KIAA1562; DKFZP434B09[illegible]	0.0197151	-4.64574
ILMN_1736670	SPRY1	4	Homo sapiens sprouty homolog 1, antagonist of FGF signaling (Drosophila) (SPRY1), transcript variant 2, mRNA.	hSPRY1	8.94E-06	-4.71898
ILMN_1803073	THADA	2	Homo sapiens thyroid adenoma associated (THADA), transcript variant 1, mRNA.	GITA; FLJ44016; KIAA17[illegible]	9.15E-05	-4.76072
ILMN_1740402	ARMCX1	X	Homo sapiens armadillo repeat containing, X-linked 1 (ARMCX1), mRNA.	DKFZp686P06199; ALEX1	1.84E-07	-4.83003

ILMN_1808114	CCRN4L	4	Homo sapiens CCR4 carbon catabolite repression 4-like (S. cerevisiae) (CCRN4L), mRNA.	CCR4L; MGC142060; MG[illegible]	7.03E-05	-4.90518
ILMN_2311020	CD83	6	Homo sapiens CD83 molecule (CD83), transcript variant 2, mRNA.	HB15; BL11	0.000445347	-5.31349
ILMN_2357062	WDR72	15	Homo sapiens WD repeat domain 72 (WDR72), mRNA.	MGC126663; FLJ38736; [illegible]	0.0315266	-5.3182
ILMN_1764030	SDF2L1	22	Homo sapiens stromal cell-derived factor 2-like 1 (SDF2L1), mRNA.		0.000158663	-5.38473
ILMN_2116877	SDCBP2	20	Homo sapiens syndecan binding protein (syntenin) 2 (SDCBP2), transcript variant 1, mRNA.	SITAC18; FLJ12256; ST-[illegible]	0.0177375	-5.78984
ILMN_1671808	TPM2	9	Homo sapiens tropomyosin 2 (beta) (TPM2), transcript variant 2, mRNA.	AMCD1; TMSB; DA1	2.73E-11	-5.80317
ILMN_1748899	FAM129B	9	Homo sapiens family with sequence similarity 129, member B (FAM129B), transcript variant 2, mRNA.	OC58; MEG-3; FLJ13518;	0.00885225	-5.88195
ILMN_1868150	TMEM158	3	Homo sapiens transmembrane protein 158 (TMEM158), mRNA.	BBP; DKFZp586E1621; R[illegible]	0.00355357	-5.9004
ILMN_2349771	TIAM2	6	Homo sapiens T-cell lymphoma invasion and metastasis 2 (TIAM2), transcript variant 2, mRNA.	STEF; FLJ41865	0.000762072	-5.91422
ILMN_1807464	TPM4	19	Homo sapiens tropomyosin 4 (TPM4), mRNA.		0.0454445	-5.91915
ILMN_1811624	ANKDD1A	15	Homo sapiens ankyrin repeat and death domain containing 1A (ANKDD1A), mRNA.	MGC120306; FLJ25870; [illegible]	0.014065	-5.9711
ILMN_1729188	S100A13	1	Homo sapiens S100 calcium binding protein A13 (S100A13), transcript variant 3, mRNA.		0.0011206	-6.01114

ILMN_1706635	PLCB2	15	Homo sapiens phospholipase C, beta 2 (PLCB2), mRNA.	FLJ38135	0.00358464	-6.03177
ILMN_1668134	DKFZP586H	11	Homo sapiens regeneration associated muscle protease (DKFZP586H2123), transcript variant 1, mRNA.	RAMP; FP938	0.0257746	-6.06679
ILMN_1683923	FILIP1L	3	Homo sapiens filamin A interacting protein 1-like (FILIP1L), transcript variant 3, mRNA.	DOC-1; GIP90; DOC1	0.00192261	-6.19801
ILMN_1736969	TMEM173	5	Homo sapiens transmembrane protein 173 (TMEM173), mRNA.	FLJ38577	0.00110986	-6.27523
ILMN_1693262	ABR	17	Homo sapiens active BCR-related gene (ABR), transcript variant 2, mRNA.	MDB; FLJ45954	0.0134141	-6.27942
ILMN_1745103	KIAA1407	3	Homo sapiens KIAA1407 (KIAA1407), mRNA.	FLJ43314	0.000669593	-6.36888
ILMN_1686109	FBLN2	3	Homo sapiens fibulin 2 (FBLN2), transcript variant 2, mRNA.		7.63E-05	-6.46902
ILMN_1876379	NFKB2	10	Homo sapiens nuclear factor of kappa light polypeptide gene enhancer in B-cells 2 (p49/p100) (NFKB2), transcript variant 2, mRNA.	LYT10; LYT-10	5.90E-07	-6.53982
ILMN_1762255	SLC16A10	6	Homo sapiens solute carrier family 16, member 10 (aromatic amino acid transporter) (SLC16A10), mRNA.	TAT1; PRO0813	0.0252762	-6.69993
ILMN_1715401	LZTS1	8	Homo sapiens leucine zipper, putative tumor suppressor 1 (LZTS1), mRNA.	FEZ1; F37	0.00036851	-6.79051
ILMN_1693941	DPYSL3	5	Homo sapiens dihydropyrimidinase-like 3 (DPYSL3), mRNA.	DRP3; CRMP4; ULIP; DRF	0.00185772	-6.83195

ILMN_1718766	PCBD1	10	Homo sapiens pterin-4 alpha-carbinolamine dehydratase/dimerization cofactor of hepatocyte nuclear factor 1 alpha (PCBD1), mRNA.	PHS; PCD; DCOH; PCBD	0.000339714	-6.8412
ILMN_1718552	MNS1	15	Homo sapiens meiosis-specific nuclear structural 1 (MNS1), mRNA.	FLJ11222	0.0494539	-9.02519
ILMN_2304512	OSTalpha	3	Homo sapiens organic solute transporter alpha (OSTalpha), mRNA.	MGC39807	2.02E-07	-9.31449
ILMN_1753347	CAPNS1	19	Homo sapiens calpain, small subunit 1 (CAPNS1), transcript variant 2, mRNA.	CANP; CALPAIN4; CDPS;	0.0191218	-9.7398
ILMN_1764557	SPRY1	4	Homo sapiens sprouty homolog 1, antagonist of FGF signaling (Drosophila) (SPRY1), transcript variant 1, mRNA.	hSPRY1	0.00866466	-10.4439
ILMN_2219683	GNMT	6	Homo sapiens glycine N-methyltransferase (GNMT), mRNA.		0.000233221	-10.5656
ILMN_1657435	MANEA	6	Homo sapiens mannosidase, endo-alpha (MANEA), mRNA.	FLJ12838; hEndo; DKFZp	1.96E-08	-15.9823
ILMN_1679357	MGST1	12	Homo sapiens microsomal glutathione S-transferase 1 (MGST1), transcript variant 1c, mRNA.	GST12; MGST-I; MGC145	0.0142824	-17.316
ILMN_1808732	LPAR1	9	Homo sapiens lysophosphatidic acid receptor 1 (LPAR1), transcript variant 2, mRNA.	vzg-1; Gpcr26; Mrec1.3;	8.21E-14	-19.1059
ILMN_1701017	DAB2	5	Homo sapiens disabled homolog 2, mitogen-responsive phosphoprotein (Drosophila) (DAB2), mRNA.	FLJ26626; DOC2; DOC-2	6.25E-11	-23.8836
ILMN_1708183	HPS5	11	Homo sapiens Hermansky-Pudlak syndrome 5 (HPS5), transcript variant 2, mRNA.	KIAA1017; AIBP63	0.0404888	-24.7939

ILMN_2124802	CPN1	10	Homo sapiens carboxypeptidase N, polypeptide 1 (CPN1), mRNA.	CPN; SCPN; FLJ40792	0.000381143	-30.5616
ILMN_1787266	DMKN	19	Homo sapiens dermokine (DMKN), transcript variant 2, mRNA.	ZD52F10; UNQ729	0.0049283	-33.4115
ILMN_2102721	S100A4	1	Homo sapiens S100 calcium binding protein A4 (S100A4), transcript variant 2, mRNA.	FSP1; MTS1; 18A2; PEL9	0.0213645	-35.8629
ILMN_2193213	DEFB1	8	Homo sapiens defensin, beta 1 (DEFB1), mRNA.	DEFB-1; BD1; HBD1; DEF	0.0194302	-36.4604
ILMN_1728262	COL6A3	2	Homo sapiens collagen, type VI, alpha 3 (COL6A3), transcript variant 1, mRNA.	DKFZp686K04147; FLJ34	8.23E-12	-36.6937
ILMN_1662880	C21orf33	21	Homo sapiens chromosome 21 open reading frame 33 (C21orf33), nuclear gene encoding mitochondrial protein, transcript variant 2, mRNA.	ES1; GT335; HES1; KNP-	1.01E-07	-37.9713
ILMN_1725661	FRMD6	14	Homo sapiens FERM domain containing 6 (FRMD6), transcript variant 2, mRNA.	EX1; c14_5320; MGC179	0.0193786	-40.8348
ILMN_2165289	HSD17B10	X	Homo sapiens hydroxysteroid (17-beta) dehydrogenase 10 (HSD17B10), nuclear gene encoding mitochondrial protein, transcript variant 1, mRNA.	HCD2; ABAD; ERAB; MHE	0.0197241	-40.9638
